# Rifampicin resistance and associated factors of *Mycobacterium tuberculosis* among pulmonary tuberculosis-suspected patients in the Amhara National Regional State Comprehensive Specialized Hospitals, Ethiopia

**DOI:** 10.1371/journal.pone.0351964

**Published:** 2026-06-26

**Authors:** Tebelay Dilnessa, Feleke Moges, Belay Tessema, Workagegnehu Hailu, Baye Gelaw

**Affiliations:** 1 Department of Medical Microbiology, School of Biomedical and Laboratory Sciences, College of Medicine and Health Sciences, University of Gondar, Gondar, Ethiopia; 2 Department of Medical Laboratory Science, College of Medicine and Health Sciences, Debre Markos University, Debre Markos, Ethiopia; 3 Institute of Clinical Immunology, Faculty of Medicine, University of Leipzig, Leipzig, Germany; 4 Department of Internal Medicine, School of Medicine, College of Medicine and Health Sciences, University of Gondar, Gondar, Ethiopia; Yekatit 12 Hospital, ETHIOPIA

## Abstract

**Background:**

Tuberculosis (TB) remains a major public health challenge globally, with drug-resistant strains, particularly rifampicin-resistant *Mycobacterium tuberculosis* (RR-MTB), which poses serious challenges for treatment and control. Rifampicin resistance is widely recognized as a key surrogate marker for multidrug-resistant MTB and is associated with poor treatment outcomes and increased transmission risk. Ethiopia is among the high-TB-burden countries, and the Amhara National Regional State continues to report significant tuberculosis morbidity and mortality.

**Objective:**

To assess the prevalence, rifampicin resistance patterns, and associated factors of *M. tuberculosis* among pulmonary tuberculosis-suspected patients at the Amhara National Regional State Comprehensive Specialized Hospitals, Northwest Ethiopia.

**Methods:**

A multicenter prospective cross-sectional study was conducted among pulmonary tuberculosis-suspected patients attending the Comprehensive Specialized Hospitals from April 2023, to May 2025. Socio-demographic and associated factors data were collected using semi-structured questionnaires. Sputum was collected and tested using GeneXpert MTB/RIF assay to detect *M. tuberculosis* and rifampicin resistance. Data were entered into SPSS version 28 for analysis. Binary logistic regression was applied to assess the relationship between predictors and the prevalence of *M. tuberculosis,* and rifampicin resistance. Variables with a *p*-value < 0.05 at 95% confidence interval in multivariable logistic regression were considered statistically significant.

**Results:**

Among 2548 pulmonary tuberculosis (PTB)-suspected participants, the overall prevalence of *M. tuberculosis* (MTB) was 150/2548 (5.9%). Rifampicin resistance was detected in 19/150 (12.7%) of MTB-positive cases, urban residents 7/70 (18.6%), and low education (grades 1−4), 3/15 (20.0%). In multivariable logistic regression analysis, rural residence was associated with reduced PTB infection compared with urban residence (AOR: 0.23; 95% CI: 0.06–0.79; *p* = 0.020). Previous antibiotic use (AOR: 9.17; 95% CI: 2.01–28.14; *p* = 0.001), HIV-positivity (AOR: 3.73; 95% CI: 2.40–7.78; *p* = 0.001) and alcohol consumption (AOR: 7.10; 95% CI: 4.99–20.86; *p* = 0.001) were independently associated with increased MTB infection. Rural residents showed significantly lower odds of rifampicin-resistant MTB compared to urban counterparts (AOR: 0.35, 95% CI: 0.12–0.99, *p* = 0.048).

**Conclusion:**

The prevalence of *M. tuberculosis* infection and rifampicin resistance remains significant in the study area. The strong association between prior antibiotic use, HIV-positivity and alcohol consumption with *M. tuberculosis* highlights the need for targeted case-finding, improved diagnosis, rational antibiotic use, and focused health education interventions.

## Introduction

Tuberculosis (TB), caused by *Mycobacterium tuberculosis* (MTB), is a major cause of morbidity and mortality worldwide, particularly in low- and middle-income countries [1]. According to the World Health Organization (WHO), tuberculosis remains among the top 10 causes of death globally and the leading cause of death from a single infectious agent, surpassing HIV/AIDS [2]. The emergence of drug-resistant MTB, especially rifampicin-resistant MTB (RR-MTB) and multidrug-resistant tuberculosis (MDR-TB), has become a significant threat to global PTB control efforts [3].

Ethiopia is among the 30 high-TB and MDR-TB burden countries [2]. The Amhara National Regional State is one of the most populous administrative regions in Ethiopia that consistently reports a high number of PTB cases [4]. Delayed diagnosis and poor treatment adherence contribute to the development and spread of drug-resistant strains [5]. The region is one of the most TB-affected areas compounded by different contributing factors such as malnutrition, displacement, and instability. In recent years, the region reported increasing rates of both PTB and drug-resistant TB, underscoring the urgency for continued research and targeted interventions [6].

Rifampicin resistance is of particular concern due to its role as a key first-line anti-TB drug and its use as a proxy marker for MDR-TB. Rifampicin resistance in MTB remains a major public health challenge due to its strong correlation with MDR-TB. It poses a serious threat to tuberculosis control efforts due to limited treatment options and increased morbidity [2,7]. Accurate and rapid detection of MTB and its resistance to rifampicin is critical for the timely initiation of appropriate therapy and to prevent further transmission. Molecular diagnostic tools such as GeneXpert MTB/RIF facilitate rapid simultaneous detection of MTB and rifampicin resistance [8,9].

Several studies have documented that sociodemographic factors (such as low educational status, unemployment, and poor socioeconomic conditions), clinical factors (including HIV co-infection, previous tuberculosis treatment, and comorbidities), and behavioral characteristics (such as smoking, alcohol use, and treatment non-adherence) significantly influence the development and transmission of RR-MTB [7,10–13]. These associated factors often interact, leading to delayed diagnosis, incomplete treatment, and subsequent amplification of drug resistance. Multiple associated factors contribute to both PTB incidence and rifampicin resistance in Amhara National Regional State, Ethiopia [14].

In Ethiopia, PTB remains a leading cause of illness and death, with rifampicin resistance emerging as a major public health concern [15]. The prevalence of rifampicin resistance varies across regions, and both individual patient characteristics and broader contextual factors have been shown to contribute to its development [16]. In the Amhara National Regional State, where the dual burden of high PTB prevalence and increasing rifampicin resistance exists, the need for focused research is particularly critical.

Despite the availability of diagnostic tools, there is still limited evidence that comprehensively examines the determinants of rifampicin resistance in this region, especially among PTB-suspected patients attending Comprehensive Specialized Hospitals. Generating such evidence is essential for informing targeted interventions, including patient education, strategies to improve treatment adherence, and tailored public health measures. Therefore, this study aimed to assess the prevalence of MTB infection and rifampicin resistance, and associated factors among PTB-suspected patients attending Comprehensive Specialized Hospitals in the Amhara National Regional State, Ethiopia.

## Materials and methods

### Study area and setting

The study was conducted among PTB-suspected patients at Comprehensive Specialized Hospitals in the Amhara National Regional State, Northwest Ethiopia. The region has eight Comprehensive Specialized Hospitals: University of Gondar Comprehensive Specialized Hospital (UoGCSH), Dessie Comprehensive Specialized Hospital (DCSH), Felege Hiwot Comprehensive Specialized Hospital (FHCSH), Tibebe Ghion Comprehensive Specialized Hospital (TGCSH), Debre Markos Comprehensive Specialized Hospital (DMCSH), Woldia Comprehensive Specialized Hospital (WCSH), Debre Tabor Comprehensive Specialized Hospital (DTCSH), and Debre Berhan Comprehensive Specialized Hospital (DBCSH), all of which serve large catchment populations within the region [17].

Among these, three hospitals were selected for this study: DMCSH, FHCSH, and UoGCSH. These hospitals function as major referral centers and provide comprehensive diagnostic and treatment services for PTB patients, making them appropriate settings for assessing the prevalence of MTB and patterns of drug resistance. Furthermore, DMCSH, FHCSH and UoGCSH serve as treatment-initiating centers (TIC) for MDR-TB.

### Study design, population, variables, and eligibility criteria

A multicenter cross-sectional study was conducted from April 2023 to May 2025 at Comprehensive Specialized Hospitals in the Amhara National Regional State, Ethiopia. The study included patients aged ≥8 years who were suspected of having PTB and underwent GeneXpert testing using sputum samples during the study period.

The dependent variables were the prevalence of MTB and rifampicin resistance patterns. Independent variables included sociodemographic factors (age, sex, residence, educational status, employment status, income, and marital status) and clinical-related factors such as HIV status, family history of PTB, distance from the health facility, chronic illnesses, cigarette smoking, and alcohol consumption.

Patients with PTB confirmed by GeneXpert, complete clinical data, and adequate sputum samples were included. Patients with known PTB or on anti-TB treatment, those who had taken antibiotics within two weeks before sample collection, and critically ill patients unable to provide sputum samples were excluded from the study.

### Sample size and sampling technique

A total of 2548 PTB-suspected patients were enrolled using a consecutive sampling technique from three health facilities. Enrollment continued until a total of 150 MTB-positive cases were identified. To ensure balanced representation and enable comparison across study sites, recruitment at each facility proceeded until 50 bacteriologically confirmed tuberculosis cases were obtained per site.

### Operational definitions

#### Indeterminate rifampicin-resistant tuberculosis:

Is a GeneXpert MTB/RIF assay result in which MTB is detected, but the test cannot reliably determine rifampicin susceptibility due to ambiguous or unresolvable rpoB gene mutation signals, rendering the resistance status inconclusive [18,19].

#### Pulmonary tuberculosis-suspected patients:

Is a person who presents with symptoms or signs suggestive of tuberculosis, most commonly a productive cough lasting ≥ 2 weeks, which may be accompanied by fever, weight loss, night sweats, chest pain, or hemoptysis and who were referred for diagnostic evaluation using GeneXpert [20].

#### Previous antibiotic use:

Refers to the history of taking any antibiotic medications within 6 months, regardless of whether the drugs were prescribed by a health professional or obtained without a prescription.

#### Rifampicin-resistant *M. tuberculosis* (RR-MTB):

It is defined as *M. tuberculosis* isolates in which resistance to rifampicin is detected by the GeneXpert MTB/RIF assay, where resistance is determined by detection of mutations in the rpoB gene [21,22].

### Data collection procedures and GeneXpert MTB/RIF assay processing

Socio-demographic and clinical data were collected using a semi-structured, pre-tested questionnaire prepared from the WHO guideline [23] and other trusted sources [13,24,25] and administered through face-to-face interviews by trained healthcare personnel. Data included were age, sex, residence, source of drinking water, HIV status, history of contact with PTB patients, alcohol consumption, and cigarette smoking habit.

Sputum was collected using a sterile, leak-proof, wide-mouthed 50 mL falcon tube from each patient. Patients were trained on the importance of high-quality sputum specimen and instructed on proper sputum expectoration to ensure that deep respiratory samples were obtained rather than saliva.

The GeneXpert MTB/RIF assay was run according to the manufacturer’s instructions. The assay detects both MTB DNA and mutations in the rpoB gene associated with rifampicin resistance. The GeneXpert assay is based on real-time polymerase chain reaction (PCR) technology, which detects the DNA of MTB complex and mutations in the rpoB gene associated with resistance to rifampicin [26]. Two mL sputum samples were mixed with 4 mL buffer, vortex mixed, and incubated at room temperature for 15 minutes. Two milliliters of the homogenized sample were then transferred into the GeneXpert cartridge, which was inserted into the machine after barcode registration. Results were generated within 90 minutes.

### Data analysis and interpretations

Data were entered and analyzed using SPSS version 28 computer software. Descriptive statistics were used to present, organize, summarize, and interpret the data. Binary logistic regression analysis was employed to identify factors associated with PTB infection and rifampicin resistance. Multicollinearity among the independent variables was assessed using the Variance Inflation Factor (VIF) before multivariable logistic regression analysis, with a VIF value < 5 indicating no significant multicollinearity. Variables with a *p-*value less than or equal to 0.25 in the univariable logistic regression were jointly entered into a multivariable logistic regression analysis. A *p*-value less than 0.05 with 95% confidence intervals (CI) were considered significantly associated with MTB and/or rifampicin resistance. Model fitness was evaluated using the Hosmer-Lemeshow goodness-of-fit test.

### Ethical considerations

The study was conducted in accordance with the principles of the Declaration of Helsinki. Ethical clearance was obtained from the Institutional Review Board (IRB) of the College of Medicine and Health Sciences of the University of Gondar (Ref. number: CMHS/SH/CS/CoMEng/06/216/3/2015). Written permission was obtained from Debre Markos, Felege-Hiwot, and the University of Gondar Comprehensive Specialized Hospitals for the data collection process. Written informed assent and consent were obtained from the parents/guardians and study participants, respectively. Confidentiality and anonymity were also maintained, and participants received counseling and appropriate referral when necessary.

## Results

### Socio-demographic characteristics of the study participants

A total of 2548 PTB-suspected patients were enrolled in this study, of whom 1358 (53.3%) were females. The dominant age group was 16–30 years 939 (36.9%), followed by 41–65 years 526 (20.6%). In terms of residence, 1008 (39.5%) were urban residents and 1540 (60.5%) were from rural areas. Concerning the study area, 904 (35.5%) were from FHCSH, 862 (33.8%) from DMCSH, and 782 (30.7%) from the UoGCSH. Data on the employment status, 453 (17.8%) were formally employed, 1654 (64.9%) were informally employed, and 441 (17.3%) were unemployed (Table 1).

**Table 1:** Socio-demographic characteristics, proportion of MTB and rifampicin resistance pattern by GeneXpert MTB/RIF assay among PTB-suspected patients in the Amhara National Regional State Comprehensive Specialized Hospitals, Ethiopia.

Variables	Categories of variables	Tuberculosis status (N = 2548)				Rifampicin susceptibility pattern (N = 150)			
		Positive, n (%)	95%CI	Negative, n (%)	Total, n (%)	Detected, n (%)	Not detected, n (%)	Indeterminate, n (%)	Total, n (%)
Sex	Male	76 (6.4)	5.0-7.8	1114 (93.6)	1190 (46.7)	8 (10.5)	62 (81.6)	6 (7.9)	76 (50.7)
	Female	74 (5.4)	4.2-6.7	1284 (94.6)	1358 (53.3)	11 (14.8)	58 (78.4)	5 (6.8)	74 (49.3)
Age group (in years)	8-15	22 (6.1)	3.6-8.6	339 (93.9)	361 (14.2)	1 (4.6)	18 (81.8)	3 (13.6)	19 (14.7)
	16-30	32 (3.4)	2.3-4.6	907 (96.6)	939 (36.9)	4 (12.5)	27 (84.4)	1 (3.1)	31 (21.3)
	31-40	28 (6.6)	4.2-8.9	398 (93.4)	426 (16.7)	6 (21.4)	22 (78.6)	0 (0.0)	28 (18.7)
	41-65	35 (6.7)	4.5-8.8	491 (93.3)	526 (20.6)	4 (11.4)	28 (80.0)	3 (8.6)	35 (23.3)
	> 66	33 (11.2)	7.6-14.7	263 (88.8)	296 (11.6)	4 (12.1)	25 (75.8)	4 (12.1)	33(22.0)
Residence	Urban	70 (7.0)	5.4-8.5	938 (93.0)	1008 (39.5)	13 (18.6)	52 (74.3)	5 (7.1)	70 (46.7)
	Rural	80 (5.2)	4.1-6.3	1460 (94.8)	1540 (60.5)	6 (7.5)	68 (85.0)	6 (7.5)	80 (53.3)
Study area	University of Gondar CSH	50 (6.4)	4.7-8.1	732 (93.6)	782 (30.7)	7 (14.0)	39 (78.0)	4 (8.0)	50 (33.4)
	Felege-Hiwot CSH	50 (5.5)	4.0-7.0	854 (94.5)	904 (35.5)	6 (12.0)	41 (82.0)	3 (6.0)	50 (33.3)
	Debre Markos CSH	50 (5.8)	4.2-7.4	812 (94.2)	862 (33.8)	6 (12.0)	40 (80.0)	4 (8.0)	50 (33.3)
Educational status	No formal education	43 (6.0)	4.3-7.8	671 (94.0)	714 (28.0)	4 (9.3)	37 (86.0)	2 (4.7)	43 (28.7)
	Grade 1–4	15 (3.3)	1.6-4.9	446 (96.7)	461 (18.1)	3 (20.0)	11 (73.3)	1 (6.7)	15 (10.0)
	Grade 5–8	24 (4.7)	2.8-6.5	490 (95.3)	514 (20.2)	4 (16.7)	18 (75.0)	2 (8.3)	24 (16.0)
	Grade 9–12	27 (7.1)	4.5-9.7	353 (92.9)	380 (14.9)	4 (14.8)	20 (74.1)	3 (11.1)	27 (18.0)
	College or above	41 (8.6)	6.1-11.1	438 (91.4)	479 (18.8)	4 (9.8)	34 (82.9)	3 (7.3)	41 (27.3)
Employment status	Formally employed	33 (7.3)	4.9-9.7	420 (92.7)	453 (17.8)	5 (15.2)	27 (18.8)	1 (3.0)	33 (22.0)
	Informally employed	104 (6.3)	5.1-7.5	1550 (93.7)	1654 (64.9)	14 (13.5)	83 (79.8)	7 (6.7)	104 (69.3)
	Unemployed	13 (3.0)	1.4-4.5	428 (97.0)	441 (17.3)	0 (0.0)	10 (77.0)	3 (23.0)	13 (8.7)
Family size	< 5	60 (6.2)	4.7-7.8	901 (93.8)	961 (37.7)	12 (20.0)	46 (76.7)	2 (3.3)	60 (40.0)
	> 5	90 (5.7)	4.5-6.8	1497 (94.3)	1587 (62.3)	7 (7.8)	74 (82.2)	9 (10.0)	90 (60.0)
Income (ETB)	< 3,000	44 (5.2)	3.7-6.6	809 (94.8)	853 (33.5)	6 (13.6)	33 (75.0)	5 (11.4)	44 (29.3)
	3,001-6,000	77 (5.4)	4.3-6.6	1339 (94.6)	1416 (55.6)	6 (7.8)	66 (85.7)	5 (6.5)	77 (51.4)
	> 6,001	29 (10.4)	6.8-14.0	250 (89.6)	279 (10.9)	7 (24.1)	21 (72.4)	1 (3.5)	29 (19.3)
Marital status	Currently married	91 (7.3)	5.8-8.7	1163 (92.7)	1254 (49.2)	16 (17.6)	70 (76.9)	5 (5.5)	91 (60.7)
	Single	48 (4.1)	2.9-5.2	1133 (95.9)	1181 (46.4)	3 (6.3)	41 (85.4)	4 (8.3)	48 (32.0)
	Ever-married	11 (9.7)	4.3−15.2	102 (90.3)	113 (4.4)	0 (0.0)	9 (81.8)	2 (19.2)	11 (7.3)
Total		150 (5.9)	5.0- 6.9	2398 (94.1)	2548 (100)	19 (12.7, 7.4-18.0)	120 (80.0, 73.6-86.4)	11 (7.3)	150 (100)

CSH: Comprehensive Specialized Hospitals; ETB: Ethiopian Birr

### Prevalence of *M. tuberculosis*

The overall prevalence of MTB among the PTB-suspected patients was 150/2548 (5.9%; 95%CI: 5.0–6.9). The prevalence of MTB was 76/1190 (6.4%) in males and 74/1358 (5.4%) in females. The highest prevalence by age group was observed among participants ≥ 66 years 33/296 (11.2%) followed by those aged 41–65 years 35/526 (6.7%). Participants from urban areas had a higher prevalence of MTB 70/1008 (7.0%) compared to those from rural areas 80/1540 (5.2%). The prevalence of MTB was 50/782 (6.4%) in the UoGCSH, 50/904 (5.5%) in FHCSH, and 50/862 (5.8%) in DMCSH. On the other hand, the prevalence of MTB was 41/479 (8.6%) among patients who had college or above, but 43/714 (6.0%) among no formal education. The prevalence of TB among formally and informally employed participants was 33/453 (7.3%) and 104/1654 (6.3%), respectively (Table 1).

### Prevalence of rifampicin-resistant *M. tuberculosis*

Among the 150 MTB-positive patients tested by GeneXpert MTB/RIF assay, rifampicin resistance was detected in 19/150 (12.7%; 95% CI: 7.4–18.0), while 120/150 (80.0%; 95% CI: 73.6–86.4) were rifampicin susceptible, and 11/150 (7.3%; 95% CI: 3.2–11.5) yielded indeterminate results. Rifampicin resistance was 11/74 (14.8%) in females and 8/76 (10.5%) in males. Across age groups, the highest rifampicin resistance was observed among patients aged 31–40 years 6/28 (21.4%) followed by those aged 16–30 years 4/31 (12.5%). Urban residents had a higher resistance rate 13/70 (18.6%) than rural residents 6/80 (7.5%). The prevalence of rifampicin resistance was 7/50 (14.0%) in the UoGCSH, 6/50 (12.0%) in FHCSH, and 6/50 (12.0%) in DMCSH (Table 1).

### Clinical and behavioral characteristics of PTB-suspected patients

Among the 2548 PTB-suspected patients, the most frequently reported chief complaint was cough 1086 (42.6%), followed by fever 700 (27.5%) and chest pain 471 (18.5%), while hemoptysis was relatively uncommon 39 (1.5%), but had a higher proportion of positivity 4 (10.3%). Regarding comorbid conditions, 320 (12.5%) of participants were HIV-positive, among whom 52 (16.2%) were PTB-positive. Behavioral and exposure-related factors showed notable differences: PTB-positivity was higher among cigarette smokers 39 (19.0%) and alcohol drinkers 68 (30.5%) compared to non-users (111 (4.7%) and 82 (3.5%), respectively) (Table 2).

**Table 2:** Clinical and behavioral characteristics of PTB-suspected patients in the Amhara National Regional State Comprehensive Specialized Hospitals, Ethiopia (N = 2548).

Variables		*M. tuberculosis* status		Total, n (%)
		Positive, n (%)	Negative, n (%)	
Chief compliant	Cough	86 (7.9)	1000 (92.1)	1086 (42.6)
	Fever	46 (6.6)	654 (93.4)	700 (27.5)
	Chest pain	6 (1.3)	465 (98.7)	471 (18.5)
	Weight loss	3 (1.8)	168 (98.2)	171 (6.7)
	Hemoptysis	4 (10.3)	35 (89.7)	39 (1.5)
	Shortness of breath	5 (6.2)	76 (93.8)	81 (3.2)
HIV status	Yes	52 (16.2)	268 (83.8)	320 (12.5)
	No	98 (4.4)	2130 (95.6)	2228 (84.5)
Diabetes	Yes	18 (3.4)	511 (96.6)	529 (20.8)
	No	132 (6.5)	1887 (93.5)	2019 (79.2)
Asthma	Yes	19 (3.7)	494 (96.3)	513 (20.1)
	No	131 (6.4)	1904 (93.6)	2035 (79.9)
Hypertension	Yes	10 (8.9)	102 (91.1)	112 (4.4)
	No	140 (5.7)	2296 (94.3)	2436 (95.6)
Previous treatment history pneumonia	Yes	16 (7.6)	194 (92.4)	210 (8.2)
	No	134 (5.7)	2204 (94.3)	2338 (91.8)
Previous antibiotics use	Yes	77 (7.6)	943 (92.4)	1020 (40.0)
	No	73 (4.8)	1455 (95.2)	1528 (60.0)
Source of drinking water	Tap water	70 (6.9)	942 (93.1)	1012 (39.7)
	River and others	80 (5.2)	1456 (94.8)	1536 (60.3)
Presence of PTB patient in the family	Yes	82 (8.0)	939 (92.0)	1021 (40.0)
	No	68 (4.5)	1459 (95.5)	1527 (60.0)
Cigarette smoking	Yes	39 (19.0)	166 (81.0)	205 (8.0)
	No	111 (4.7)	2232 (95.3)	2343 (92.0)
Alcohol drinking habit	Yes	68 (30.5)	155 (69.5)	223 (8.8)
	No	82 (3.5)	2243 (96.5)	2325 (91.2)
Distance from home to HCF	< 40 km	70 (6.9)	942 (93.1)	1012 (39.7)
	> 40 km	80 (5.2)	1456 (94.8)	1536 (60.3)
Actions taken for the first appearance of symptoms	Self-medication	39 (5.0)	742 (95.0)	781 (30.6)
	Traditional medicine	47 (6.8)	648 (93.2)	695 (27.3)
	Holly water	18 (3.9)	442 (96.1)	460 (18.1)
	Consult HCWs locally	20 (6.8)	272 (93.2)	292 (11.5)
	Consult HCWs at HCF	26 (8.1)	294 (91.9)	320 (12.5)
**Total**		**150 (5.9)**	**2398 (94.1)**	**2548 (100)**

HCW: Healthcare worker, HCF: Healthcare facility, HIV: Human immunodeficiency virus

### Sociodemographic factors associated with PTB infection

In the univariable binary logistic regression analysis, age, educational status, employment status, income, marital status, and residence were associated with MTB infection (*p* < 0.25). In the multivariable logistic regression analysis, age, residence, employment status, income, and marital status remained associated with MTB infection. Rural residence was associated with reduced odds of MTB infection compared with urban residence (AOR: 0.23; 95% CI: 0.06–0.79; *p* = 0.020). Informal employment was also associated with higher odds of infection (AOR: 2.40; 95% CI: 1.20–4.86; *p* = 0.015), while lower income (less than 3,000 ETB) was linked to decreased odds (AOR: 0.54; 95% CI: 0.31–0.96; *p* = 0.036). Additionally, being single was associated with lower likelihood of infection compared to currently married individuals (AOR: 0.51; 95% CI: 0.34–0.81; *p* = 0.004). Other variables, including educational status and sex, were not significantly associated with MTB infection after adjustment for confounders (p > 0.05) (Table 3).

**Table 3:** Bivariable and multivariable logistic regression analysis of socio-demographic factors for MTB infection among PTB-suspected patients in the Amhara National Regional State Comprehensive Specialized Hospitals, Ethiopia (N = 2548).

Variables		*M. tuberculosis* status		COR (95% CI)	*P*-value	AOR (95% CI)	*P*-value
		Positive, n (%)	Negative, n (%)				
Sex	Male	76 (6.4)	1114 (93.6)	1.18 (0.85-1.65)	0.316		
	Female	74 (5.4)	1284 (94.6)	1			
Age group (in years)	8-15	22 (6.1)	339 (93.9)	1		1	
	16-30	32 (3.4)	907 (96.6)	0.54 (0.31-0.95)	0.032^*^	0.09 (0.04-0.20)	0.001^**^
	31-40	28 (6.6)	398 (93.4)	1.08 (0.61-1.93)	0.784	0.13 (0.06-0.30)	0.001^**^
	41-65	35 (6.7)	491 (93.3)	1.09 (0.63-1.90)	0.738	0.14 (0.06-0.32)	0.001^**^
	> 66	33 (11.2)	263 (88.8)	1.93 (1.10-3.39)	0.022^*^	0.33 (0.15-0.70)	0.004^**^
Residence	Urban	70 (7.0)	938 (93.0)	1		1	
	Rural	80 (5.2)	1460 (94.8)	0.73 (0.53-1.02)	0.067^*^	0.23 (0.06-0.79)	0.020^**^
Study area	University of Gondar CSH	50 (6.4)	732 (93.6)	1.11 (0.74-1.66)	0.615		
	Felege-Hiwot CSH	50 (5.5)	854 (94.5)	0.95 (0.63-1.42)	0.807		
	Debre Markos CSH	50 (5.8)	812 (94.2)	1			
Educational status	No formal education	43 (6.0)	671 (94.0)	0.68 (0.44-1.07)	0.095^*^	0.63 (0.26-1.54)	0.316
	Grade 1–4	15 (3.3)	446 (96.7)	0.36 (0.19-0.66)	0.001^*^	0.42 (0.16-1.07)	0.069
	Grade 5–8	24 (4.7)	490 (95.3)	0.52 (0.31-0.88)	0.015^*^	0.54 (0.25-1.20)	0.130
	Grade 9–12	27 (7.1)	353 (92.9)	0.82 (0.49-1.35)	0.434	0.86 (0.42-1.73)	0.673
	College & above	41 (8.6)	438 (91.4)	1		1	
Employment status	Formally employed	33 (7.3)	420 (92.7)	1		1	
	Informally employed	104 (6.3)	1550 (93.7)	0.85 (0.51-1.28)	0.446	2.40 (1.20-4.86)	0.015^**^
	Unemployed	13 (3.0)	428 (97.0)	0.38 (0.20-0.74)	0.005^*^	0.45 (0.21-1.41)	0.213
Family size	< 5	60 (6.2)	901 (93.8)	1			
	> 5	90 (5.7)	1497 (94.3)	0.90 (0.64-1.26)	0.552		
Income (ETB)	< 3,000	44 (5.2)	809 (94.8)	0.47 (0.28-0.76)	0.002^*^	0.54 (0.31-0.96)	0.036^**^
	3,001-6,000	77 (5.4)	1339 (94.6)	0.49 (0.32-0.77)	0.002^*^	1.53 (0.48-4.82)	0.465
	> 6,001	29 (10.4)	250 (89.6)	1		1	
Marital status	Currently married	91 (7.3)	1163 (92.7)	1		1	
	Single	48 (4.1)	1133 (95.9)	0.54 (0.37-0.77)	0.001^*^	0.51 (0.34-0.81)	0.004^**^
	Ever-married	11 (9.7)	102 (90.3)	1.38 (0.71-2.66)	0.339	1.1 (0.54-2.14)	0.847

CSH: Comprehensive Specialized Hospitals; AOR: Adjusted odds ratio; COR: Crude odds ratio; ^*^: candidate variables for multivariable logistic regression (*p*<0.25); ^**^: significantly associated variables *(p*<0.05)

### Association of clinical and behavioral factors with PTB infection

In the univariable binary logistic regression analysis, several variables were associated with MTB infection among PTB-suspected patients. These included HIV status, diabetes mellitus, asthma, hypertension, previous antibiotic use, source of drinking water, presence of a PTB patient in the family, cigarette smoking, alcohol drinking habit, distance from home to health care facility, and the first action taken following symptom onset (*p* < 0.25).

In the multivariable logistic regression analysis, HIV-positive status (AOR: 3.73; 95% CI: 2.40–7.78; *p* = 0.001), previous antibiotic use (AOR: 9.17; 95% CI: 2.01–28.14; *p* = 0.001), and alcohol drinking (AOR: 7.10; 95% CI: 4.99–20.86; *p* = 0.001) remained independently associated with increased odds of MTB infection. Other variables, including diabetes, asthma, hypertension, cigarette smoking, family history of PTB, source of drinking water, distance to health facility, and initial healthcare-seeking behaviors, were not significantly associated with MTB infection after adjustment for confounders (*p* > 0.05) (Table 4).

**Table 4:** Bivariate and multivariable logistic regression analysis of associated factors for MTB infection among PTB-suspected patients in the Amhara National Regional State Comprehensive Specialized Hospitals, Ethiopia (N = 2548).

Variables		*M. tuberculosis* status		COR (95% CI)	*P*-value	AOR (95% CI)	*P*-value
		Positive, n (%)	Negative, n (%)				
HIV status	Yes	52 (16.2)	268 (83.8)	4.22 (2.94-6.04)	0.001^*^	3.73 (2.40-7.78)	0.001^**^
	No	98 (4.4)	2130 (95.6)	1		1	
Diabetes	Yes	18 (3.4)	511 (96.6)	0.50 (0.30-0.83)	0.007^*^	0.66 (0.37-1.17)	0.154
	No	132 (6.5)	1887 (93.5)	1		1	
Asthma	Yes	19 (3.7)	494 (96.3)	0.56 (0.34-0.91)	0.020^*^	0.57 (0.32-1.00)	0.051
	No	131 (6.4)	1904 (93.6)	1		1	
Hypertension	Yes	10 (8.9)	102 (91.1)	1.61 (0.82-3.15)	0.166^*^	1.48 (0.67.3.29)	0.326
	No	140 (5.7)	2296 (94.3)	1		1	
Previous pneumonia treatment history	Yes	16 (7.6)	194 (92.4)	1.36 (0.79-2.32)	0.267		
	No	134 (5.7)	2204 (94.3)	1			
Previous antibiotics use	Yes	77 (7.6)	943 (92.4)	1.63 (1.17-2.26)	0.004^*^	9.17 (2.01-28.14)	0.001^**^
	No	73 (4.8)	1455 (95.2)	1		1	
Source of drinking water	Tap water	70 (6.9)	942 (93.1)	1		1	
	River and others	80 (5.2)	1456 (94.8)	0.74 (0.53-1.03)	0.074^*^	4.50 (0.42-26.32)	0.997
Presence of PTB patient in the family	Yes	82 (8.0)	939 (92.0)	1.87 (1.34-2.61)	0.001^*^	0.02 (0.01-10.53)	0.998
	No	68 (4.5)	1459 (95.5)	1		1	
Cigarette smoking	Yes	39 (19.0)	166 (81.0)	4.72 (3.17-7.03)	0.001^*^	1.01 (0.56-1.83)	0.961
	No	111 (4.7)	2232 (95.3)	1		1	
Alcohol drinking habit	Yes	68 (30.5)	155 (69.5)	12.0 (8.37-17.20)	0.001^*^	7.10 (4.99-20.86)	0.001^**^
	No	82 (3.5)	2243 (96.5)	1		1	
Distance from home to HCF	< 40 km	70 (6.9)	942 (93.1)	1		1	
	> 40 km	80 (5.2)	1456 (94.8)	0.74 (0.53-1.03)	0.074^*^	2.35 (0.78-10.57)	0.691
Actions taken for the first appearance of symptoms	Self-medication	39 (5.0)	742 (95.0)	0.59 (0.35-0.99)	0.047^*^	0.83 (0.36-1.90)	0.653
	Traditional medicine	47 (6.8)	648 (93.2)	0.82 (0.49-1.35)	0.436	1.19 (0.62-2.32)	0.598
	Holly water	18 (3.9)	442 (96.1)	0.46 (0.25-0.85)	0.014^*^	0.71 (0.32-1.57)	0.397
	Consult HCWs locally	20 (6.8)	272 (93.2)	0.83 (0.45-1.52)	0.550	1.70 (0.51-5.64)	0.385
	Consult HCWs at HCF	26 (8.1)	294 (91.9)	1		1	

AOR: Adjusted odds ratio; COR: Crude odds ratio; HCW: Health care workers; HCF: Health care facility; *: candidate variables for multivariable logistic regression (*p*<0.25); **: significantly associated variables *(p*<0.05).

### Associated factors for rifampicin-resistant *M. tuberculosis*

In the bivariable logistic regression analysis, residence, alcohol consumption, and previous antibiotic use showed associations with RR-MTB among MTB-patients (*p* < 0.25). However, in the multivariable logistic regression analysis, only residence remained significantly associated with RR-MTB, with rural residents being less likely to develop resistance compared to urban counterparts (AOR: 0.35, 95% CI: 0.12–0.99, *p* = 0.048). Other variables, including sex, age group, employment status, cigarette smoking, HIV status, alcohol consumption, and previous antibiotic use, were not significantly associated with RR-MTB in the adjusted model (*p* > 0.05) (Table 5).

**Table 5:** Bivariable and multivariable logistic regression analysis of associated factors for rifampicin resistant-MTB among MTB-patients in the Amhara National Regional State Comprehensive Specialized Hospitals, Ethiopia (N = 139^a^).

Variables		Rifampicin resistant pattern		COR (95% CI)	*P*-value	AOR (95% CI)	*P*-value
		Resistant, n (%)	Susceptible, n (%)				
Sex	Male	8 (11.4)	62 (88.6)	0.68 (0.25-1.81)	0.440		
	Female	11 (16.0)	58 (84.0)	1			
Age group (in years)	8-15	1 (5.3)	18 (94.7)	1			
	16-30	4 (13.0)	27 (87)	2.66 (0.27-25.83)	0.397		
	31-40	6 (21.4)	22 (78.6)	4.90 (0.54-44.60)	0.158		
	41-65	4 (12.5)	28 (87.5)	2.57 (0.26-44.88)	0.415		
	> 66	4 (13.8)	25 (86.2)	2.88 (0.29-27.97)	0.362		
Residence	Urban	13 (20.0)	52 (80.0)	1		1	
	Rural	6 (8.1)	68 (91.9)	0.35 (0.12-0.99)	0.048^*^	0.35 (0.12-0.99)	0.048^**^
Employment status	Formally employed	5 (15.6)	27 (84.4)	1			
	Informally employed	14 (14.4)	83 (85.6)	0.91 (0.30-2.76)	0.869		
	Unemployed	0 (0.0)	10 (100)	0.05 (0.01-10.50)	0.987		
Alcohol consumption	Yes	9 (13.6)	57 (86.4)	2.83 (1.01-7.95)	0.048^*^	0.78 (0.28-2.13)	0.629
	No	10 (13.7)	63 (86.3)	1		1	
Cigarette smoking	Yes	6 (15.8)	32 (84.2)	1.27 (0.44-3.62)	0.656		
	No	13 (12.9)	88 (87.1)	1			
HIV status	Yes	5 (10.6)	42 (89.4)	0.66 (0.22-1.97)	0.459		
	No	14 (15.2)	78 (84.8)	1			
Previous antibiotics use	Yes	13 (18.6)	57 (81.4)	2.39 (0.85-6.72)	0.097^*^	0.84 (0.24-13.22)	0.945
	No	6 (8.7)	63 (91.3)	1		1	

CSH: Comprehensive Specialized Hospitals; AOR: Adjusted odds ratio; COR: Crude odds ratio, ^*^: candidate variables for multivariable logistic regression (*p*<0.25); ^**^: significantly associated variables *(p*<0.05); ^a^: Indeterminate MTB isolates (n=11) were excluded from associated factors analysis.

## Discussion

The overall prevalence of MTB among PTB-suspected patients was, 5.9% and RR-MTB was detected among 12.7% of the confirmed cases. HIV-positive status, prior antibiotic use, and alcohol consumption significantly affected by PTB infection. Urban residents showed significantly lower odds of rifampicin resistance compared to rural counterparts. The prevalence of MTB was slightly lower than reports such as the 8.4% documented in Ethiopia [27]. A multi-year GeneXpert program data reported 10.5% positivity among 17,615 presumptive TB patients from 2015 to 2021 [28]. A 2024 year trend analysis from Northwest Ethiopia found 11.7% positivity in 3,696 presumptive cases, again higher than the current study [29]. Other single-site studies likewise report higher yields: 12.7% in a 2023 cross-sectional study using GeneXpert, 17.2% in a 2025 analysis of 273 presumptive patients, and 26.8% in a smaller facility-based series [14,30,31]. The lower prevalence of MTB in the present study compared with previous Ethiopian reports may be due to differences in the study population, as the current multi-center design may dilute positivity compared to earlier single-center studies focusing on high-risk groups such as defaulters and lost follow-up patients. Variations in geographic TB burden, improved TB control interventions, and expanded GeneXpert access may also have reduced positivity rates. In addition, differences in diagnostic practices, sputum quality, and health-seeking behavior, as well as temporal declines in TB transmission and variation in HIV co-infection rates, may further explain the observed discrepancy.

The magnitude of MTB in the current study falls within the range reported in the Ethiopian population surveys, which estimate bacteriologically-confirmed PTB prevalence near 147 per 100,000 population [32], although much higher rates have also been seen depending on case definition and diagnostic method. Ethiopia’s national TB prevalence survey reported 277 per 100,000 bacteriologically confirmed TB, while subnational studies have shown variation across regions [33,34]. Localized studies in hotspot regions and high-risk occupational groups, such as miners, report notably higher prevalence, exceeding 7% or more in specific subpopulations [32]. Demographically, the finding of marginally higher prevalence in males aligns with national and regional data, where men consistently show elevated notification and incidence rates, with male-to-female ratios up to 1.4 [33,35]. Differences in PTB prevalence across studies often reflect variations in case mix and inclusion criteria, diagnostic methods and their quality, epidemiological context, and temporal or programmatic factors. The lower prevalence observed in the current study likely reflects its broader catchment, which included a large proportion of low-risk presumptive cases and excluded retreatment patients, unlike earlier single-site studies.

The urban MTB prevalence rate (7.0%) versus rural (5.2%) slightly contrasts with some earlier Ethiopian surveys, which show greater PTB notification in rural settings, largely a reflection of healthcare access and reporting; however, escalating urbanization and overcrowding are increasingly driving higher urban PTB rates. Urban residence was significantly associated with increased MTB detection, possibly due to higher population density, transmission dynamics, and better case detection in urban settings [27]. Inter-site differences (Gondar, Bahir Dar, and Debre Markos) reflect spatial clustering documented in the Amhara Regional State and other regions, where migration and local socioeconomic factors influence local PTB rates [34]. Education-wise, higher PTB prevalence among illiterate individuals is echoed in a Tanzanian study, where both extremes of educational attainment carried a higher risk, potentially due to different exposure patterns, awareness, or mobility [36]. Informally employed was significantly associated with PTB in the current study. The finding that informally employed individuals were significantly associated with PTB is consistent with previous peer-reviewed studies conducted in low-income settings [37,38]. Informally employed individuals are more likely to work in crowded workplaces, public transport systems, markets, or migratory labor settings where TB transmission is facilitated, whereas unemployed individuals may have relatively reduced exposure to such high-contact environments.

The PTB prevalence was highest among individuals with higher income (>6,001 ETB), indicating that the risk of PTB does not necessarily decrease with increasing income. This finding is consistent with evidence from urban Ethiopia, where higher TB notification rates were linked to increased mobility, healthcare-seeking behavior, population density, and greater social interaction rather than poverty alone [39]. Similarly, a national study from South Korea reported that tuberculosis was not confined to economically disadvantaged populations and that socioeconomic patterns of TB can vary across different settings, particularly in urbanized communities with complex social mixing patterns [40]. The higher MTB prevalence among higher-income participants in this study may be related to increased health-care utilization and earlier access to diagnostic services, which increases the probability of being tested and confirmed compared with lower-income groups who may delay care or remain undiagnosed. It may also reflect occupational and urban-related exposure differences, as higher-income individuals are more likely to live or work in densely populated settings where transmission risk is higher. With respect to marital status, the magnitudes of MTB were 9.7% and 4.1% among ever-married and single participants, respectively. The elevated prevalence among ever-married individuals has been documented elsewhere and may reflect social vulnerability, reduced support networks, and related health challenges which was supported by a literature [41]. The lower PTB prevalence among single individuals compared with currently married participants can be explained by single individuals are often younger and may have had shorter cumulative exposure time to MTB compared with married individuals, who are generally older and have longer lifetime exposure.

The magnitude of MTB with respect to educational attainment was 28% among no formal education and only 18.8% having college-level education or higher, which parallels reports from other Ethiopian settings: for example, a study identified low education as a major associated factor for PTB clustering, particularly among internal migrants and in hotspots with high rural population density [42]. Income distributions in PTB studies across Ethiopia and Kenya typically reveal that most patients earn under local median incomes, emphasizing the links between poverty and PTB risk [43]. A comparative study in Kenya and other parts of Sub-Saharan Africa also highlights a concentration of PTB cases among young adults, males, and those of low socioeconomic and educational status. The clustering of PTB in densely populated, lower-income, and rural areas remains a consistent theme, with migration and poor living conditions frequently cited as amplifying factors [44].

Among the 150 MTB-positive patients tested by GeneXpert MTB/RIF assay, rifampicin resistance was detected in 12.7% of cases. The rifampicin resistance rate was relatively consistent across study centers: Gondar (14%), Bahir Dar (12%), and Debre Markos (12%). A study in Pakistan among pediatric PTB patients found 4.5% RR-MTB, a rate lower than the 12.7% reported here, reflecting possible differences in population and settings [45]. A study in Southwest Ethiopia found rifampicin resistance at 3.4%, which is lower than the current study aligning with the increased resistance seen in certain age and urban groups [46]. Another Ethiopian study reported rifampicin resistance at 9.8%, [47] which is consistent with the current study (12.7%). A study reviewing a larger population showed a declining trend, but still reported notable resistance levels in both adults and children, with 8.3% rifampicin resistance among adults and 7.2% in children, somewhat lower but comparable to the current findings [48]. In the current study, a relatively high proportion of indeterminate results (7.3%) was observed among MTB during rifampicin susceptibility testing using the GeneXpert MTB/RIF assay. This may be attributed to low bacillary load in clinical specimens, suboptimal sample quality, the presence of PCR inhibitors, instrument- or cartridge-related issues, and mutations occurring outside the rifampicin resistance-determining region (RRDR).

The finding that only residence remained independently associated with RR-MTB, with rural residents having lower odds compared to urban counterparts, is consistent with evidence suggesting that urban settings often facilitate the transmission and amplification of drug-resistant MTB due to higher population density, overcrowding, and greater exposure to previously treated or inadequately managed TB cases. Studies conducted in Ethiopia and other high-burden settings have similarly reported increased RR-MTB prevalence in urban populations, likely reflecting better diagnostic access, but also higher rates of treatment interruption, informal healthcare use, and antibiotic misuse in cities [49,50]. The protective effect observed among rural residents may reflect lower exposure to drug-resistant strains due to reduced population density, limited transmission networks, and less frequent prior tuberculosis treatment compared to urban settings, where overcrowding, higher healthcare contact, and antibiotic misuse are more common drivers of resistance.

In this study, the prevalence of PTB among suspected cases was influenced by several socio-demographic characteristics [51–53]. Previous antibiotic use, HIV-positive status, and alcohol drinking were significantly associated with PTB. This finding is consistent with a study from Debre Markos Referral Hospital, which reported a significant association between previous antibiotic use and PTB-prevalence [24]. Similarly, studies from Bahir Dar city and Sekota town identified alcohol consumption, and HIV-seropositivity as important predictors [54,55]. Additionally, studies from Adigrat General Hospital and Gedeo zone demonstrated that HIV coinfection was significantly associated with RR-MTB among MTB-positive cases [56,57]. Alcohol drinking remained significant in the current study, supported by previous Ethiopian and international evidences linking alcohol use with increased PTB susceptibility and adverse outcomes [49,58–60]. These associated factors may contribute to a higher prevalence of PTB due to their combined effects on weakening immune function, delaying accurate diagnosis and treatment, promoting poor treatment adherence, and increasing susceptibility to opportunistic infections and drug-resistant MTB.

### Limitations of the study

The use of the GeneXpert MTB/RIF assay, detects only rifampicin-resistant and not resistance to other first-line or second-line anti-MTB drugs. The use of convenience sampling techniques may limit the generalizability of the findings. The referral site data also cause selection bias and overestimation of RR- MTB. Information on socio-demographic and clinical factors was collected using a face-to-face interview, which could be subjected to recall bias and social desirability bias.

## Conclusion and recommendation

The prevalence of MTB among PTB-suspected cases was 5.9%, with higher rates observed among older age groups and urban residents. Rifampicin resistance was observed in 12.7% of MTB-positives. Pulmonary tuberculosis was significantly associated with previous antibiotic use, HIV-positivity and alcohol consumption. Strengthening early detection of MTB and expanding routine rifampicin resistance testing are essential, particularly among high-risk groups such as individuals living with HIV. Interventions should promote appropriate antibiotic use, and reduce alcohol consumption. Focused health education and ongoing surveillance of drug-resistant MTB are needed to limit transmission and improve PTB control in the region.

## Supporting information

S1 Data Raw data set in excel. (XLSX)

## Abbreviations

DMCSH: Debre Markos Comprehensive Specialized Hospital
FHCSH: Felege-Hiwot Comprehensive Specialized Hospital
MDR-TB: Multidrug-resistant tuberculosis
PTB: Pulmonary tuberculosis
RR-MTB: Rifampicin-resistant *M. tuberculosis*
UoGCSH: University of Gondar Comprehensive Specialized Hospital.
