# Supplementary material for: Rifampicin resistance and associated factors of Mycobacterium tuberculosis among pulmonary tuberculosis-suspected patients in the Amhara National Regional State Comprehensive Specialized Hospitals, Ethiopia
Source: PLoS One. 2026 Jun 26;21(6):e0351964. doi: 10.1371/journal.pone.0351964 (PMC13309019; doi:10.1371/journal.pone.0351964)
Supplement: Attachment — Submitted filename: PONE-D-26-17510.pdf [file pone.0351964.s004.pdf]

PLOS One

Rifampicin resistance and associated factors of Mycobacterium tuberculosis among pulmonary tuberculosis-suspected patients in the Amhara National Regional State Comprehensive Specialized Hospitals, Ethiopia

--Manuscript Draft--

**Manuscript Number:**	PONE-D-26-17510
**Article Type:**	Research Article
**Full Title:**	Rifampicin resistance and associated factors of Mycobacterium tuberculosis among pulmonary tuberculosis-suspected patients in the Amhara National Regional State Comprehensive Specialized Hospitals, Ethiopia
**Short Title:**	Rifampicin resistance of Mycobacterium tuberculosis among pulmonary tuberculosis-suspected patients
**Corresponding Author:**	Tebelay Dilnessa, MSc<br>University of Gondar<br>Debre Markos, ETHIOPIA
**Keywords:**	Tuberculosis, Rifampicin resistance, Mycobacterium tuberculosis, GeneXpert
**Abstract:**	Background: Tuberculosis (TB) remains a major public health challenge globally, with drug-resistant strains, particularly rifampicin-resistant Mycobacterium tuberculosis (RR-MTB), which poses serious challenges for treatment and control. Ethiopia is among the high TB burden countries, and the Amhara National Regional State continues to report significant tuberculosis morbidity and mortality.<br>Objective: To determine the prevalence of rifampicin-resistant M. tuberculosis and associated factors among tuberculosis-suspected patients at the Amhara National Regional State Comprehensive Specialized Hospitals, Northwest Ethiopia.<br>Methods: A multicenter cross-sectional study was conducted among pulmonary tuberculosis-suspected patients attending comprehensive specialized hospitals from April 01, 2023, to May 30, 2025. Socio-demographic and associated factor data were collected using semi-structured questionnaires. Sputum was collected and tested using GeneXpert MTB/RIF assay to detect M. tuberculosis and rifampicin resistance. Data were entered into SPSS version 28 for analysis. Binary logistic regression was applied to assess the relationship between predictors and the outcome variable. Variables with p-value of ≤ 0.05 at 95%CI in multivariate logistic regression were considered statistically significant.<br>Results: Among 2,548 pulmonary tuberculosis (PTB)-suspected participants, the overall prevalence of M. tuberculosis was 150/2548 (5.9%). The prevalence was higher in males (6.4%) than females (5.4%), in urban (7.0%) compared to rural (5.2%) residents, and among participants aged > 66 years (11.2%). Rifampicin resistance was detected in 19/150 (12.7%) of M. tuberculosis-positive cases, more frequently among urban residents (18.6%), and among clients with low education 20.0% (in grades 1-4). In multivariate logistic regression analysis, age, residence, occupation, income, and marital status were associated with M. tuberculosis infection (p< 0.05). Rural residence was associated with reduced pulmonary tuberculosis infection compared with urban residence (AOR: 0.23; 95%CI: 0.06-0.79). Students had markedly lower odds of PTB (AOR: 0.03; 95%CI: 0.01-0.11). Previous antibiotic use (AOR: 9.17; 95%CI: 2.01-28.14) and alcohol consumption (AOR: 7.10; 95%CI: 4.99-20.86) were independently associated with increased M. tuberculosis infection.<br>Conclusion: The prevalence of M. tuberculosis infection (5.9%) and rifampicin resistance (12.7%) remain significant in the study area. The strong association between prior antibiotic use and alcohol consumption with M. tuberculosis highlights the need for targeted case-finding, improved diagnosis, rational antibiotic use, and focused health education interventions.
**Order of Authors:**	Tebelay Dilnessa, MSc
	Feleke Moges, PhD, Professor
	Belay Tessema, PhD, Professor
	Workagegnehu Hailu, MD+, Associate Professor

Powered by Editorial Manager® and ProduXion Manager® from Aries Systems Corporation

	Baye Gelaw, PhD, Professor
Additional Information:	
Question	Response
**Financial Disclosure**<br><br>Enter a financial disclosure statement that describes the sources of funding for the work included in this submission. Review the submission guidelines for detailed requirements. View published research articles from *PLOS ONE* for specific examples.<br><br>This statement is required for submission and **will appear in the published article** if the submission is accepted. Please make sure it is accurate.<br><br>**Funded studies**<br>Enter a statement with the following details:<br>• Initials of the authors who received each award<br>• Grant numbers awarded to each author<br>• The full name of each funder<br>• URL of each funder website<br>• Did the sponsors or funders play any role in the study design, data collection and analysis, decision to publish, or preparation of the manuscript?<br><br>Did you receive funding for this work?	The author(s) received no specific funding for this work.
**Competing Interests**<br><br>Use the instructions below to enter a competing interest statement for this submission. On behalf of all authors, disclose any competing interests that could be perceived to bias this work—acknowledging all financial support and any other relevant financial or non-financial competing interests.<br><br>This statement is required for submission and **will appear in the published article** if the submission is accepted. Please make sure it is accurate and that any funding	The authors have declared that no competing interests exist.

sources listed in your Funding Information later in the submission form are also declared in your Financial Disclosure statement.<br><br>View published research articles from *PLOS ONE* for specific examples.<br><br>**NO authors have competing interests**<br><br>Enter: *The authors have declared that no competing interests exist.*<br><br>**Authors with competing interests**<br><br>Enter competing interest details beginning with this statement:<br><br>*I have read the journal's policy and the authors of this manuscript have the following competing interests: [insert competing interests here]*<br><br>* typeset	
**Ethics Statement**<br><br>Enter an ethics statement for this submission. This statement is required if the study involved:<br><br>• Human participants<br>• Human specimens or tissue<br>• Vertebrate animals or cephalopods<br>• Vertebrate embryos or tissues<br>• Field research<br><br>Write "N/A" if the submission does not require an ethics statement.<br><br>General guidance is provided below. Consult the submission guidelines for detailed instructions. **Make sure that all information entered here is included in the Methods section of the manuscript.**	N/A

**Format for specific study types**<br><br>**Human Subject Research (involving human participants and/or tissue)**<br>• Give the name of the institutional review board or ethics committee that approved the study<br>• Include the approval number and/or a statement indicating approval of this research<br>• Indicate the form of consent obtained (written/oral) or the reason that consent was not obtained (e.g. the data were analyzed anonymously)<br><br>**Animal Research (involving vertebrate animals, embryos or tissues)**<br>• Provide the name of the Institutional Animal Care and Use Committee (IACUC) or other relevant ethics board that reviewed the study protocol, and indicate whether they approved this research or granted a formal waiver of ethical approval<br>• Include an approval number if one was obtained<br>• If the study involved *non-human primates*, add *additional details* about animal welfare and steps taken to ameliorate suffering<br>• If anesthesia, euthanasia, or any kind of animal sacrifice is part of the study, include briefly which substances and/or methods were applied<br><br>**Field Research**<br><br>Include the following details if this study involves the collection of plant, animal, or other materials from a natural setting:<br>• Field permit number<br>• Name of the institution or relevant body that granted permission	
Data Availability<br><br>Before publication, Authors are required to make fully available and without restriction all data underlying their findings. Sharing the data underlying your findings increases the visibility, credibility, and impact of your work, while supporting transparency and future scientific discovery. Please see our PLOS Data Policy page for detailed information on this policy.</p><br><br>A Data Availability	All relevant data are within the manuscript and its supporting information files.

Powered by Editorial Manager® and ProduXion Manager® from Aries Systems Corporation

Statement, detailing where the data can be accessed, is required at first submission. Insert your Data Availability Statement in the box below.</p><br><br>Please see the data reporting of our submission guidelines for instructions on what you need to include in your Data Availability Statement. Examples of acceptable Data Availability Statements are included here for reference:</p><br> <br> - The minimal data set [and accompanying code, where generated] is available at [repository name] via [DOI hyperlink] <br> - No data was generated by this study. The following existing data sources were used: [dataset name] from [dataset location] available via [web address or DOI hyperlink]. <br> - For Study Protocols: No datasets were generated or analysed during the current study. All relevant data from this study will be made available upon study completion. <br> <br><br><i>PLOS allows rare exemptions to address legal and ethical concerns. If you have legal or ethical restrictions, please use the box below to detail these in full sentences for the Journal team to consider.</i></p><br><br>This statement will be typeset if the manuscript is accepted for publication.</p>	

Manuscript Click here to access/download;Manuscript;Manuscript.docx

Rifampicin resistance and associated factors of *Mycobacterium tuberculosis* among pulmonary tuberculosis-suspected patients in the Amhara National Regional State Comprehensive Specialized Hospitals, Ethiopia

Tebelay Dilnessa [a, b]*, Feleke Moges [a], Belay Tessema [a, c], Workagegnehu Hailu [d], Baye Gelaw [a]

[a] Department of Medical Microbiology, School of Biomedical and Laboratory Sciences, College of Medicine and Health Sciences, University of Gondar; P. O. Box 196, Gondar, Ethiopia

[b] Department of Medical Laboratory Science, College of Medicine and Health Sciences, Debre Markos University; P. O. Box 269, Debre Markos, Ethiopia

[c] Institute of Clinical Immunology, Faculty of Medicine, University of Leipzig; 04103, Leipzig, Germany

[d] Department of Internal Medicine, School of Medicine, College of Medicine and Health Sciences, University of Gondar; P. O. Box 196, Gondar, Ethiopia

*Corresponding author: Tebelay Dilnessa: tebelay@gmail.com, Telephone: +251912198715

Abstract

Background: Tuberculosis (TB) remains a major public health challenge globally, with drug-resistant strains, particularly rifampicin-resistant *Mycobacterium tuberculosis* (RR-MTB), which poses serious challenges for treatment and control. Ethiopia is among the high TB burden countries, and the Amhara National Regional State continues to report significant tuberculosis morbidity and mortality.

Objective: To determine the prevalence of rifampicin-resistant *M. tuberculosis* and associated factors among tuberculosis-suspected patients at the Amhara National Regional State Comprehensive Specialized Hospitals, Northwest Ethiopia.

Methods: A multicenter cross-sectional study was conducted among pulmonary tuberculosis-suspected patients attending comprehensive specialized hospitals from April 01, 2023, to May 30, 2025. Socio-demographic and associated factors data were collected using semi-structured questionnaires. Sputum was collected and tested using GeneXpert MTB/RIF assay to detect *M. tuberculosis* and rifampicin resistance. Data were entered into SPSS version 28 for analysis. Binary logistic regression was applied to assess the relationship between predictors and the outcome variable. Variables with *p*-value of ≤ 0.05 at 95%CI in multivariate logistic regression were considered statistically significant.

Results: Among 2,548 pulmonary tuberculosis (PTB)-suspected participants, the overall prevalence of *M. tuberculosis* was 150/2548 (5.9%). The prevalence was higher in males (6.4%) than females (5.4%), in urban (7.0%) compared to rural (5.2%) residents, and among participants aged ≥ 66 years (11.2%). Rifampicin resistance was detected in 19/150 (12.7%) of *M. tuberculosis*-positive cases, more frequently among urban residents (18.6%), and among clients with low education 20.0% (in grades 1-4). In multivariate logistic regression analysis, age, residence, occupation, income, and marital status were associated with *M. tuberculosis* infection ($p < 0.05$). Rural residence was associated with reduced pulmonary tuberculosis infection compared with urban residence (AOR: 0.23; 95%CI: 0.06-0.79). Students had markedly lower odds of PTB (AOR: 0.03; 95%CI: 0.01-0.11). Previous antibiotic use (AOR: 9.17; 95%CI: 2.01-28.14) and alcohol consumption (AOR: 7.10; 95%CI: 4.99-20.86) were independently associated with increased *M. tuberculosis* infection.

Conclusion: The prevalence of *M. tuberculosis* infection (5.9%) and rifampicin resistance (12.7%) remain significant in the study area. The strong association between prior antibiotic use and alcohol consumption with *M. tuberculosis* highlights the need for targeted case-finding, improved diagnosis, rational antibiotic use, and focused health education interventions.

Keywords: Tuberculosis, Rifampicin resistance, *Mycobacterium tuberculosis*, GeneXpert

1. Introduction

Tuberculosis (TB), caused by *Mycobacterium tuberculosis* (Mtb), is a major cause of morbidity and mortality worldwide, particularly in low- and middle-income countries. According to the World Health Organization (WHO), TB remains among the top 10 causes of death globally and the leading cause of death from a single infectious agent, surpassing HIV/AIDS [1]. The emergence of drug-resistant *M. tuberculosis*, especially rifampicin-resistant TB (RR-TB) and multidrug-resistant TB (MDR-TB), has become a significant threat to global PTB control efforts [2].

Ethiopia is among the 30 high TB and MDR-TB burden countries [1]. The Amhara National Regional State is one of the most populous administrative regions in Ethiopia that consistently reported high number of PTB cases [3]. Delayed diagnosis and poor treatment adherence contribute to the development and spread of drug-resistant strains [4]. The region is one of the most TB-affected areas compounded with different contributing factors such as malnutrition, displacement and instability. In recent years, the region reported increasing rates of both PTB and drug-resistant TB underscoring the urgency for continued research and targeted interventions [5].

Rifampicin resistance is of particular concern due to its role as a key first-line anti-TB drug and its use as a proxy marker for MDR-TB. Rifampicin resistance in *M. tuberculosis* remains a major public health challenge due to its strong correlation with multidrug-resistant tuberculosis (MDR-TB). It poses a serious threat to TB control efforts due to limited treatment options and increased morbidity [1, 6]. Accurate and rapid detection of *M. tuberculosis* and its resistance to rifampicin is critical for timely initiation of appropriate therapy and to prevent further transmission. Molecular diagnostic tools such as GeneXpert MTB/RIF facilitate rapid simultaneous detection of *M. tuberculosis* and rifampicin resistance [7, 8].

Several studies have documented that sociodemographic factors (such as low educational status, unemployment, and poor socioeconomic conditions), clinical factors (including HIV co-infection, previous TB treatment, and comorbidities), and behavioral characteristics (such as smoking, alcohol use, and treatment non-adherence) significantly influence the development and transmission of rifampicin-resistant *M. tuberculosis* [9]. These associated factors often interact, leading to delayed diagnosis, incomplete treatment, and subsequent amplification of drug resistance. Multiple associated factors contribute to both TB incidence and rifampicin resistance in Amhara National Regional State, Ethiopia [10].

In Ethiopia, pulmonary tuberculosis (PTB) remains a leading cause of illness and death, with rifampicin resistance emerging as a major public health concern. The prevalence of rifampicin resistance varies across

regions, and both individual patient characteristics and broader contextual factors have been shown to contribute to its development [11]. In the Amhara National Regional State, where the dual burden of high PTB prevalence and increasing rifampicin resistance exists, the need for focused research is particularly critical.

Despite the availability of diagnostic tools, there is still limited evidence that comprehensively examines the determinants of rifampicin resistance in this region, especially among PTB-suspected patients attending comprehensive specialized hospitals. Generating such evidence is essential for informing targeted interventions, including patient education, strategies to improve treatment adherence, and tailored public health measures. Therefore, this study aimed to determine the prevalence of *M. tuberculosis* infection and rifampicin resistance, and to identify associated factors among PTB-suspected patients attending selected comprehensive specialized hospitals in the Amhara National Regional State, Ethiopia.

2. Material and methods

1.2. Study area and setting

The study was conducted among pulmonary tuberculosis-suspected patients at selected comprehensive specialized hospitals of the Amhara National Regional State, Northwest Ethiopia. The hospitals included in the study were Debre Markos Comprehensive Specialized Hospital, Felege-Hiwot Comprehensive Specialized Hospital and University of Gondar Comprehensive Specialized Hospital which serve as referral centers for a wide catchment population. Additionally, these hospitals provide diagnostic and treatment services for PTB patients, making them ideal settings for assessing *M. tuberculosis*-prevalence and resistance patterns.

2.2. Study design and period

A multifacility-based cross-sectional study was conducted from April 01, 2023, to May 30, 2025.

2.3. Study population

The study population consisted of patients aged ≥ 8 years who were suspected of having pulmonary tuberculosis and referred for GeneXpert testing during the study period. Pulmonary tuberculosis suspicion was based on national guidelines, including persistent cough, weight loss, fever, and night sweats.

2.4. Study variables

2.4.1. Dependent variables

- Prevalence of *M. tuberculosis*
- Rifampicin susceptibility patterns of *M. tuberculosis*

2.4.2. Independent variables

- Sociodemographic variables: Age, sex, residence (urban/rural), educational status, occupation

- Clinical and health-related factors: HIV status, health facility visited, distance from health facility, history of PTB patient in the family and contact history, presence of chronic illnesses (e.g., diabetes, hypertension, malnutrition), smoking history, and alcohol consumption

2.5. Eligibility criteria

2.5.1. Inclusion criteria

- Individuals diagnosed with PTB based on clinical, and laboratory findings (GeneXpert).
- Patients with complete clinical data and who provided adequate sputum samples.
- Patients aged greater than or equal to 8 years attending at the Amhara National Regional State Comprehensive Specialized Hospitals.

2.5.2. Exclusion criteria

- Patients with extrapulmonary TB or those on multi-drug-resistant TB (MDR-TB) treatment.
- Patients who have received antibiotics including anti-TB drugs within the past two weeks prior to sample collection.
- Patients who were critically ill and unable to provide sputum sample.

2.6. Sample size determination and sampling technique

The sample size was determined by enrolling participants consecutively until 150 *M. tuberculosis*-positive cases were obtained from the three health facilities. To ensure balanced representation and facilitate comparison across study sites, 50 *M. tuberculosis*-positive participants were included from each health facility.

Operational definitions

Multidrug-resistant tuberculosis (MDR-TB): Defined as *M. tuberculosis* that is resistant to at least isoniazid and rifampin, the two most potent tuberculosis drugs [12].

2.7. Data collection and processing

2.7.1. Sociodemographic data collection

Socio-demographic and clinical data were collected using a semi-structured and pre-tested questionnaire administered by trained healthcare personnels. Information included were age, sex, residence, TB history, HIV status, contact history, alcohol drinking habit, health facility visited, and smoking status.

2.7.2. Sputum sample collection

Sputum was collected using a sterile, leak-proof, wide-mouthed 50 mL falcon tube from each participant. Patients were trained on the importance of high-quality sputum specimen and instructed to collect the sputum sample. Emphasis was given on the need to produce sputum originating from the lungs, rather than saliva.

2.7.3. Laboratory investigation

The GeneXpert MTB/RIF assay was run according to the manufacturer's instructions. The assay detects both *M. tuberculosis* DNA and mutations in the rpoB gene associated with rifampicin resistance. The GeneXpert assay is based on real-time polymerase chain reaction (PCR) technology, which detects the DNA of *M. tuberculosis* complex and mutations in the rpoB gene associated with resistance to rifampicin [13]. Two mL sputum samples were mixed with 4 mL buffer, vortex mixed, and incubated at room temperature for 15 minutes. Two milliliters of the homogenized sample was then transferred into the GeneXpert cartridge, which was inserted into the machine after barcode registration. Results were generated within 90 minutes.

2.8. Data analysis and interpretations

Data were entered and analyzed using SPSS version 28 computer software. Descriptive data analysis was conducted to organize, summarize, and interpret the data. Bivariable logistic regression analysis was employed to examine the association between independent variables with tuberculosis or rifampicin resistance. A p-value ≤ 0.25 was used as a cut-off for inclusion into the multivariate model. Multicollinearity among the independent variables was assessed using the Variance Inflation Factor (VIF) prior to multivariable logistic regression analysis, with a VIF value < 5 indicating no significant multicollinearity. Variables with p-value less than or equal to 0.25 in the bivariate analysis were jointly entered into a multivariate logistic regression analysis. A p-value ≤ 0.05 with 95%CI was considered significantly associated with *M. tuberculosis* and/or rifampicin resistance. Model fitness was evaluated using the Hosmer-Lemeshow goodness-of-fit test.

2.9. Ethical considerations

The study was conducted in accordance with the principles of the Declaration of Helsinki. Ethical clearance was obtained from the Institutional Review Board (IRB) of College of Medicine and Health Sciences of University of Gondar (Ref. number: CMHS/SH/CS/CoMEng/06/216/3/2015). Written permission was obtained from Debre Markos, Felege-Hiwot and University of Gondar Comprehensive Specialized Hospitals for the data collection process. Written informed assent and consent were obtained from the parents/guardians and study participants, respectively. Confidentiality and anonymity were also maintained, and participants received counseling and appropriate referral when necessary.

3. Results

3.1. Socio-demographic characteristics of the study participants

A total of 2,548 tuberculosis-suspected participants were enrolled in this study, of whom 46.7% were males and 53.3% were females. The dominant age group was 16-30 years (36.9%), followed by 41-65 years (20.6%). The majority of participants were living in rural areas (60.5%), while 39.5% were from urban settings. In terms of study site distribution, 35.5% were from Felege-Hiwot, 33.8% from Debre Markos, and 30.7% from

University of Gondar Comprehensive Specialized Hospitals. Data on educational status of the study participants showed that 28.0% were illiterate, 20.2% completed elementary school, and 18.8% attained college or university. Farmers were predominant (40.8%) followed by merchants (20.3%), and government employees (17.8%). More than half of the study participants (62.3%) had a family size greater than five and the proportion of the study participants who had monthly income less than 3,000 Ethiopian birr (ETB) was 33.5%, those with 3,001-6,000 ETB (55.6%), and 10.9% earned above 6,001 ETB (Table 1).

Table 1: Socio-demographic characteristics and the proportion of *M. tuberculosis* among pulmonary tuberculosis-suspected participants in the Amhara National Regional State Comprehensive Specialized Hospitals, Ethiopia (N=2,548)

**Variables**	**Categories of variables**	**Tuberculosis status**			**Total, n (%)**
		**Positive, n (%)**	**95%CI**	**Negative, n (%)**	
Sex	Male	76 (6.4)	5.0-7.8	1114 (93.6)	1190 (46.7)
	Female	74 (5.4)	4.2-6.7	1284 (94.6)	1358 (53.3)
Age group (in years)	8-15	22 (6.1)	3.6-8.6	339 (93.9)	361 (14.2)
	16-30	32 (3.4)	2.3-4.6	907 (96.6)	939 (36.9)
	31-40	28 (6.6)	4.2-8.9	398 (93.4)	426 (16.7)
	41-65	35 (6.7)	4.5-8.8	491 (93.3)	526 (20.6)
	≥ 66	33 (11.2)	7.6-14.7	263 (88.8)	296 (11.6)
Residence	Urban	70 (7.0)	5.4-8.5	938 (93.0)	1008 (39.5)
	Rural	80 (5.2)	4.1-6.3	1460 (94.8)	1540 (60.5)
Study area	University of Gondar CSH	50 (6.4)	4.7-8.1	732 (93.6)	782 (30.7)
	Felege-Hiwot CSH	50 (5.5)	4.0-7.0	854 (94.5)	904 (35.5)
	Debre Markos CSH	50 (5.8)	4.2-7.4	812 (94.2)	862 (33.8)
Educational status	Illiterate	43 (6.0)	4.3-7.8	671 (94.0)	714 (28.0)
	Grade 1-4	15 (3.3)	1.6-4.9	446 (96.7)	461 (18.1)
	Grade 5-8	24 (4.7)	2.8-6.5	490 (95.3)	514 (20.2)
	Grade 9-12	27 (7.1)	4.5-9.7	353 (92.9)	380 (14.9)
	College & above	41 (8.6)	6.1-11.1	438 (91.4)	479 (18.8)
Occupation	Government employed	33 (7.3)	4.5-9.7	420 (92.7)	453 (17.8)
	Merchant	41 (7.9)	5.6-10.3	475 (92.1)	516 (20.3)
	Farmer	61 (5.9)	4.3-7.3	980 (94.1)	1041 (40.8)

	Daily laborer	2 (2.1)	0.8-5.0	92 (97.9)	94 (3.7)
	Student	4 (1.0)	0.2- 2.0	398 (99.0)	402 (15.8)
	Others	9 (21.4)	9.0-33.8	33 (78.6)	42 (1.6)
Family size	< 5	60 (6.2)	4.7-7.8	901 (93.8)	961 (37.7)
	≥ 5	90 (5.7)	4.5-6.8	1497 (94.3)	1587 (62.3)
Income (ETB)*	≤ 3,000	44 (5.2)	3.7-6.6	809 (94.8)	853 (33.5)
	3,001-6,000	77 (5.4)	4.3-6.6	1339 (94.6)	1416 (55.6)
	≥ 6,001	29 (10.4)	6.8-14.0	250 (89.6)	279 (10.9)
Marital status	Married	91 (7.3)	5.8-8.7	1163 (92.7)	1254 (49.2)
	Single	48 (4.1)	2.9-5.2	1133 (95.9)	1181 (46.4)
	Divorced	5 (10.6)	1.8-19.5	42 (89.4)	47 (1.8)
	Widowed	6 (9.1)	2.2-16.0	60 (90.9)	66 (2.6)
**Total**		**150 (5.9)**	**5.0- 6.9**	**2398 (94.1)**	**2548 (100.0)**

CSH: Comprehensive Specialized hospitals; ETB: Ethiopian Birr

3.2. Prevalence of *Mycobacterium tuberculosis*

The overall prevalence of *M. tuberculosis* among the participants was 5.9% (150/2,548) (5.0-6.9). The prevalence was slightly higher in males (6.4%) than in females (5.4%). The highest prevalence by age group was observed among participants ≥66 years (11.2%) followed by those aged 41-65 years (6.7%). Participants from urban areas had higher prevalence (7.0%) compared to those from rural areas (5.2%). The prevalence of *M. tuberculosis* infection was 6.4% in University of Gondar, 5.5% in Felege-Hiwot, and 5.8% in Debre Markos comprehensive specialized hospitals. On the other hand, the prevalence of *M. tuberculosis* infection was 8.6% among patients who had college and above education level, but 6.0% among illiterates. Data also showed that the prevalence of *M. tuberculosis* among divorced participants was 10.6% followed by the widowed (9.1%) (Table 1).

3.3. Rifampicin susceptibility pattern of *M. tuberculosis*

Among the 150 *M. tuberculosis* positive participants tested by GeneXpert MTB/RIF assay, rifampicin resistance was detected in 19 (12.7%; 95%CI: 7.4-18.0), while 120 (80.0%; 95%CI: 73.6-86.4) were rifampicin susceptible, and 11 (7.3%; 95%CI: 3.2-11.5) yielded indeterminate results. Rifampicin resistance was slightly higher in females (14.8%) compared to males (10.5%). Across age groups, the highest rifampicin resistance was observed among participants aged 31-40 years (21.4%) followed by those aged 16-30 years (12.5%). Urban residents had a higher resistance rate (18.6%) than rural residents (7.5%). The prevalence of rifampicin

resistance was 14.0% in University of Gondar, 12.0% in Felege-Hiwot and Debre Markos comprehensive specialized hospitals each. Data also showed that the proportion of rifampicin resistance was 20.0% among study participants who had lower educational status (grade 1-4) and 16.7% among those with grade 5-8, but 14.8% among grade 9-12 educational status. The prevalence of rifampicin-resistant *M. tuberculosis* was 21.9% among merchants but 15.0% among government employees. Married participants had higher proportion of rifampicin resistant *M. tuberculosis* (17.6%) compared to singles (6.3%) participants (Table 2).

Table 2: Frequency of rifampicin susceptibility pattern of *M. tuberculosis* using GeneXpert MTB/RIF assay among *M. tuberculosis*-positive participants in the Amhara National Regional State Comprehensive Specialized Hospitals, Ethiopia (N=150)

**Variables**	**Categories of variables**	**Rifampicin susceptibility pattern**			**Total, n (%)**
		**Detected, n (%)**	**Not detected, n (%)**	**Indeterminate, n (%)**	
Sex	Male	8 (10.5)	62 (81.6)	6 (7.9)	76 (50.7)
	Female	11 (14.8)	58 (78.4)	5 (6.8)	74 (49.3)
Age group (in years)	8-15	1 (4.6)	18 (81.8)	3 (13.6)	19 (14.7)
	16-30	4 (12.5)	27 (84.4)	1 (3.1)	31 (21.3)
	31-40	6 (21.4)	22 (78.6)	0 (0.0)	28 (18.7)
	41-65	4 (11.4)	28 (80.0)	3 (8.6)	35 (23.3)
	$\geq$ 66	4 (12.1)	25 (75.8)	4 (12.1)	33(22.0)
Residence	Urban	13 (18.6)	52 (74.3)	5 (7.1)	70 (46.7)
	Rural	6 (7.5)	68 (85.0)	6 (7.5)	80 (53.3)
Study area	University of Gondar CSH	7 (14.0)	39 (78.0)	4 (8.0)	50 (33.4)
	Felege-Hiwot CSH	6 (12.0)	41 (82.0)	3 (6.0)	50 (33.3)
	Debre Markos CSH	6 (12.0)	40 (80.0)	4 (8.0)	50 (33.3)
Educational status	Illiterate	4 (9.3)	37 (86.0)	2 (4.7)	43 (28.7)
	Grade 1-4	3 (20.0)	11 (73.3)	1 (6.7)	15 (10.0)
	Grade 5-8	4 (16.7)	18 (75.0)	2 (8.3)	24 (16.0)
	Grade 9-12	4 (14.8)	20 (74.1)	3 (11.1)	27 (18.0)
	College & above	4 (9.8)	34 (82.9)	3 (7.3)	41 (27.3)
Occupation	Government employed	5 (15.2)	27 (81.8)	1 (3.0)	33 (22.0)
	Merchant	9 (21.9)	28 (68.3)	4 (9.8)	41 (27.3)
	Farmer	4 (6.6)	54 (88.5)	3 (4.9)	61 (40.7)

	Daily laborer	1 (50.0)	1 (50.0)	0 (0.0)	2 (1.3)
	Student	0 (0.0)	3 (75.0)	1 (25.0)	4 (2.7)
	Others	0 (0.0)	7 (77.8)	2 (22.2)	9 (6.0)
Family size	< 5	12 (20.0)	46 (76.7)	2 (3.3)	60 (40.0)
	≥ 5	7 (7.8)	74 (82.2)	9 (10.0)	90 (60.0)
Income (ETB)	≤ 3,000	6 (13.6)	33 (75.0)	5 (11.4)	44 (29.3)
	3,001-6,000	6 (7.8)	66 (85.7)	5 (6.5)	77 (51.4)
	≥ 6,001	7 (24.1)	21 (72.4)	1 (3.5)	29 (19.3)
Marital status	Married	16 (17.6)	70 (76.9)	5 (5.5)	91 (60.7)
	Single	3 (6.3)	41 (85.4)	4 (8.3)	48 (32.0)
	Divorced	0 (0.0)	4 (80.0)	1 (20.0)	5 (3.3)
	Widowed	0 (0.0)	5 (83.3)	1 (16.7)	6 (4.0)
**Total**		**19 (12.7)**	**120 (80.0)**	**11 (7.3)**	**150 (100)**

CSH: Comprehensive Specialized hospitals; ETB: Ethiopian birr

3.4. Associated factors for tuberculosis infection

In the bivariate model, age, educational status, occupation, income, marital status, and residence were associated with *M. tuberculosis* infection. Data also showed that study participants with the age ≥66 years were 1.93 times more likely to be infected with *M. tuberculosis* compared with study participants aged 16-30 years (COR: 1.93; 95%CI: 1.10-3.39).

In the multivariate model, age, residence, occupation, income, and marital status remained associated with *M. tuberculosis* infection. Rural residence was associated with reduced odds of *M. tuberculosis* infection compared with urban residence (AOR: 0.23; 95%CI: 0.06-0.79). Students had markedly lower odds of *M. tuberculosis* infection (AOR: 0.03; 95%CI: 0.01-0.11) compared with government employed. Being single was also independently associated with lower PTB odds compared with being married (AOR: 0.51; 95%CI: 0.32-0.80) (Table 3).

235 **Table 3:** Bivariate and multivariate logistic regression analysis of socio-demographic factors against *M. tuberculosis* infection among tuberculosis-suspected participants in the Amhara National Regional Sate Comprehensive Specialized Hospitals, Ethiopia (N=2,548)

**Variables**		***M. tuberculosis* status**		**COR (95%CI)**	**P-value**	**AOR (95%CI)**	**P-value**
		**Positive, n (%)**	**Negative, n (%)**				
Sex	Male	76 (6.4)	1114 (93.6)	1.18 (0.85-1.65)	0.316		
	Female	74 (5.4)	1284 (94.6)	1			
Age group (in years)	8-15	22 (6.1)	339 (93.9)	1		1	
	16-30	32 (3.4)	907 (96.6)	0.54 (0.31-0.95)	0.032	0.06 (0.03-0.12)	0.001
	31-40	28 (6.6)	398 (93.4)	1.08 (0.61-1.93)	0.784	0.08 (0.04-0.17)	0.001
	41-65	35 (6.7)	491 (93.3)	1.09 (0.63-1.90)	0.738	0.08 (0.04-0.17)	0.001
	≥ 66	33 (11.2)	263 (88.8)	1.93 (1.10-3.39)	0.022	0.14 (0.06-0.32)	0.001
Residence	Urban	70 (7.0)	938 (93.0)	1			
	Rural	80 (5.2)	1460 (94.8)	0.73 (0.53-1.02)	0.067	0.23 (0.06-0.79)	0.020
Study area	University of Gondar CSH	50 (6.4)	732 (93.6)	1.11 (0.74-1.66)	0.615		
	Felege-Hiwot CSH	50 (5.5)	854 (94.5)	0.95 (0.63-1.42)	0.807		
	Debre Markos CSH	50 (5.8)	812 (94.2)	1			
Educational status	Illiterate	43 (6.0)	671 (94.0)	0.68 (0.44-1.07)	0.095	0.63 (0.26-1.54)	0.316
	Grade 1-4	15 (3.3)	446 (96.7)	0.36 (0.19-0.66)	0.001	0.42 (0.16-1.07)	0.069
	Grade 5-8	24 (4.7)	490 (95.3)	0.52 (0.31-0.88)	0.015	0.54 (0.25-1.20)	0.130
	Grade 9-12	27 (7.1)	353 (92.9)	0.82 (0.49-1.35)	0.434	0.86 (0.42-1.73)	0.673
	College & above	41 (8.6)	438 (91.4)	1		1	
Occupation	Government employed	33 (7.3)	420 (92.7)	1		1	
	Merchant	41 (7.9)	475 (92.1)	1.09 (0.68-1.77)	0.669	1.24 (0.72-2.14)	0.425
	Farmer	61 (5.9)	980 (94.1)	0.79 (0.51-1.23)	0.298	0.86 (0.44-1.71)	0.679
	Daily laborer	2 (2.1)	92 (97.9)	0.27 (0.06-1.12)	0.081	0.32 (0.07-1.47)	0.143
	Student	4 (1.0)	398 (99.0)	0.13 (0.04-0.36)	0.001	0.03 (0.01-0.11)	0.001
	Others	9 (21.4)	33 (78.6)	3.47 (1.53-7.86)	0.003	3.44 (1.28-9.27)	0.014

Family size	< 5	60 (6.2)	901 (93.8)	1			
	≥ 5	90 (5.7)	1497 (94.3)	0.90 (0.64-1.26)	0.552		
Income (ETB)	≤ 3,000	44 (5.2)	809 (94.8)	0.47 (0.28-0.76)	0.002	0.53 (0.29-0.94)	0.030
	3,001-6,000	77 (5.4)	1339 (94.6)	0.49 (0.32-0.77)	0.002	2.17 (0.65-7.30)	0.208
	≥ 6,001	29 (10.4)	250 (89.6)	1		1	
Marital status	Married	91 (7.3)	1163 (92.7)	1		1	
	Single	48 (4.1)	1133 (95.9)	0.54 (0.38-0.77)	0.001	0.51 (0.32-0.80)	0.004
	Divorced	5 (10.6)	42 (89.4)	1.52 (0.59-3.94)	0.387	1.38 (0.52-3.64)	0.513
	Widowed	6 (9.1)	60 (90.9)	1.27 (0.54-3.04)	0.579	0.74 (0.29-1.86)	0.521
**Total**		**150 (5.9)**	**2398 (94.1)**				

CSH: Comprehensive Specialized hospitals; AOR: Adjusted odds ratio; COR: Crude odds ratio

3.5. Association of clinical and behavioral factors with *M. tuberculosis* infection

In the bivariate model, several variables were significantly associated with *M. tuberculosis* infection among PTB-suspected patients. A higher likelihood of PTB was observed among participants with previous antibiotic use (COR: 1.63; 95%CI: 1.17-2.26; *p*= 0.004), and those who had a PTB patient in the family (COR: 1.87; 95%CI: 1.34-2.61; *p* = 0.001). Cigarette smoking (COR: 4.72; 95%CI: 3.17-7.03; *p*= 0.001) and alcohol drinking (COR: 12.00; 95%CI: 8.37-17.20; *p*= 0.001) were also strongly associated with PTB-positivity.

In the multivariate logistic regression analysis, previous antibiotic use (AOR: 9.17; 95%CI: 2.01-28.14; *p*= 0.001) and alcohol drinking (AOR: 7.10; 95%CI: 4.99-20.86; *p*= 0.001) remained independently associated with increased odds of *M. tuberculosis* infection. The type of health facility visited also remained significantly associated with PTB status, with lower odds observed among patients who visited health posts (AOR: 0.02; 95%CI: 0.05-0.12), health centers (AOR: 0.03; 95%CI: 0.06-0.15), government hospitals (AOR: 0.03; 95%CI: 0.07-0.17), and private hospitals/clinics (AOR: 0.03; 95% CI: 0.07-0.18) compared with other facilities (all *p*= 0.001). Other variables, including underlying diseases, cigarette smoking, distance from health facility, source of drinking water, and family history of *M. tuberculosis* infection were not statistically significant after adjustment (Table 4).

Table 4: Bivariate and multivariate regression analysis of associated factors against *M. tuberculosis* infection among tuberculosis-suspected patients in the Amhara National Regional State Comprehensive Specialized Hospitals, Ethiopia (N=2,548)

**Variables**		***M. tuberculosis* status**		**COR (95%CI)**	**P-value**	**AOR (95%CI)**	**P-value**
		**Positive, n (%)**	**Negative, n (%)**				
Chief compliant	Cough	86 (7.9)	1000 (92.1)	1.31 (0.51-3.32)	0.573	2.17 (0.75-6.30)	0.154
	Fever	46 (6.6)	654 (93.4)	1.07 (0.41-2.77)	0.891	1.77 (0.59-5.25)	0.305
	Chest pain	6 (1.3)	465 (98.7)	0.19 (0.06-0.66)	0.008	0.25 (0.05-0.87)	0.031
	Weight loss	3 (1.8)	168 (98.2)	0.27 (0.06-1.16)	0.079	0.43 (0.08-2.13)	0.304
	Hemoptysis	4 (10.3)	35 (89.7)	1.74 (0.45-6.86)	0.431	0.54 (0.09-3.33)	0.508
	Shortness of breath	5 (6.2)	76 (93.8)	1		1	
Underlying disease	HIV/AIDS	52 (15.5)	283 (84.5)	1.67 (0.77-3.63)	0.195	2.06 (0.82-5.20)	0.123
	Diabetes	18 (3.3)	532 (96.7)	0.69 (0.31-1.55)	0.377	0.89 (0.35-2.20)	0.813
	UTI	18 (3.7)	473 (96.3)	0.86 (0.39-1.88)	0.701	1.18 (0.46-2.98)	0.729
	Asthma	19 (3.7)	501 (96.3)	0.94 (0.43-2.04)	0.884	0.79 (0.31-1.97)	0.614
	Hypertension	10 (8.1)	113 (91.9)	2.09 (0.87-5.00)	0.097	1.92 (0.66-5.60)	0.230
	Pneumonia	16 (7.7)	193 (92.3)	1.48 (0.64-3.43)	0.351	1.97 (0.73-5.27)	0.178
	Others	8 (5.0)	151 (95.0)	0.89 (0.33-2.38)	0.824	1.49 (0.47-4.67)	0.494
	No underlying disease	9 (5.6)	152 (94.4)	1		1	
Previous antibiotics use	Yes	77 (7.7)	943 (92.5)	1.63 (1.17-2.26)	0.004	9.17 (2.01-28.14)	0.001
	No	73 (4.8)	1455 (95.2)	1		1	
Source of drinking water	Tap water	70 (6.9)	942 (93.1)	1		1	
	River and others	80 (5.2)	1456 (94.8)	0.74 (0.53-1.03)	0.074	4.50 (0.42-26.32)	0.997
Family history of PTB	Yes	82 (8.0)	939 (92.0)	1.87 (1.34-2.61)	0.001	0.02 (0.01-10.53)	0.998
	No	68 (4.5)	1459 (95.5)	1		1	
Cigarette smoking	Yes	39 (19.0)	166 (81.0)	4.72 (3.17-7.03)	0.001	1.01 (0.56-1.83)	0.961
	No	111 (4.7)	2232 (95.3)	1		1	
	Yes	68 (30.5)	155 (69.5)	12.0 (8.37-17.20)	0.001	7.10 (4.99-20.86)	0.001

Alcohol drinking habit	No	82 (3.5)	2243 (96.5)	1		1	
Distance from home to HCF	≤ 40 km	70 (6.9)	942 (93.1)	1		1	
	> 40 km	80 (5.2)	1456 (94.8)	0.74 (0.53-1.03)	0.074	2.35 (0.78-10.57)	0.691
Type of health facility visited	Health post	32 (5.7)	534 (94.3)	0.12 (0.03-0.50)	0.004	0.02 (0.01-0.12)	0.001
	Health center	45 (6.1)	687 (93.9)	0.13 (0.03-0.54)	0.005	0.03 (0.06-0.15)	0.001
	Gov’t hospital	43 (5.4)	754 (94.6)	0.11 (0.03-0.47)	0.003	0.03 (0.07-0.17)	0.001
	Private hospital/clinics	27 (6.1)	417 (93.9)	0.13 (0.03-0.54)	0.005	0.03 (0.07-0.18)	0.001
	Others	3 (33.3)	6 (66.7)	1		1	
Actions taken for the first appearance of symptoms	Self-medication	39 (5.0)	742 (95.0)	0.59 (0.35-0.99)	0.047	0.83 (0.36-1.90)	0.653
	Traditional medicine	47 (6.8)	648 (93.2)	0.82 (0.49-1.35)	0.436	1.19 (0.62-2.32)	0.598
	Holly water	18 (3.9)	442 (96.1)	0.46 (0.25-0.85)	0.014	0.71 (0.32-1.57)	0.397
	Consult HCWs locally	20 (6.8)	272 (93.2)	0.83 (0.45-1.52)	0.550	1.70 (0.51-5.64)	0.385
	Consult HCWs at HCF	26 (8.1)	294 (91.9)	1		1	
**Total**		**150 (5.9)**	**2398 (94.1)**				

AOR: Adjusted odds ratio; COR: Crude odds ratio; HCW: Health care workers; HCF: Health care facility

4. Discussion

The overall prevalence of *M. tuberculosis* among pulmonary tuberculosis (PTB)-suspected patients (5.9%; 150/2,548) is on the lower end of recent hospital-based Ethiopian findings. A multi-year GeneXpert program data reported 10.5% positivity among 17,615 presumptive tuberculosis patients from 2015 to 2021 [14]. A 2024 trend analysis from Northwest Ethiopia found 11.7% positivity in 3,696 presumptive cases, again higher than the current study [15]. Other single-site studies likewise report higher yields: 12.7% in a 2023 cross-sectional study using GeneXpert, 17.2% in 2025 analysis of 273 presumptive patients, and 26.8% in a smaller facility-based series [10, 16, 17].

The magnitude of *M. tuberculosis* in the current study falls within the range reported in the Ethiopian population surveys, which estimate bacteriologically-confirmed PTB prevalence near 147 per 100,000 population, although much higher rates have also been seen depending on case definition and diagnostic method. Localized studies in hotspot regions and high-risk occupational groups, such as miners, report notably higher prevalence, exceeding 7% or more in specific subpopulations [18]. Demographically, the finding of marginally higher prevalence in males aligns with national and regional data, where men consistently show elevated notification and incidence rates, with male-to-female ratios up to 1.4. The observed age gradient-with peak PTB prevalence among those at or above 66 years-matches the pattern seen across Ethiopia and Sub-Saharan Africa: older adults face higher PTB risk, while young adults constitute the majority of notified cases due to larger population size [19].

The urban *M. tuberculosis* prevalence rate (7.0%) versus rural (5.2%) slightly contrasts with some earlier Ethiopian surveys, which show greater PTB notification in rural settings, largely a reflection of healthcare access and reporting; however, escalating urbanization and overcrowding are increasingly driving for higher urban PTB rates. Inter-site differences (Gondar, Bahir Dar, Debre Markos) reflect spatial clustering documented in the Amhara Regional State and other regions, where migration and local socioeconomic factors influence local PTB rates [20]. Education-wise, higher PTB prevalence among illiterate individuals is echoed in a Tanzanian study, where both extremes of educational attainment carried higher risk-potentially due to different exposure patterns, awareness, or mobility. Occupational differences are consistent with regional literature: merchant, government employees, and farmers often show higher prevalence, while students and daily laborers tend to record the lowest rates-mirroring high PTB risks for those exposed to crowded or migratory work environments [21].

The observed patterns in economic and marital status-showing that PTB prevalence was highest among individuals with higher income (>6,001 ETB) and among divorced and widowed participants, indicate that the risk of PTB does not necessarily decrease with increasing income. This may be linked to factors such as urban residence and occupational exposures. Likewise, the elevated prevalence among divorced and widowed individuals has been documented elsewhere and may reflect social vulnerability, reduced support networks, and related health challenges [22].

The magnitude of *M. tuberculosis* with respect to educational attainment was 28% among illiterate and only 18.8% having college-level education or higher, parallels report from other Ethiopian settings: for example, identified low education as a major associated factor for PTB clustering, particularly among internal migrants and in hotspots with high rural population density. Occupational patterns show a similar picture elsewhere: farmers and day laborers carry greater PTB vulnerability due to socioeconomic challenges, frequent migration, and crowded living environments. Income distributions in PTB studies across Ethiopia and Kenya typically reveal most patients earning under local median incomes, emphasizing the links between poverty and PTB risk [23]. A comparative study in Kenya and other parts of Sub-Saharan Africa also highlights a concentration of PTB cases among young adults, males, and those of low socioeconomic and educational status. The clustering of PTB in densely populated, lower-income, and rural areas remains a consistent theme, with migration and poor living conditions frequently cited as amplifying factors [24].

Among the 150 *M. tuberculosis* positive participants tested with the GeneXpert MTB/RIF assay, rifampicin resistance (RR) was detected in 12.7% of cases. Rifampicin resistance was slightly higher among females (14.8%) compared to males (10.5%). Age-wise, the highest resistance was in the 31-40 years group (21.4%). Urban residents had a higher rifampicin resistance rate (18.6%) than rural residents (7.5%). The rifampicin resistance rate was relatively consistent across study sites: Gondar (14%), Bahir Dar (12%), and Debre Markos (12%). A study in Pakistan among pediatric PTB patients found 4.5% rifampicin-resistant *M. tuberculosis*, a rate lower than the 12.7% reported here, reflecting possible differences in population and settings [25]. A study in Southwest Ethiopia found rifampicin resistance at 3.4%, which is lower than the current study, but was noted to be higher in previously treated cases, aligning with the increased resistance seen in certain age and urban groups in this data [26]. Another Ethiopian study reported rifampicin resistance at 9.8%, close to but slightly lower than the 12.7% seen, with similar observations of higher resistance among females and HIV status [27]. A study reviewing a larger population showed a

declining trend, but still reported notable resistance levels in both adults and children, with 8.3% rifampicin resistance among adults and 7.2% in children, somewhat lower but comparable to the current findings [28].

Socio-economic determinants such as low education and poor knowledge contribute to delayed diagnosis and poor adherence, exacerbating rifampicin resistance risks, particularly in urban and certain occupational groups with higher social mixing. The influence of income and household size on resistance is less frequently documented explicitly but is consistent with observations of complex risk factors influencing PTB epidemiology and drug resistance [15, 29]. Interventions targeting education, treatment adherence, and socioeconomic barriers remain crucial in controlling rifampicin-resistant *M. tuberculosis* [27, 30]. A meta-analyses and pooled molecular-surveillance studies for Ethiopia report pooled rifampicin resistance (RR) generally in the 7-9% range, so our study 12.7% is noticeably above those pooled averages, suggesting either local hot-spots or sample differences [6, 31].

Rifampicin resistance was highest among those with Grade 1-4 education (20.0%), followed by grade 5-8 (16.7%) and grade 9-12 (14.8%). This aligns with broader patterns showing elevated RR or MDR-TB among populations with low educational attainment, though explicit stratification by educational level is not frequently reported. For instance, one study from Northwest Ethiopia found that poorest and least educated adults had disproportionately higher MDR-TB rates, underscoring the vulnerability of the poorly educated to drug-resistant disease [32]. Data on occupation, merchants (21.9%) and government employees (15.2%) showed with notably higher resistance compared to farmers (6.6%), and none among students. In contrast, a cross-sectional analysis from West Armachiho and Metema districts in Northwest Ethiopia reported no significant association between occupation and MDR-TB after adjustment; although raw numbers showed some MDR-TB among farmers/day laborers, the regression found occupation-including being a farmer-was not a predictor once other factors were accounted [33]. In the current study, a relatively high proportion of indeterminate results (7.3%) in *M. tuberculosis* rifampicin susceptibility testing, particularly when using the GeneXpert MTB/RIF assay, may be attributed to several well-recognized technical and biological factors. These include low bacillary load in clinical specimens, suboptimal sample quality, the presence of PCR inhibitors, instrument- or cartridge-related issues, and mutations occurring outside the rifampicin resistance–determining region (RRDR).

In this study, the prevalence of PTB among suspected cases was influenced by several socio-demographic characteristics. Male participants showed a slightly higher PTB prevalence (6.4%) compared to females (5.4%), though the association was not statistically significant, aligning with studies from Ethiopia,

Uganda, and India that consistently reported higher PTB infection among men, likely due to greater occupational exposure and health-seeking behavior differences [34-36]. In the multivariate model, age, residence and occupation were independently associated with PTB status. Older age categories showed significantly lower odds of PTB compared with the 8-15-year age group (AOR: 0.06-0.14; p= 0.001). In contrast, a study reported that older age showed higher odds for PTB, highlighting a higher PTB risk in adults than children and adolescents in sub-Saharan Africa [37]. Further, these findings were inconsistent from other studies in Ethiopia and low- and middle-income countries (LMICs), where low education is frequently associated with higher PTB risk due to its correlation with socioeconomic status and health literacy [38, 39]. Students by occupation remained lower odds for PTB (AOR: 0.03; 95%CI: 0.01-0.11) compared to government employees. This is contrary to study in Ethiopia which showed type of occupation was associated with increased odds of active PTB disease. Additionally, broader literature on PTB and socioeconomic status demonstrates that low socioeconomic status is consistently associated with higher PTB incidence and vulnerability [4, 40].

Chest pain was a less likely indicators of PTB, with adjusted odds ratio (AOR: 0.25; 95%CI: 0.05-0.87; p= 0.031), indicating that PTB suspects presenting chest pain are less likely to be PTB-positive. However, cough and fever are common in PTB, were not strongly associated in this analysis, echoing the diagnostic challenges reported in Ethiopian health facilities. Weight loss and hemoptysis did not reach statistical significance in multivariate analysis, which matches observations that such symptoms are less sensitive for PTB diagnosis in generalized screening but may still indicate advanced disease [41].

Previous antibiotic use and alcohol drinking were significantly associated with PTB, whereas HIV status, diabetes, smoking, family history of TB, distance to health facilities, and first health-seeking actions were not significant after adjustment. This finding is consistent with a study from Debre Markos Referral Hospital, which reported a significant association between previous antibiotic use and PTB [42]. Similarly, studies from Sekota town identified alcohol consumption as a significant factor, although they also reported HIV seropositivity and contact with PTB patients as important predictors, unlike the present study [39, 43]. In contrast, studies from Adigrat General Hospital demonstrated that HIV infection and age were significantly associated with rifampicin resistance, differing from the current non-significant association with HIV [44, 45]. Although cigarette smoking was significant in the bivariate analysis, it lost significance in the multivariate model, despite strong evidence from other studies showing smoking as an independent risk factor for PTB progression and poor outcomes [46]. Alcohol drinking remained significant in both models, supporting previous Ethiopian and international evidence linking alcohol use with increased PTB

susceptibility and adverse outcomes [47]. Furthermore, source of drinking water and distance to health facilities were not significant, diverging from studies that report these factors as important for other infectious diseases but less consistently for PTB unless combined with broader social determinants [41, 45, 48].

Differences in prevalence across studies usually reflect: (i) case mix and inclusion criteria (symptom duration thresholds, inclusion of extrapulmonary specimens, proportion of retreatment cases-retreatment consistently shows higher positivity); (ii) diagnostic approach and quality (GeneXpert vs. smear/culture, cartridge performance, error/invalid rates, and laboratory quality systems); (iii) epidemiologic context (local TB/HIV burden, urban-rural composition, crowding, and contact patterns); and (iv) time period/program effects (expansion of screening, earlier care-seeking, and secular declines). For example, retreatment patients had substantially higher positivity than new cases in national program data, while program reports also document cartridge wastage/error that can influence measurable yield; both factors can push site-level prevalence up or down [14, 49]. In short, the lower estimate likely reflects a combination of broader catchment with many low-risk presumptive, fewer retreatment cases, and improved case-finding relative to earlier single-site studies.

Limitations of the study

The use of the GeneXpert MTB/RIF assay, detects only rifampicin-resistant and not resistance to other first-line or second-line anti-TB drugs. The study included patients from selected comprehensive specialized hospitals, which may not represent the broader population in the Amhara National Regional State, particularly rural areas or those without access to advanced health facilities. Information on socio-demographic and clinical factors was collected using self-reported questionnaires, which could be subject to recall bias and social desirability bias.

5. Conclusion and recommendation

The prevalence of *M. tuberculosis* among suspected cases was 5.9%, with higher rates observed among older age groups and urban residents. Rifampicin resistance was observed in 12.7% of isolates. Pulmonary tuberculosis was significantly associated with prior antibiotic use and alcohol consumption. Strengthening early *M. tuberculosis* detection and routine rifampicin resistance testing is crucial, especially among high-risk groups. Interventions should promote appropriate antibiotic use, and reduce alcohol consumption. Focused health education and ongoing surveillance of drug-resistant *M. tuberculosis* are needed to limit transmission and improve PTB control in the region.

Abbreviations

AMR: Antimicrobial resistance, DMCSH: Debre Markos Comprehensive Specialized Hospital, FHCSH: Felege-Hiwot Comprehensive Specialized Hospital, MDR-TB: Multidrug-resistant tuberculosis, PTB: Pulmonary tuberculosis, UoGCSH: University of Gondar Comprehensive Specialized Hospital

Acknowledgments

We would like to sincerely thank Debre Markos, Felege-Hiwot and University of Gondar Comprehensive Specialized Hospitals administration and staffs for allowing us to conduct the research in the hospitals. We acknowledge the study participants for their participation without them the research would not be a reality.

Availability of data and material

All relevant data are within the manuscript and its supporting information files.

Competing interest

The authors declare that they have no competing interests.

Funding

The authors received no specific funding for this work.

Authors' contribution

TD: Conceptualization, Methodology, Investigation, Data curation, Project administration, Formal analysis, Resources, Software, Writing- original draft, and Writing- review and editing

FM: Conceptualization, Methodology, Supervision, Project administration, Validation, and Writing- review and editing

BT: Methodology, Supervision, Project administration, Validation, and Writing- review and editing

WH: Methodology, Supervision, Project administration, Validation and Writing- review and editing

BG: Conceptualization, Methodology, Investigation, Supervision, Project administration, Validation, and Writing- review and editing

References

1. WHO. Global tuberculosis report 2023. Geneva: World Health Organization; 2023. Licence: CC BY-NC-SA 3.0 IGO.

2. Salari N, Kanjoori AH, Hosseinian-Far A, Hasheminezhad R, Mansouri K, Mohammadi M. Global prevalence of drug-resistant tuberculosis: a systematic review and meta-analysis. *Infect Dis Poverty*. 2023;12(1):57.

3. FMoH. Ethiopia national TB program annual performance report. Ministry of Health, Ethiopia. 2022.

4. Shimeles E, Enquselassie F, Aseffa A, Tilahun M, Mekonen A, Wondimagegn G, *et al*. Risk factors for tuberculosis: A case-control study in Addis Ababa, Ethiopia. *PloS One*. 2019;14(4):e0214235.

5. Shiferaw MB, Sinishaw MA, Amare D, Alem G, Asefa D, Klinkenberg E. Prevalence of active tuberculosis disease among healthcare workers and support staff in healthcare settings of the Amhara region, Ethiopia. *PloS One*. 2021;16(6):e0253177.

6. Birhanu MY, Bekele GM, Jemberie SS. Molecular detection of rifampicin-resistant *Mycobacterium tuberculosis* by polymerase chain reaction in Ethiopia: a systematic review and meta-analysis. *Front Med*. 2024;11:1319845.

7. WHO. Automated Real-Time Nucleic Acid Amplification Technology for Rapid and Simultaneous Detection of Tuberculosis and Rifampicin Resistance: Xpert MTB/RIF Assay for the Diagnosis of Pulmonary and Extrapulmonary TB in Adults and Children, 2013. Geneva: Copyright © World Health Organization 2013.

8. Boehme CC, Nabeta P, Hillemann D, Nicol MP, Shenai S, Krapp F, *et al*. Rapid molecular detection of tuberculosis and rifampin resistance. *N Engl J Med*. 2010;363(11):1005-15.

9. Badgeba A, Shimbre MS, Gebremichael MA, Bogale B, Berhanu M, Abdulkadir H. Determinants of Multidrug-Resistant *Mycobacterium tuberculosis* Infection: A Multicenter Study from Southern Ethiopia. *Infect Drug Resist*. 2022;15:3523-35.

10. Asmare Z, Assefa A, Belay A, Mulie A, Aschil D, Gashaw Y. Prevalence and factors associated with TB among presumptive TB patients in war-affected areas of North Wollo, Ethiopia. *JAC Antimicrob Resist*. 2025;7(1):dlae206.

11. Girum T, Muktar E, Lentiro K, Wondiye H, Shewangizaw M. Epidemiology of multidrug-resistant tuberculosis (MDR-TB) in Ethiopia: a systematic review and meta-analysis of the prevalence, determinants and treatment outcome. *Trop Dis Travel Med Vaccines*. 2018;4(1):5.

12. WHO. Definitions and reporting framework for tuberculosis-2013 revision. Geneva: WHO. 2013.

13. Ramaswamy S, Musser JM. Molecular genetic basis of antimicrobial agent resistance in *Mycobacterium tuberculosis*: 1998 update. *Tubercle Lung Dis*. 1998;79(1):3-29.

14. Biset S, Teferi M, Alamirew H, Birhanu B, Dessie A, Aschale A, *et al*. Trends of *Mycobacterium tuberculosis* and Rifampicin resistance in Northwest Ethiopia: Xpert® MTB/RIF assay results from 2015 to 2021. *BMC Infect Dis*. 2024;24(1):238.

15. Gebremariam G, Kiros M, Hagos S, Hadush H, Gebremichael A, Gebrekirstos G, *et al*. Trend of pulmonary tuberculosis and rifampicin-resistance among tuberculosis presumptive patients in Central Tigray, Ethiopia; 2018 -2023: a six-year retrospective study. *Trop Dis Travel Med Vaccines*. 2024;10(1):15.

16. Wubu B, Jemal M, Million Y, Gizachew M. Pulmonary tuberculosis and multidrug-resistant *Mycobacterium tuberculosis* in northwestern Ethiopia: a hospital-based cross-sectional study among presumptive pulmonary tuberculosis patients. *Front Med*. 2023;10:1266780.

17. Andarge DB, Anticho TL, Jara GM, Ali MM. Prevalence of *Mycobacterium tuberculosis* infection and rifampicin resistance among presumptive tuberculosis cases visiting tuberculosis clinic of Adare General Hospital, Southern Ethiopia. *SAGE Open Med*. 2021;9:20503121211045541.

18. Banti AB, Winje BA, Hinderaker SG, Heldal E, Abebe M, Dangisso MH, *et al*. Prevalence and incidence of symptomatic pulmonary tuberculosis based on repeated population screening in a district in Ethiopia: a prospective cohort study. *BMJ Open*. 2023;13(7):e070594.

19. Kebede AH, Alebachew Z, Tsegaye F, Lemma E, Abebe A, Agonafir M, *et al*. The first population-based national tuberculosis prevalence survey in Ethiopia, 2010-2011. *Int J Tuberc Lung Dis*. 2014;18(6):635-9.

20. Getahun M, Beyene D, Mollalign H, Diriba G, Tesfaye E, Yenew B, *et al*. Population structure and spatial distribution of *Mycobacterium tuberculosis* in Ethiopia. *Sci Rep*. 2024;14(1):10455.

21. Mbuya AW, Mboya IB, Semvua HH, Mamuya SH, Msuya SE. Prevalence and factors associated with tuberculosis among the mining communities in Mererani, Tanzania. *PloS One*. 2023;18(3):e0280396.

22. Gashaw M, Abtew D, Addis Z. Prevalence and Antimicrobial Susceptibility Pattern of Bacteria Isolated from Mobile Phones of Health Care Professionals Working in Gondar Town Health Centers. *Int Sch Res Notices*. 2014;2014(1):205074.

23. Heaton B. Prevalence of Tuberculosis in Sub-Saharan Africa. Ballard Brief. March 2022.

24. Tola HH, Karimi M, Yekaninejad MS. Effects of sociodemographic characteristics and patients' health beliefs on tuberculosis treatment adherence in Ethiopia: a structural equation modelling approach. *Infect Dis Poverty*. 2017;6(1):167.

25. Jameel R, Mehdi N, Majeed N, Zafar A, Tahir A, Aroob I. Prevalence and Diagnosis of Rifampicin-Resistant Mycobacterium Tuberculosis using the GeneXpert MTB/RIF Assay at a Tertiary Care Children's Hospital: Prevalence and Diagnosis of Rifampicin-Resistant *Mycobacterium tuberculosis. PJHS*. 2024;5(07):110-4.

26. Admassu W, Ayelign B, Abebe G, Tadesse M. Detection of *Mycobacterium tuberculosis* and rifampicin resistance by Xpert® MTB/RIF assay among presumptive tuberculosis cases at Jimma University Medical Center, Southwest Ethiopia. *PloS One*. 2022;17(1):e0262929.

27. Araya S, Negesso AE, Tamir Z. Rifampicin-Resistant *Mycobacterium tuberculosis* Among Patients with Presumptive Tuberculosis in Addis Ababa, Ethiopia. *Infect Drug Resist*. 2020;13:3451-9.

28. Wasihun AG, Hailu GG, Dejene TA. Prevalence of *Mycobacterium tuberculosis* (Rifampicin-Resistant MTB) and Associated Risk Actors Among Pulmonary Presumptive TB Patients in Eastern Amhara, Ethiopia: 2015-2019. *Infect Dis Ther*. 2021;10(3):1299-308.

29. Cadmus S, Akinseye VO, Olagunju T, Makolo A, Falodun O, Lawal O, *et al*. Risk factors for rifampicin resistance tuberculosis among patients attending Directly Observed Treatments Centres in Southwestern Nigeria. *Pan Afr Med J*. 2024;49:87.

30. Arega B, Menbere F, Getachew Y. Prevalence of rifampicin resistant *Mycobacterium tuberculosis* among presumptive tuberculosis patients in selected governmental hospitals in Addis Ababa, Ethiopia. *BMC Infect Dis*. 2019;19(1):307.

31. Demelash M, Nibret E, Hailegebriel T, Minichil Z, Mekonnen D. Prevalence of rifampicin resistant pulmonary tuberculosis using geneXpert assay in Ethiopia, a systematic review and meta-analysis. *Heliyon*. 2023;9(9):e19554.

32. Zereabruk K, Kahsay T, Teklemichael H, Aberhe W, Hailay A, Mebrahtom G, *et al.* Determinants of multidrug-resistant tuberculosis among adults undergoing treatment for tuberculosis in Tigray Region, Ethiopia: a case-control study. *BMJ Open Respir Res*. 2024;11(1).

33. Mekonnen F, Tessema B, Moges F, Gelaw A, Eshetie S, Kumera G. Multidrug resistant tuberculosis: prevalence and risk factors in districts of metema and west armachiho, Northwest Ethiopia. *BMC Infect Dis*. 2015;15:461.

34. Ramos JM, Comeche B, Tesfamariam A, Reyes F, Tiziano G, Balcha S, *et al*. Sex differences and HIV status of tuberculosis in adults at a rural hospital in southern Ethiopia: an 18-year retrospective cross-sectional study. *Afr Health Sci*. 2020;20(2):605-14.

35. Sekandi JN, Quach T, Olum R, Nakkonde D, Farist L, Obiekwe R, *et al.* Stigma and associated sex disparities among patients with tuberculosis in Uganda: a cross-sectional study. *Ther Adv Infect Dis*. 2024;11:20499361241305517.

36. Roy M. Sociodemographic Determinants of Tuberculosis Burden in India. *Amrita J Med*. 2021;17:45.

37. Horton KC, MacPherson P, Houben RM, White RG, Corbett EL. Sex Differences in Tuberculosis Burden and Notifications in Low- and Middle-Income Countries: A Systematic Review and Meta-analysis. *PLoS Med*. 2016;13(9):e1002119.

38. Mohammed H, Oljira L, Roba KT, Ngadaya E, Manyazewal T, Ajeme T, *et al*. Tuberculosis Prevalence and Predictors Among Health Care-Seeking People Screened for Cough of Any Duration in Ethiopia: A Multicenter Cross-Sectional Study. *Front Public Health*. 2022;Volume 9 - 2021.

39. Mengistu G, Tekeste Z, Mehabie D, Tesfaye S, Alelign A. Prevalence, risk factors, and rifampicin resistance pattern of *Mycobacterium tuberculosis* in Sekota town, Northwest Ethiopia. *Parasite Epidemiol Control*. 2026;32:e00471.

40. Wang Q, Guo L, Wang J, Zhang L, Zhu W, Yuan Y, *et al*. Spatial distribution of tuberculosis and its socioeconomic influencing factors in mainland China 2013-2016. *Trop Med Int Health*. 2019;24(9):1104-13.

41. Mohammed H, Oljira L, Teji Roba K, Ngadaya E, Mehari R, Manyazewal T, *et al*. Who to Involve and Where to Start Integrating Tuberculosis Screening into Routine Healthcare Services: Positive Cough of Any Duration as the First Step for Screening Tuberculosis in Ethiopia. *Risk Manag Healthc Policy*. 2021;14:4749-56.

42. Mulu W, Abera B, Yimer M, Hailu T, Ayele H, Abate D. Rifampicin-resistance pattern of *Mycobacterium tuberculosis* and associated factors among presumptive tuberculosis patients referred to Debre Markos Referral Hospital, Ethiopia: a cross-sectional study. *BMC Res Notes*. 2017;10(1):8.

43. Endalew G, Beyene MB, Kassie A, Wassie GT. Incidence and Risk Factors of Tuberculosis among Children Receiving Antiretroviral Therapy in Northwest, Ethiopia. *Int J Public Health*. 2025;70:1607892.

44. Abay GK, Abraha BH. Trends of *Mycobacterium tuberculosis* and rifampicin resistance in Adigrat General Hospital, Eastern zone of Tigrai, North Ethiopia. *Trop Dis Travel Med Vaccines*. 2020;6:14.

45. Diriba K, Awulachew E. Associated risk factor of tuberculosis infection among adult patients in Gedeo Zone, Southern Ethiopia. *SAGE Open Med*. 2022;10:20503121221086725.

46. Alemu YM, Awoke W, Wilder-Smith A. Determinants for tuberculosis in HIV-infected adults in Northwest Ethiopia: a multicentre case-control study. *BMJ Open*. 2016;6(4):e009058.

47. Fenta A, Demeke G, Bitew A, Kebede D, Hailu T. Prevalence and Associated Factors of TB Co-Morbidity Among HIV Sero-Positive Individuals in Shegaw Motta District Hospital, Ethiopia. *Int J Gen Med*. 2020;13:1529-36.

48. Abaynew Y, Ali A, Taye G. Social determinants of tuberculosis in Addis Ababa, Ethiopia: a qualitative study. *Sci Rep*. 2025;15(1):15961.

49. Ramachandra V, Brammacharry U, Muralidhar A, Muthukumar A, Mani R, Muthaiah M, *et al*. Assess the Diagnostic Accuracy of GeneXpert to Detect *Mycobacterium tuberculosis* and Rifampicin-Resistant Tuberculosis among Presumptive Tuberculosis and Presumptive Drug Resistant Tuberculosis Patients. *Microbiol Res*. 2024;15(1):91-108.

Supporting Information

S 1 data: Raw data set in excel

Click here to access/download
Supporting Information
S1 Data.xlsx
