# Supplementary material for: Rifampicin resistance and associated factors of Mycobacterium tuberculosis among pulmonary tuberculosis-suspected patients in the Amhara National Regional State Comprehensive Specialized Hospitals, Ethiopia
Source: PLoS One. 2026 Jun 26;21(6):e0351964. doi: 10.1371/journal.pone.0351964 (PMC13309019; doi:10.1371/journal.pone.0351964)
Supplement: Attachment — Submitted filename: Response to Reviewers.docx [file pone.0351964.s009.docx]

**Response to reviewers**

**Dear Editor- in-Chief,**

**PLoS One**

This is a revised versions of the manuscript entitled: “**Rifampicin resistance and associated factors of *Mycobacterium tuberculosis* among pulmonary tuberculosis-suspected patients in** **the Amhara National Regional State Comprehensive Specialized Hospitals, Ethiopia**” to be considered for publication in “**PLoS One**”.

**Dear Academic Editor,**

We sincerely thank you for your decision and for the valuable detailed comments provided, which have been extremely helpful in improving our manuscript. We are also very grateful to the reviewers for their detailed, thoughtful, constructive comments and questions. Please find the attached revised version of the manuscript, which has been carefully updated in accordance with the comments and suggestions from the editor and reviewers. All comments from the tracked and other attached files have been carefully considered and incorporated into the manuscript. Where appropriate, we have also included brief explanations addressing the concerns raised by the reviewers. In addition, the entire manuscript has been thoroughly proofread to improve clarity, grammar, and overall presentation. We believe that these revisions have significantly strengthened the quality of the manuscript, and we hope that the revised version is now suitable for publication in your esteemed journal.

**Responses to Academic Editor**

1. **Editor**: Attach the reviewer and editor comments as comments in a separate document, and use tracked changes in the main document. Carefully consider each comment, and either correct or justify accordingly in your revision

**Author response**: We are grateful to you for your decision and to the reviewers for the time and effort invested in thoroughly evaluating our work and for providing valuable and constructive comments. We have carefully reviewed all the comments and suggestions raised by the reviewers and have revised the manuscript accordingly. Every concern has been addressed, and the recommended changes have been fully incorporated into the revised version of the manuscript. A detailed, point-by-point response to your comments and each reviewer’s comments is provided below.

2. **Editor**: Please indicate here whether you use secondary data or not

**Author response**: This study utilized primary data collected prospectively, as clearly described in the manuscript. Now additional clarification was included both the abstract and main sections of methods part.

3. **Editor**: Specifically mention, what is the outcome, there is confusing, either PTB or RR-MTB

**Author response**: Thank you for this important comment. In this study, both pulmonary tuberculosis (PTB) and RR-MTB were considered as outcome variables. PTB was defined as the primary outcome (presence of *M. tuberculosis* among suspected patients), while RR-MTB was treated as a secondary outcome among confirmed TB cases. In the revised manuscript, this distinction has been clearly stated in the methods and results sections to avoid any confusion. Associated factor analysis was also performed for RR-MTB.

4. **Editor**: It is less or equal

**Author response**: Thank you for the clarification: Yes, it is less or equal to 0.05 based on the statistical association principle.

5. **Editor**: It is multivariable not multivariate

**Author response**. We accepted the comment and uniformly corrected, as it was ‘bivariable and multivariable’.

6. **Editor**: Be consistent, make it patient

**Author response**: We made consistent by using patient rather than participant throughout the document.

7. **Editor:** Please remove this comparative word and just you can discuss in the discussion part

**Author response**: Thank you for the comment. We removed comparative words from the result part and comparison was performed in the discussion part.

8. **Editor**: In your objective, you plan to determine the risk factors for RIF but here you determine risk factors for PTB, please make it clear?

**Author response:** Thank you for the important comment. Both PTB and RR-MTB were considered in the analysis. Risk factors for PTB were assessed among all suspected patients, while risk factors for RR-MTB were evaluated among confirmed TB cases. This distinction has now been clarified, and the objective and corresponding sections have been revised to clearly indicate that factors associated with both PTB and RR-MTB were investigated in the manuscript (Tables 3, 4 &5).

9. **Editor**: Unnecessary associated factors variables was not important in the abstract

**Author response:** We removed those factors which lacked association with PTB and or RR-MTB and primary focus was given for those variables which have significant associations.

10. **Editor**: You can Make it “Rural residence (AOR: 0.23; 95% CI: 0.06-0.79) and being a student (AOR: 0.03; 95% CI: 0.01-0.11) were associated with significantly lower odds of pulmonary tuberculosis (PTB) infection. In contrast, previous antibiotic use (AOR: 9.17; 95% CI: 2.01-28.14) and alcohol consumption (AOR: 7.10; 95% CI: 4.99-20.86) were independently associated with increased *M. tuberculosis* infection.” If your plan was to determine factor for PTB

**Author response:** Thank you for the suggestion. We accepted the recommendation and revision was performed in the abstract.

11. **Edito**r: You mention several studies but your reference is single i.e [9]

**Author response**: Thank you for this observation. We acknowledge that the initial version cited only a single reference despite referring to multiple studies. In the revised manuscript, we have incorporated additional up-to-date and relevant peer-reviewed references to appropriately support each statement and ensure consistency between the cited evidence and the discussion.

12. **Editor**: Please describe shortly their finding, their limitation and your current study value

**Author response:**

13. **Editor**: Cross check references, showing the regional variation of PTB or RR-TB across the region

**Author response**: We checked the references that described the regional variations of PTB in Ethiopia.

14. **Editor**: The introduction last sentence was different from your objective in your abstract part, there, the risk factor is for RR-PTB

**Author response:** Thank you for this important observation. In the revised manuscript, we have aligned the final sentence of the introduction with the objective stated in the abstract. Specifically, we clarified that the study aims to assess the associated factors for both PTB and RR-MTB, rather than limiting the focus only to RR-PTB. This revision ensures consistency between the introduction and abstract and more accurately reflects the scope of our analysis.

15. **Editor:** Here add, how many specialized hospitals found in the region, how many % of them included in this study? Are these hospitals, treat MDR-TB patient?

**Author response**: There are 8 Comprehensive Specialized Hospitals namely; University of Gondar, Felege Hiwot, Tibebe Ghion, Debre Markos, Debre Birhan, Dessie, Debre Tabor, and Woldia. All of these hospitals provide diagnoses of TB and rifampicin resistance, and standard TB treatment but four comprehensive specialized hospitals (UoG, FH, DM and Woldiya) are dedicated for treatment of MDR-TB. Three hospitals were included in the current study, representing 3 out of 8 (37.5%) of the comprehensive specialized hospitals in the region, and were considered sufficient to represent the entire region based on statistical sampling principles.

16. **Editor**: Why you excluded patients less than 8 years?

**Author response**: Thank you for this observation. Children under 8 years are often excluded primarily due to inability to produce sputum, low diagnostic yield, and difficulty in confirming bacteriological outcomes, which are critical for studies assessing TB and drug resistance patterns.

17. **Editor**: PTB suspected: take these to operational definition and add reference

**Author response**: We took to the operational definitions and operationalized based on the comment.

18. **Editor**: Distance from home to the health facility: Have you collected this?

**Author response**: Yes, we collected and categorized as less or equal to 40 and greater than 40 km based on the face-to-face interview with participant.

19. **Editor**: Your study population was PTB, so, EXPTB is not under your population, so, should not include in exclusion criteria

**Author response**: Thank you for this important comment. We agree that since the study population was limited to PTB-suspected patients, extrapulmonary tuberculosis (EPTB) cases fall outside the defined population and should not be listed under the exclusion criteria. Accordingly, EPTB has been removed from the manuscript part of exclusion criteria.

20. **Editor**: 1. Did you use a sample size calculation formula, or did you simply include all patients who had GenXert for PTB in the selected hospital (which is better)? Why did you choose an equal sample size (50 PTB patients) when it should be based on proportion? 2. Clearly describe the data type (primary vs. secondary), data source, etc. 3. How did you select patients- randomly, systematically, or conveniently?

**Author response**: Thank you for these constructive comments. We have revised the manuscript accordingly and provide the following clarifications:

**1. Sample size determination and allocation across sites**: No formal sample size calculation was performed for this study. Instead, we included all eligible PTB- suspected patients who underwent GeneXpert testing during the study period using a consecutive approach. The decision to enroll an equal number of 50 *M. tuberculosis*-positive cases per site was intentional to ensure balanced representation and enable direct comparison across the three study areas. This approach was adopted in the context of a dissertation project, where equal group sizes also facilitate comparison with parallel study groups (e.g., comparison of PTB-patients versus apparently healthy individuals in terms of diversity and abundance of microbiota, antimicrobial resistance of microbiota, carbapenamase production encoding genes, and treatment outcomes of PTB-patients), although that objective is not part of the present manuscript. Additionally, having comparable sample sizes per site allows site-specific prevalence estimates to be calculated more consistently for microbiota, treatment outcome etc. for the next parts of dissertation.

**2. Data type and data source**: The study utilized primary data collected prospectively from PTB-suspected patients at the selected hospitals. Laboratory results, including GeneXpert outcomes, were obtained directly from routine diagnostic testing, and relevant socio-demographic and clinical information was collected using semi-structured data collection tools.

**3. Sampling technique**: A convenience sampling technique was employed. Specifically, consecutive eligible patients were enrolled at each site until 50 bacteriologically confirmed PTB-cases were obtained per facility. This approach ensured feasibility within the study period while maintaining comparability across sites.

21. **Editor:** Add PTB-suspected patients, RR-MTB, what is indeterminate RR-MTB and what can do for indeterminate as operational definition

**Author response**: We operationalized the PTB-suspected patients, RR-MTB, intermediate RR-MTB. We noted that repeat testing or confirmatory methods (e.g., line probe assay or culture-based drug susceptibility testing) are recommended in routine practice, for this study test reputation with additional sputum sample by GeneXpert was performed, but no change was observed in the result.

22. **Editor:** Socio-demographic data collection: Is mean by interview? or extracted from the secondary data from HMIS or Patient chart?

**Author response**: The data were collected prospectively by trained healthcare professionals. We clarify it by revising as ‘Socio-demographic and clinical data were collected using a semi-structured, pre-tested questionnaire administered through face-to-face interviews by trained healthcare personnel’.

23. **Editor:** These sentences should be rephased, sputum not saliva is the sample, not matter of emphasis

**Author response**: Thank you, we revised it.

24. **Editor:** Bivariable logistic regression analysis was employed to examine the association between independent variables with tuberculosis or rifampicin resistance: for what or both?

**Author response:** Thank you very much for your valuable comment. We performed logistic regression analysis for both associated factors for prevalence of PTB and RR-MTB. A revision was performed to avoid confusion for readers in the methods section.

25. **Editor:** If you determine factor for both/or RR-MTB or PTB. The data interpretation became confusing

**Author response**: Thank you very much for your valuable comment. We prepared separate tables and associated factor analysis were performed for each RR-MTB and PTB and no confusion would be created now (Tables 3, 4 & 5).

26. **Editor:** Have you taken written consent from the 2,548 patients?

**Author response**: Thank you very much for your valuable comment. Yes, we took a written consent from each individual from the three sites 862 from Debre Markos, 904 from Bahir Dar and 782 from University of Gondar Comprehensive Specialized Hospitals. Six individuals participated in the administrations of the consent and interview.

27. **Editor:** Once you abbreviate a word, use the abbreviation thereafter

**Author response**: Thank you, we revised thoroughly the manuscript and corrected.

28. **Editor:** Please use consistent numbering format, number with % or only %, better to use percentage with number proportion. %(a/b) formatting though out you document

**Author response**: Thank you. We revised and used consistent formatting in the use of absolute number and percentages.

29. **Editor:** You can merge the two tables into one compressive table. The 95%CI can be described some main variables like overall PTB prevalence and RR-MTB.

**Author response:** Thank you, we merged table 1 and 2.

30. **Editor:** Have you collected, data regarding the TB type (new vs previous TB treatment history), because the prevalence differed between the two groups

**Author response:** Based on the inclusion criteria, only PTB-suspected newly diagnosed by the authors were includes as study participants. All previous PTB treated, default and lost to follow up were excluded.

31. **Editor:** Is your interest to determine factors for PTB infection or for the RR-MTB, a lot has been done for PTB

**Author response:** Thank you; our objective was to identify the factors associated with both PTB infection and RR-MTB. Separate tables, results in text, and corresponding interpretations were clearly presented for each outcome in the revised manuscript.

32. **Editor**: No need to interpret variable in the univariable regression, just list the variables with p<0.25

**Author response:** Thank you, a modification was performed, not to focus to interpret COR on the univariable logistic regression analysis.

33. **Editor**: If you change the reference value, you can describe the variable that increase, rather than preventing the risk. Complete the interpretation, you miss the income

**Author response:** Thank you for this valuable comment. We agree that the interpretation should be clearer when the reference category is changed. In our analysis, we used the lower-risk or less-affected group as the reference category to improve interpretability of the associations. Accordingly, the direction of effect has been described in relation to this reference group. The monthly income data was included in text.

34. **Editor:** Sex, family size and study area are not candidate in univariable because the p value > 0.25. Not candidate variable because the p value in 0.25

**Author response:** Thank you for the comment. As observed in table 3, the variables including sex, family size and study area were not included in the multivariable logistic regression analysis because since the p-value in the bivariable logistic regression (in COR) was less than 0.25. But the bivariable logistic regression result was included in the table, but not in the multivariable logistic regression column of the table.

35. **Editor:** Please include the result of AOR, result who were candidate in COR

**Author response:** Thank you for this important comment. We would like to clarify that sex was assessed in the bivariable (COR) analysis, and although it was included in the initial evaluation, it did not meet the selection criterion (p < 0.25) to be entered into the multivariable logistic regression model (AOR), as its p-value was 0.316. Therefore, sex was not a candidate variable for the final adjusted analysis, and no AOR result is reported for it in the multivariable model.

36. **Editor:** Study area: What is the meaning or implication of comparing the areas, good to remove?

**Author response:** Thank you for this insightful comment. The comparison of study areas is intentional and important. The inclusion of different study areas allows for meaningful comparisons across settings, which can help identify potential variations in disease burden, resistance patterns, and associated factors between hospitals or catchment populations. Furthermore, these study areas provide a valuable foundation for future research directions within the dissertation framework. They can support comparative analyses such as differences in microbiota profiles, treatment outcomes, and other clinical or laboratory parameters when compared across sites or even against apparently healthy control groups. Such comparisons are essential for understanding regional heterogeneity and for informing targeted interventions and subsequent studies. Therefore, we will retain the study area.

37. **Editor:** Regroup this into meaningful risk like formally employed (government employed), informally employed (merchant, farmer, daily laborer), and not employed (student, others)."

**Author response:** Thank you for this constructive comment. In response, we have recoded the occupational variable into more meaningful and analytically appropriate categories as suggested. Accordingly, employment status was regrouped into three categories: formally employed (government employed), informally employed (including merchants, farmers, and daily laborers), and not employed (including students and others).

38. **Editor:** Family size: This not candidate variable because the p value is > 0.25

**Author response:** Thank you. It was correct that the p value was > 0.25, and not candidate for multivariable logistic regression analysis.

39. **Editor:** You can make divorced and widowed into Ever-married

**Author response:** Thank you, we accepted the comment and considered both variables as one (ever-married) and reanalyzed.

40. **Editor:** Please first construct the frequency table of the variables included above under subtitles, clinical and behavioral characteristic

41. **Author response:** Thank you, we constructed a new table 2, which for clinical and behavioral characteristic of patients.

42. **Editor:** Here, your candidate was not those with significantly significant in COR, rather with p < 0.25, state in terms of p< 0.25, No need to interpreted the COR, see above

**Author response:** Thank you. We have removed unnecessary interpretation of the COR values and have updated the text to clearly reflect the p < 0.25 screening criterion used for variable selection.

43. **Editor**: This is clinical manifestation of patients with PTB, not considered as risk factors for PTB infection. Might be important when the aim is contact tracing. For example, patient with hemoptysis more likely to have PTB. Remove

44. **Author response:** Thank you for this important observation. We agree that clinical manifestations such as hemoptysis are not independent risk factors for PTB infection, but rather diagnostic indicators that are more relevant for case identification and contact tracing. Therefore, following your suggestion, we have removed these variables from the associated factors analysis table. Instead, we have summarized the clinical manifestations using a descriptive frequency table to appropriately reflect their distribution among the study participants.

45. **Editor**: 1. Separate these variable and do individually, HIV/AIDS and do as Yes/NO, Do similarly for DM(Yes/No), Asthma (yes /No), Previous Pneumonia treatment history (Yes/No). 2. If you considering to compare patient having underlying comorbidities (HIV, DM, HTN, Asthma, might be others) Vs has no underlying, UTI and Pneumonia are not underlying comorbidities these can be treated and cured

**Author response:** Thank you for this valuable comment. We have addressed it by separating each comorbid condition and clinical factor (such as HIV status, diabetes, asthma, and others), assigning appropriate codes, and reanalyzing the data accordingly (see Table 4).

46. **Editor**: Is the prevalence of HIV among your population 13.1%? for DM 21.5%, please prepare descriptive table for all these variables

**Author response:** Now, we explicitly presented the clinical and behavioral characteristics of study participants.

47. **Editor:** Previous antibiotics use: When?

**Author response:** Thank you for the valuable comment. We operationalized this phrase as ‘Previous antibiotic use refers to the history of taking any antibiotic medications within 6 months, regardless of whether the drugs were prescribed by a health professional or obtained without prescription’ and incorporated to the manuscript.

48. **Editor**: Family history of PTB: When?

**Author response:** We revised the previous wording (family history of TB) by a clearer concept ‘Presence of PTB patient in the family’. This can be interpreted as any individual presented in the family any time will be considered as ‘Presence of PTB patient in the family’.

49. **Editor**: Types of health facility visited: What dose these mean, referring facilities? are facilities they commonly use?

**Author response:** Based on the comments, we carefully saw its importance being included in the associated factors and we got less important and removed.

50. **Editor**: The discussion is mixed and somewhere confusing. First paragraph summarizes the result. Second paragraph: discussion prevalence of the PTB. Third paragraph: discussion on the RR-PTB. Other: discussion on significantly associated risk factors (from the strongly associated to less associated). Do not write discussion for the dissipative result

**Author response:** Thank you for the detailed comments and guides. We intensively revised the discussion part based on the forwarded comments and recommendations.

51. **Editor**: From all the references studies, your finding nearly halved of the least reported, what do think is the possible explanation, please explain the possible reasons

**Author response:** We appreciate the reviewer’s insightful comment. Although the prevalence of PTB observed in our study was nearly half of the lowest previously reported findings, several plausible explanations may account for this difference. In the revised manuscript, we included detailed explanations for the comparatively lower prevalence observed across different parameters. For example, ‘The lower prevalence of MTB in the present study compared with previous Ethiopian reports may be due to differences in study population, as the current multi-center design may dilute positivity compared to earlier single-center studies focusing on high-risk groups such as defaulters and lost follow-up patients. Variations in geographic TB burden, improved TB control interventions, and expanded GeneXpert access may also have reduced positivity rates. In addition, differences in diagnostic practices, sputum quality, and health-seeking behavior, as well as temporal declines in TB transmission and variation in HIV co-infection rates, may further explain the observed discrepancy.’

52. **Editor**: In the final model of your study, older age had lower risk compare to young

**Author response:** A through revision was performed to make smooth compatibility in the result part and discussion.

53. **Editor**: In your data, compared to >6000-birr, patient with < 3000 had lower risk for PTB, TB is the disease of poverty, put reasonable evidence for such condition from your study perspective

**Author response:** We incorporated the reasons for the PTB more prevalent in those who have better income than lower income in the discussion part.

54. **Editor**: In your data being single decreasing PTB compered to currently married

**Author response:** The lower PTB prevalence among single individuals compared with currently married participants can be explained by single individuals are often younger and may have had shorter cumulative exposure time to MTB compared with married individuals, who are generally older and have longer lifetime exposure. In addition, currently married individuals are more likely to experience close and repeated household contact, which increases the risk of intra-household transmission if a family member is infectious. Conversely, single individuals may have more dispersed living arrangements and fewer prolonged close contacts.

55. **Editor**: Paragraph 3,4,5 of the discussion is repeated conceptually

**Author response:** Thank you for the comment. These paragraphs discussed more about prevalence of MTB with respect to sociodemographic factors such as residence, income, educational and employment status. We revised these paragraphs to make them compatible to the work keeping them their being three.

56. **Editor**: This is descriptive; no discussion is needed. The comparison between sociodemographic variables is not statistically supported.

**Author response:** We accepted the comment and the content was removed.

57. **Editor**: The rifampicin resistance rate was relatively consistent across study sites: Gondar (14%), Bahir Dar (12%), and Debre Markos (12%). Where is the reference?

**Author response**: These are the current results from the multicenter study area as the current study conducted in three sites. Therefore, no reference was needed, but revision was performed to avoid confusion for readers.

58. **Editor**: Line 33: … but was noted to be higher in previously treated cases, aligning with the increased resistance seen in certain age and urban groups in this data. Rephrase

**Author response:** We revised this statement in line with the comments forwarded.

59. **Editor**: To say higher, lower, or equivalent, you can take the 95% confidence interval of your variable of interest (RR-MTB). If the finding of the other study is within the confidence interval, it is similar; if above, it is higher; if below, it is lower.

**Author response:** Thank you very much for your valuable comment. We added the 95%CI and discussions were adjusted based on the values of 95%CI.

60. **Editor**: The discussion should be based on your own findings. In this paragraph, you have not presented any data regarding adherence, knowledge, or the other variables described here.

**Author response:** We appreciate the insightful comment. We agree that the discussion section included references to factors such as treatment adherence, patient knowledge, and related variables that were not directly assessed in our study. In response, we have revised the paragraph to ensure that the discussion is strictly aligned with our findings and only reflects variables that were measured and analyzed in the present study.

61. **Editor**: Numerous studies have identified risk factors for RR-MTB. Therefore, what scientific value is there in discussing variables that have not been statistically tested as risk factors for RR-MTB?

**Author response:** We appreciate the reviewer’s careful reading, which helped us identify and correct this error. We reassessed and stick to those variables tested associated significantly to MTB and or RR-MTB.

62. **Editor**: Remove such number in the discussion (AOR: 0.06-0.14; *p*= 0.001).

**Author response:** We removed the numbers from the discussion unless if obligatory.

63. **Editor:** The finding in your study contrasts with most previous findings. What explains this discrepancy? Have you considered reanalyzing your data (e.g., checking for confounding factors, data entry errors, or different subgroup effects)?

**Author response:** Thank you for such important comment. We reassessed the data and reanalyzed including the recoded variables such as employment status and marital status and we got consistent finding. The reasons for variations were mentioned in each respective section.

64. **Editor**: Remove this, as reasoned in the results section

**Author response:** We accept the recommendation and removed the clinical factors as they were not associated factors rather indicators of PTB disease based on the comment.

65. **Editor:** Comorbidities: See result. Reanalysis and if significant discuss

**Author response**: The comorbidities and other conditions among study participants were recoded and reanalyzed and discussed well based on the analysis result.

66. **Editor:** Cigarette smoking: Discuss only about variable significantly associated in the final model

**Author response**: Thank you for the valuable comment. We agree that cigarette smoking was not significantly associated with PTB in the multivariable logistic regression analysis. Accordingly, we revised the discussion section to focus only on variables that remained statistically significant in the final model and removed the discussion related to non-significant associations, including cigarette smoking, to improve the scientific consistency and clarity of the manuscript.

67. **Editor**: These possible reasons are important, but put them where the specific variable is discussed

**Author response:** Thank you for the constructive comment. We revised the discussion section accordingly by incorporating the possible reasons for variations directly under each respective variable discussion rather than presenting them separately. Specifically, explanations for discrepancies between the current study and previous reports regarding the prevalence of PTB, rifampicin-resistant *M. tuberculosis* (RR-MTB), and associated factors were integrated within their corresponding discussion paragraphs to improve coherence, clarity, and logical flow of the manuscript.

68. **Editor:** …....self-reported questionaries: What does this mean: 'Because in your method, the data acquisition is not clearly mentioned'?

**Author response:** Thank you for the comment. We clarified the data acquisition procedure in the methods section. The questionnaires were interviewer-administered by trained healthcare professionals specifically assigned for data collection. The term “self-reported” was used to indicate that the responses provided during the interview depended on the participants’ willingness and ability to give accurate and honest information regarding their sociodemographic and clinical characteristics. We revised the manuscript to make this point clearer and avoid ambiguity.

69. **Editor**: Conclusion: Previous antibiotics use and alcohol consumption: You identified many other factors. Did you select only two, or did you include others in the conclusion?

**Author response:** Thank you for the constructive comment. The conclusion and recommendations of the manuscript were prepared based on the three main study objectives: the prevalence of PTB, the prevalence of RR-MTB, and factors associated with PTB and rifampicin resistance. Therefore, only variables that showed statistically significant associations in the final multivariable analysis were emphasized in the conclusion section. Previous antibiotic use and alcohol consumption and HIV positivity status (after recoding) were highlighted because they remained significantly associated with PTB, while residence was included in relation to RR-MTB. Following your comment, the conclusion section was further revised to clearly and comprehensively reflect all significant findings corresponding to the study objectives.

**Responses to Reviewer #1**

Thank you dear reviewer for your time, detail, observations and forwarded comments and questions to us for the betterment of our manuscript. We accepted the comments and incorporated to the manuscript and we gave some responses point-by-point to your comments, suggestions and questions.

1. **Reviewer 1:** This study emphasizes the prevalence of tuberculosis among the general population of the Amhara Region, Northwest Ethiopia. The topic is highly interesting and of public health concern. The manuscript was well prepared and clearly articulated. However, very minor modifications are needed.

**Author response:** We thank the reviewer for this important and insightful, and promising comment.

2. **Reviewer** **1**: It would be better to see the prevalence of rifampicin resistance among the overall suspected cases.

**Author response**: Thank you for the constructive suggestion. We understand the reviewer’s interest in seeing the prevalence of rifampicin (RIF) resistance among all PTB-suspected participants. However, rifampicin resistance patterns (resistant or sensitive) can only be determined among participants who tested positive for *M. tuberculosis*. Participants who were negative for MTB do not have interpretable RIF susceptibility results and therefore cannot serve as part of the denominator for calculating RIF resistance or sensitivity patterns. For this reason, the prevalence of RIF resistance was calculated only among confirmed MTB-positive cases, which is consistent with the standard interpretation and reporting of Xpert MTB/RIF assay results.

3. **Reviewer** **1**: In Table 3, the variable stated as "others" under occupation is significantly associated. Don't you think this will be difficult to discuss?

**Author response**: We agreed with the reviewer’s suggestion. In response, we have recoded the occupational variable into more meaningful and analytically appropriate categories as suggested. Accordingly, employment status was regrouped into three categories: formally employed (government employed), informally employed (including merchants, farmers, and daily laborers), and not employed (including students and others). Now, it became scientifically sound for discussion and discussed well.

4. **Reviewer** **1**: The title of Table 4 needs a slight modification. The word "associated factors" is a little vague. Please change to "clinical data." (Line No. 266)

**Author response**: We thank for your valuable observation. The table was revised and clinical, and behavioral data were separately prepared and presented. The title was modified accordingly for (Table 2 & 4).

**Responses to Reviewer #2**

Thank you dear reviewer, for your time, comments and questions to us for improvement of our manuscript. We accepted comments and incorporated to the paper and we gave some responses to your comments and questions below.

1. **Reviewer 2**: The overall comments are attached to the system. The manuscript is technically sound, and the analysis is well done. Moreover, the manuscript is presented in an intelligible fashion and written in standard English, except for some editorial errors, unnecessary details, and some repetitions in the main document. Besides, the manuscript is comprehensive as it has been conducted using a multicenter approach.

**Author response**: We sincerely thank the reviewer for the comprehensive and constructive overall comments. We appreciate the recognition that the manuscript is technically sound, the analysis is well conducted, and the study is comprehensive due to its multicenter approach. Accordingly, we carefully revised the manuscript to improve clarity, conciseness, readability, and consistency in language throughout the document.

2. **Reviewer 2**: Make this objective as part of the background or put at the end, by stating like: Therefore, this study was aimed….

**Author response**: Thank you for the comment important for the improvement of the manuscript. Since PLoS One journal formatting allows authors to select their own form in the abstract that whether the objective was placed in new sentence or not. Therefore, we preferred the objective to be placed in a separate sentence intending which might give emphasis.

3. **Reviewer 2**: Re-estate the result as given in the document, and remove unnecessary details as stated or corrected by the track changes

**Author response**: We agree with the reviewer’s suggestion. We revised and removed unnecessary detail and a modification was performed in the abstract.

4. **Reviewer 2**: Remove the numbers/the percentage details in the conclusion part and the key word “Tuberculosis” in the key word

**Author response**: We appreciate the reviewer’s valuable comment. We accepted the suggestion and removed the numerical/percentage details from the conclusion section to improve clarity and conciseness. In addition, the keyword “Tuberculosis” was removed from the keywords section as recommended.

5. **Reviewer 2**: Consider the use of terms “several studies and putting one reference at the end; need consideration or inclusion of references

**Author response**: We added references which were directly referred to the statement mentioned.

6. **Reviewer 2**: Check some statements without reference

**Author response**: We appreciate the reviewer’s valuable comment. We checked references missed and incorporating based on the statement which was taken.

7. **Reviewer 2**: In the study sites, better to abbreviate once and use in the rest part of the document

**Author response**: We applied the use of abbreviations throughout the manuscript once written in both versions.

8. **Reviewer 2**: On line 146 to 148; it is better to put your references where your questionnaire is adapted.

**Author response**: We cited sources of references from which the questionnaire was prepared.

9. **Reviewer 2**: Line 169 to 170: Remove as it is a reptation from the above sentence (Variables with p-value less than or equal to 0.25 in the bivariate analysis were jointly entered into a multivariate logistic regression analysis)

**Author response**: Thank you for the comment. We removed the repetitions which appeared two times in the data analysis and interpretations section.

10. **Reviewer 2**: Line 172; Better to include a statement like: Finally, the data was presented using texts, figures…

**Author response**: Thank you. We included a statement which can describe the use of descriptive statistics to present, organize, summarize, and interpret the data (Lines 181 & 182).

11. **Reviewer 2**: Line 224; on the result part of the sub title: As you were studied on PTB, it is better to say: Associated factors for PTB infection

**Author response**: We accepted the suggestion and revised based on the comment.

12. **Reviewer 2**: In assessing the variables, of the two tables, you better to indicate those p-values <0.05 with * and give the key at the bottom

**Author response**: Thank you for the valuable suggestion. We accepted the recommendation and revised the associated factor tables accordingly. Variables with p-values ≤ 0.25 in the bivariable analysis were indicated using a single asterisk (*), while variables with p-values ≤ 0.05 in the multivariable analysis were indicated using a double asterisk (**). Appropriate keys explaining these symbols were also added at the bottom of each table for clarity.

13. **Reviewer 2**: “The study included patients from selected comprehensive specialized hospitals, which may not represent the broader population in the Amhara National Regional State, particularly rural areas or those without access to advanced health facilities” may not be the limitations of your study. This is because these hospitals are those referral and specialized hospitals, where the samples are referred from the rural health centers to these hospitals too.

**Author response**: We revised the limitation part of the manuscript based on the comments.

14. **Reviewer 2**: In steady of writing these numbers here in the conclusion part, it better to re-state for what the observed finding/s mean

**Author response**: Thank you for the valuable recommendation regarding the removal of number figures. We agree that minimizing excessive numerical presentation can improve clarity. However, considering the context and the need to address the three specific objectives of the study, we intentionally retained selected figures together with descriptive text to make the findings more explanatory and easier for readers to interpret. We have also revised the presentation to improve readability and maintain a clear flow of information throughout the manuscript.

15. **Reviewer 2**: If you abbreviate e.g. DMCSH, you need to use in the document

**Author response**: Thank you for the important comment. We agree with the concern regarding the inconsistent use of abbreviations and long forms. We carefully revised the manuscript and ensured that the abbreviation was used consistently throughout the manuscript to improve clarity and readability.

16. **Reviewer 2**: The result part: If you have a table at the end, you better state the main or those variables having the highest only, do not put all the details

**Author response**: We accepted and variables of middle value were left in the table than writing in text.

17. **Reviewer 2**: Check the numbers or values in the table 3 and table 4 including the rules you stated in the methods, analysis, part (p-value <0.25 to be fitted to the MVLR analysis)

**Author response**: We reinspect and checked whether variables in tale 3 and 4 were candidates for multivariable logistic regression or not. If any corrections were made.

18. **Reviewer 2**: Your discussion is too much, remove unnecessary details or better to write to the point. You should put your findings, followed by implications, if possible, then compare with the previous findings and give the possible explanations/justifications for the observed variations

**Author response**: Thank you for the comment. We removed unnecessary details and which were not significant in our study to discuss.

19. **Reviewer 2**: To compare your findings with the previous studies, you better show the 95%Cis for your findings including for the drug resistance part.

**Author response**: Thank you for the observation. The 95% CI was included in the main findings to be discussed in the discussion section.

20. **Reviewer 2**: What is your justification for the exclusion of age < 8 years?

**Author response**: Thank you for this observation. Children under 8 years are often excluded primarily due to inability to produce sputum, low diagnostic yield, and difficulty in confirming bacteriological outcomes, which are critical for studies assessing prevalence of PTB and drug resistance patterns.

21. **Reviewer 2**: What is your justification to consider 150 positives why not 100 or 200?

**Author response**: Thank you for these constructive comments. We have revised the manuscript accordingly and provide the following clarifications: No formal sample size calculation was performed for this study. A total of 2,548 PTB-suspected patients were enrolled the three health facilities which was ample to become representative. This approach was adopted in the context of a comparative study for a dissertation project (PTB-patients and apparently healthy individuals) to compare MTB, RR-MTB within each study area and microbiota diversity, abundance, antimicrobial resistance pattern, carbapenamase encoding genes and treatment outcomes of PTB-patients).

22. **Reviewer 2**: Why you operationally define this??? Is it your interest or your objective to assess the resistance of isoniazid or MDR?

**Author response**: We thank the reviewer for raising this important point. We removed this operational definition as we did not use it as a main objective than using the abbreviation somewhere in the manuscript.

**Responses to Reviewer #3**

Thank you, dear reviewer, for your time, the detailed comments and questions to us for improvement of our paper. We accepted comments and incorporated to the paper and we gave some responses to your comments below.

1. **Reviewer 3:** This manuscript presents good work addressing the current global issue of antimicrobial resistance. The topic is timely and relevant, and the study contributes valuable information to the field. Please refer to my detailed comments and suggestions provided in the attached PDF.

**Author response**: We sincerely thank Reviewer for the thorough, insightful, and constructive evaluation of our manuscript and we brought some of the comments from the PDF version and responses were given below.

2. **Reviewer 3:** The term “multivariate logistic regression” is misused; it should be replaced with “multivariable logistic regression” throughout the document.

**Author response**: We revised and bivariable and multivariable logistic regression was used throughout the document.

3. **Reviewer 3:** The comparison should be avoided in the result section

**Author response**: Thank you for the constructive comment. We agree that comparisons with other studies should not be included in the results section. Accordingly, we revised the manuscript by removing comparative statements from the results section and retained only the presentation of the study findings. Comparisons and interpretations were moved to the discussion section where appropriate.

4. **Reviewer 3:** It is better you remove a specific date for data collection

**Author response:** Thank you, we accepted the comment and removed the specific date keeping the months and years.

5. **Reviewer 3:** Please ensure consistent capitalization of “Comprehensive Specialized Hospitals” throughout the manuscript

**Author response:** We applied consistent use of “Comprehensive Specialized Hospitals’ throughout the manuscript.

6. **Reviewer 3:** Once you used both the abbreviations and long forms, then you better use the abbreviation consistently.

**Author response:** Thank you for the important comment. We agree with the concern regarding the inconsistent use of abbreviations and long forms. Accordingly, we carefully revised the manuscript and ensured that, after the first definition, the abbreviation was used consistently throughout the manuscript to improve clarity and readability.

7. **Reviewer 3:** It is better to use “rifampicin resistance pattern” rather than “rifampicin susceptibility pattern,” since resistance is predominantly reported.

**Author response:** We accepted and incorporated to the document.

8. **Reviewer 3:** The authors should clearly describe the sampling technique and provide the method used to determine the sample size of 150 TB patients.

**Author response:** Thank you for these constructive comments. We have revised the manuscript accordingly and provide the following clarifications: No formal sample size calculation was performed for this study. A total of 2,548 PTB-suspected patients were enrolled the three health facilities which was ample to become representative. The study included all eligible PTB- suspected patients who underwent GeneXpert testing during the study period using a consecutive approach. The decision to enroll an equal number of 50 *M. tuberculosis*-positive cases per site was intentional to ensure balanced representation and enable direct comparison across the three study areas. This approach was adopted in the context of a dissertation project (e.g., comparison of PTB-patients versus apparently healthy individuals in terms of diversity and abundance of microbiota, antimicrobial resistance of microbiota, carbapenamase production encoding genes, and treatment outcomes of PTB-patients). A convenience sampling technique was employed.

**Responses to Reviewer #4**

Thank you, dear reviewer, for your time, the detailed, critical comments and questions to us for improvement of our paper. These comments not only important for such specific paper but also for the general project ongoing on respiratory microbiota. We accepted comments and incorporated to the paper and we gave some responses to your comments below.

1. **Reviewer 4:** The manuscript is generally well organized; however, several aspects require revision to enhance its scientific rigor, clarity, and overall quality. Specifically, the methods and results sections need revision.

**Author response**: Thank you for the constructive comments and overall evaluation of our manuscript. We appreciate the reviewer’s suggestions regarding the need to improve the scientific rigor, clarity, and overall quality of the manuscript. In response, we thoroughly revised the methods and results sections to enhance clarity, consistency, and scientific presentation. The methodological descriptions were expanded and clarified, while the results section was reorganized and refined to improve readability and accurate interpretation of the findings.

2. **Reviewer 4:** Please include brief background information on rifampicin resistance to better contextualize the study.

**Author response**: Thank you for the constructive comment. We accepted the suggestion and included brief background information on rifampicin resistance in the introduction section to better contextualize the study and highlight its public health importance (Lines 34 & 35).

3. **Reviewer 4:** It is preferable to replace the phrase “to determine” with “to assess” for improved academic tone.

**Author response**: We accepted and the word ‘determine’ was replaced by ‘assess’ (Line 38).

4. **Reviewer 4:** The conclusion requires revision to ensure consistency with the stated objective and results.

**Author response**: We revised the conclusion part of the abstract based on the study objectives.

5. **Reviewer 4:** Statements such as “the prevalence remains significant” should be supported by a clear baseline or comparison (e.g., previous studies in the study area or national estimates). Consider using terms like high or low with appropriate references.

**Author response**: Thank you for this important comment. We revised the manuscript accordingly to make more precise interpretations supported by relevant literature and comparative evidence.

6. **Reviewer 4:** Key statistically significant factors identified in the results (e.g., age, residence, occupation, income, marital status) should also be reflected in the conclusion and recommendations.

**Author response**:

7. **Reviewer 4:** Provide justification for excluding patients aged <8 years and clarify the rationale for including participants aged ≥8 years.

**Author response**: Thank you. Children under 8 years of age are less likely to produce adequate sputum samples, which are essential for GeneXpert MTB/RIF and culture-based confirmation of pulmonary tuberculosis. In this age group, TB diagnosis is often made using alternative approaches such as gastric aspirates, clinical scoring systems, and radiological assessment, which were not part of the current study protocol. In addition, younger children often present with paucibacillary disease, reducing the sensitivity of bacteriological tests used in this study.

8. **Reviewer 4:** The sample size calculation is not clearly described. Please specify how the total sample size and the 150 *M. tuberculosis* cases were obtained. A clear sample size calculation formula and assumptions and proportional allocation of participants across study sites

**Author response**: Thank you for these constructive comments. We have revised the manuscript accordingly and provide the following clarifications: No formal sample size calculation was performed for this study. A total of 2,548 PTB-suspected patients were enrolled the three health facilities which was ample to become representative. The study included all eligible PTB- suspected patients who underwent GeneXpert testing during the study period using a consecutive approach. The decision to enroll an equal number of 50 *M. tuberculosis*-positive cases per site was intentional to ensure balanced representation and enable direct comparison across the three study areas. This approach was adopted in the context of a dissertation project (e.g., apparently healthy individuals in comparison with PTB-patients, microbiota, treatment outcomes of PTB-patients). The study utilized primary data collected prospectively from PTB-suspected patients at the selected hospitals. Laboratory results, including GeneXpert outcomes, were obtained directly from routine diagnostic testing, and relevant socio-demographic and clinical information was collected using semi-structured data collection tools. A convenience sampling technique was employed.

9. **Reviewer 4:** The statement “50 *M. tuberculosis*-positive participants were included from each health facility” needs reconsideration. A proportional allocation based on the source population of each facility would be more appropriate than equal allocation.

**Author response**: The decision to enroll an equal number of 50 *M. tuberculosis*-positive cases per site was intentional to ensure balanced representation and enable direct comparison across the three study areas. This approach was adopted in the context of a dissertation project to accommodate further other objectives which are not part of this manuscript (e.g., comparison of PTB-patients versus apparently healthy individuals in terms of microbiota, antimicrobial resistance pattern of microbiota and carbapenamase encoding genes, and treatment outcomes of PTB-patients).

10. **Reviewer 4:** Indicate where the pre-test of the questionnaire was conducted and the number of participants involved.

**Author response**: Thank you for the valuable comment. We accepted the suggestion and clearly indicated in the Methods section that the questionnaire was pre-tested on 5% of the total sample size. The pre-test was conducted in a similar setting among participants who were not included in the final study to ensure clarity, consistency, and validity of the data collection tool.

11. **Reviewer 4:** Please include 95% confidence intervals (CIs) in Table 2 to facilitate comparison with other studies in the discussion section.

**Author response**: Thank you for this helpful suggestion. We accepted the comment and incorporated the 95% confidence intervals (95% CIs) for the main variables in Table 2. This addition was made to improve the interpretability of the results and to facilitate direct comparison with findings from other studies in the discussion section.

12. **Reviewer 4:** Clarify how independent variables were selected for bivariable and multivariable logistic regression.

**Author response**: Each of the variables were run in the SPSS version 28 software independently in the bivariable logistic regression to select the candidate variables (p≤ 0.25) for multivariable logistic regression (MLR). All variables which have a p-value ≤0.25 were considered as candidate for MLR analysis and all run into a MLR at once. The statistically significant variables were considered based on p<0.05 and 95%CI.

13. **Reviewer 4:** It is methodologically inappropriate to analyze sociodemographic variables separately from clinical and behavioral factors in multivariable models.

**Author response**: Thank you for the constructive comment. The candidate variables for the multivariable logistic regression analyses presented in Tables 3 and 4 were selected and entered concurrently into the multivariable model at once based on the predefined selection criteria from the bivariable analysis. However, the tables were presented separately to improve clarity and readability of the results for PTB associated factors.

14. **Reviewer 4:** Variables with p-value ≤0.25 in the bivariable analysis should be entered together into a single multivariable logistic regression model.

**Author response**: We performed a single multivariable logistic regression model for all candidate variables for associated factor analyses.

15. **Reviewer 4:** Tables 3 and 4 should ideally be merged into one comprehensive table. If separated for clarity, ensure that all variables are included in a unified multivariable model.

**Author response**: Thank you for the constructive comment. The candidate variables for the multivariable logistic regression analyses presented in Tables 3 and 4 were selected and entered concurrently into the multivariable model at once based on the predefined selection criteria from the bivariable analysis. However, the tables were presented separately to improve clarity and readability of the results for PTB associated factors.

16. **Reviewer 4:** There are inconsistencies between the table and its description. For example: The reference category for age is listed as 8-15 years in the table, whereas the text compares ≥ 66 years with 16-30 years. This inconsistency must be corrected.

**Author response**: Thank you for the important observation. We accepted the comment and corrected the inconsistencies between the tables and their corresponding descriptions. The reference categories were revised to ensure consistency in the result and discussion sections of the manuscript.

17. **Reviewer 4:** The finding that participants with income <3000 birr have lower odds of infection compared to those earning >6000 birr (AOR: 0.53; 95%CI: 0.29-0.94) requires careful interpretation. Please verify the reference category and ensure the correctness of the statistical analysis.

**Author response**: Thank you for the important observation. The reference categories were retested and revised to ensure consistency throughout the manuscript, and the interpretations in the discussion section were carefully corrected accordingly.

18. **Reviewer 4:** Table 4, for the variable “type of health facility visited,” specify the categories included under “others” in a footnote, or revise the reference category for clarity.

**Author response**: Thank you for the constructive comment. We accepted the suggestion and removed the variable “type of health facility visited” from Table 4, considering it less important to the main objectives and findings of this manuscript.

Finally, once again we would like to thank you the editor and reviewers for your time and expertise.

In case of any questions and doubts, please do not hesitate to contact us anytime.

With best regards,

Tebelay Dilnessa (MSc, PhD candidate in Medical Microbiology, University of Gondar,

Email: [tebelay@gmail.com](mailto:tebelay@gmail.com); Telephone: +251912198715)

Onbehaf of all authors
