# Supplementary material for: Rifampicin resistance and associated factors of Mycobacterium tuberculosis among pulmonary tuberculosis-suspected patients in the Amhara National Regional State Comprehensive Specialized Hospitals, Ethiopia
Source: PLoS One. 2026 Jun 26;21(6):e0351964. doi: 10.1371/journal.pone.0351964 (PMC13309019; doi:10.1371/journal.pone.0351964)
Supplement: Attachment — Submitted filename: Comments and questions TD Manuscript PONE-D-26-17510.docx [file pone.0351964.s002.docx]

**Manuscript title:** Rifampicin resistance and associated factors of Mycobacterium tuberculosis among pulmonary tuberculosis-suspected patients in the Amhara National Regional State Comprehensive Specialized Hospitals, Ethiopia

Dear editor,

In this manuscript, and based on its content and its context, the author raised an important issue and tried to address the most global, regional and national sensitive issues, rifampicin resistance M. tuberculosis in the Amhara National Regional State. The study is comprehensive as it is conducted in a multifacility based by considering the three largest comprehensive and specialized hospitals found in the region, and using a very large sample size. The document (including the introduction, the methods, the discussion…) is detail, well written, well organized, or structured. How ever, the manuscript has some issues (editorial issues, some comments, and few questions) that need to be addressed before published in the journal. Some of the most important comments and questions have been addressed in the reviewer session for the author. Moreover, it has been given as a track change in the main manuscript for correction by the authors.

Decision of the reviewer: **Accept with minor revision**

**Comments**

A. **Minor**
I. **In the abstract:**

- Make this objective as part of the background or put at the end, by stating like: Therefore, this study was aimed….
- Re-estate the result as given in the document, and remove unnecessary details as stated or corrected by the track changes
- Remove the numbers/the percentage details in the conclusion part and the key word “Tuberculosis” in the key word

II. **Introduction**

Consider the use of terms “several studies and putting one reference at the end; need consideration or inclusion of references

Check some statements without reference

III. **Methods part**

In the study sites, better to abbreviate once and use in the rest part of the document

On line 146 to 148; it is better to put your references where your questionnaire is adapted.

Line 169 to 170: Remove as it is a reptation from the above sentence (Variables with p-value less than or equal to 0.25 in the bivariate analysis were jointly entered into a multivariate logistic regression analysis)

Line 172; Better to include a statement like: Finally, the data was presented using texts, figures…

Line 224; on the result part of the sub title: As you were studied on PTB, it is better to say: Associated factors for PTB infection

In assessing the variables, of the two tables, you better to indicate those p-values <0.05 with * and give the key at the bottom

**In the limitation part**;

- “The study included patients from selected comprehensive specialized hospitals, which may not represent the broader population in the Amhara National Regional State, particularly rural areas or those without access to advanced health facilities” may not be the limitations of your study. This is because these hospitals are those referral and specialized hospitals, where the samples are referred from the rural health centers to these hospitals too.

**In the conclusion part;**

In steady of writing these numbers here in the conclusion part, it better to re-state for what the observed finding/s mean

**In the abbreviation part:**

If you abbreviate e.g. DMCSH, you need to use in the document

B. **Major comments**

**On the result part**

I. The result part: If you have a table at the end, you better state the main or those variables having the highest only, do not put all the details

II. Check the numbers or values in the table 3 and table 4 including the rules you stated in the methods, analysis, part (p-value <0.25 to be fitted to the MVLR analysis)

**On the discussion part:**

III. Your discussion is too much, remove unnecessary details or better to write to the point. You should put your findings, followed by implications, if possible, then compare with the previous findings and give the possible explanations/justifications for the observed variations

IV. To compare your findings with the previous studies, you better show the 95%Cis for your findings including for the drug resistance part.

**Questions**

1. What is your justification for the exclusion of age < 8 years?

2. What is your justification to consider 150 positives why not 100 or 200?

3. Why you operationally define this??? Is it your interest or your objective to assess the resistance of isoniazid or MDR?

.
