# Supplementary material for: Rifampicin resistance and associated factors of Mycobacterium tuberculosis among pulmonary tuberculosis-suspected patients in the Amhara National Regional State Comprehensive Specialized Hospitals, Ethiopia
Source: PLoS One. 2026 Jun 26;21(6):e0351964. doi: 10.1371/journal.pone.0351964 (PMC13309019; doi:10.1371/journal.pone.0351964)
Supplement: Attachment — Submitted filename: Main manuscript track changes TD PONE-D-26-17510.docx [file pone.0351964.s003.docx]

PLOS One

**Rifampicin resistance and associated factors of Mycobacterium tuberculosis among pulmonary tuberculosis-suspected patients in the Amhara National Regional State Comprehensive Specialized Hospitals, Ethiopia**

--Manuscript Draft--

| **Manuscript Number:** | PONE-D-26-17510 |
| --- | --- |
| **Article Type:** | Research Article |
| **Full Title:** | Rifampicin resistance and associated factors of Mycobacterium tuberculosis among pulmonary tuberculosis-suspected patients in the Amhara National Regional State Comprehensive Specialized Hospitals, Ethiopia |
| **Short Title:** | Rifampicin resistance of Mycobacterium tuberculosis among pulmonary tuberculosis- suspected patients |
| **Corresponding Author:** | Tebelay Dilnessa, MSc University of Gondar Debre Markos, ETHIOPIA |
| **Keywords:** | Rifampicin resistance, Mycobacterium tuberculosis, GeneXpert |
| **Abstract:** | Background: Tuberculosis (TB) remains a major public health challenge globally, with drug-resistant strains, particularly rifampicin-resistant Mycobacterium tuberculosis (RR- MTB), which poses serious challenges for treatment and control. Ethiopia is among the high TB burden countries, and the Amhara National Regional State continues to report significant tuberculosis morbidity and mortality.  Objective: To determine the prevalence of rifampicin-resistant M. tuberculosis and associated factors among tuberculosis-suspected patients at the Amhara National Regional State Comprehensive Specialized Hospitals, Northwest Ethiopia.  Methods: A multicenter cross-sectional study was conducted among pulmonary tuberculosis-suspected patients attending comprehensive specialized hospitals from April 01, 2023, to May 30, 2025. Socio-demographic and associated factor data were collected using semi-structured questionnaires. Sputum was collected and tested using GeneXpert MTB/RIF assay to detect M. tuberculosis and rifampicin resistance. Data were entered and analyzed using SPSS version 28. Binary logistic regression was applied to assess the relationship between predictors and the outcome variable. Variables with p-value of ≤ 0.05 at 95%CI in multivariate logistic regression were considered statistically significant.  Results: Out of 2,548 pulmonary tuberculosis (PTB)-suspected participants, 150 (5.9%), were positive for M. tuberculosis The prevalence was higher in males (6.4%) than females (5.4%), in urban (7.0%) compared to rural (5.2%) residents, and among participants aged > 66 years (11.2%). Rifampicin resistance was detected in 19/150 (12.7%) of M. tuberculosis-positive cases, more frequently among urban residents (18.6%), and among clients with low education 20.0% (in grades 1-4). In multivariate logistic regression analysis, age, residence, occupation, income, and marital status were associated with M. tuberculosis infection (p< 0.05). Rural residence was associated with reduced pulmonary tuberculosis infection compared with urban residence (AOR: 0.23; 95%CI: 0.06-0.79). Students had markedly lower odds of PTB (AOR: 0.03; 95%CI: 0.01-0.11). Previous antibiotic use (AOR: 9.17; 95%CI: 2.01-  28.14) and alcohol consumption (AOR: 7.10; 95%CI: 4.99-20.86) were independently associated with increased M. tuberculosis infection.  Conclusion: The prevalence of M. tuberculosis infection and rifampicin resistance remain significant in the study area. The strong association between prior antibiotic use and alcohol consumption with M. tuberculosis highlights the need for targeted case-finding, improved diagnosis, rational antibiotic use, and focused health education interventions. |
| **Order of Authors:** | Tebelay Dilnessa, MSc |
|  | Feleke Moges, PhD, Professor |
|  | Belay Tessema, PhD, Professor |
|  | Workagegnehu Hailu, MD+, Associate Professor |
|  |  |

|  | Baye Gelaw, PhD, Professor |
| --- | --- |
| **Additional Information:** |  |
| **Question** | **Response** |
| **Financial Disclosure**  Enter a financial disclosure statement that describes the sources of funding for the work included in this submission. Review the [submission guidelines](http://journals.plos.org/plosone/s/submission-guidelines#loc-financial-disclosure-statement) for detailed requirements. View published research articles from [*PLOS ONE*](https://journals.plos.org/plosone/) for specific examples.  This statement is required for submission and **will appear in the published article** if the submission is accepted. Please make sure it is accurate. | The author(s) received no specific funding for this work. |
| **Funded studies**  Enter a statement with the following details:   - Initials of the authors who received each award - Grant numbers awarded to each author - The full name of each funder - URL of each funder website - Did the sponsors or funders play any role in the study design, data collection and analysis, decision to publish, or preparation of the manuscript? |  |
| Did you receive funding for this work? |  |
| **Competing Interests**  Use the instructions below to enter a competing interest statement for this submission. On behalf of all authors, disclose any [competing interests](http://journals.plos.org/plosone/s/competing-interests) that could be perceived to bias this work—acknowledging all financial support and any other relevant financial or non- financial competing interests.  This statement is required for submission and **will appear in the published article** if the submission is accepted. Please make sure it is accurate and that any funding | The authors have declared that no competing interests exist. |

| sources listed in your Funding Information later in the submission form are also declared in your Financial Disclosure statement.  View published research articles from  [*PLOS ONE*](http://journals.plos.org/plosone/) for specific examples. |  |
| --- | --- |
| **NO authors have competing interests**  Enter: *The authors have declared that no competing interests exist.*  **Authors with competing interests**  Enter competing interest details beginning with this statement:  *I have read the journal's policy and the authors of this manuscript have the following competing interests: [insert competing interests here]* |  |
| * typeset |  |
| **Ethics Statement**  Enter an ethics statement for this submission. This statement is required if the study involved:   - Human participants - Human specimens or tissue - Vertebrate animals or cephalopods - Vertebrate embryos or tissues - Field research   Write "N/A" if the submission does not require an ethics statement.  General guidance is provided below. Consult the [submission guidelines](http://journals.plos.org/plosone/s/submission-guidelines#loc-guidelines-for-specific-study-types) for detailed instructions. **Make sure that all information entered here is included in the Methods section of the manuscript.** | N/A |

| **Format for specific study types**  **Human Subject Research (involving human participants and/or tissue)**   - Give the name of the institutional review board or ethics committee that approved the study - Include the approval number and/or a statement indicating approval of this research - Indicate the form of consent obtained (written/oral) or the reason that consent was not obtained (e.g. the data were analyzed anonymously)   **Animal Research (involving vertebrate animals, embryos or tissues)**   - Provide the name of the Institutional Animal Care and Use Committee (IACUC) or other relevant ethics board that reviewed the study protocol, and indicate whether they approved this research or granted a formal waiver of ethical approval - Include an approval number if one was obtained - If the study involved *non-human primates*, add *additional details* about animal welfare and steps taken to ameliorate suffering - If anesthesia, euthanasia, or any kind of animal sacrifice is part of the study, include briefly which substances and/or methods were applied   **Field Research**  Include the following details if this study involves the collection of plant, animal, or other materials from a natural setting:   - Field permit number - Name of the institution or relevant body that granted permission |  |
| --- | --- |
|  |  |
| <strong>Data Availability</strong><br>  <br>Before publication, Authors are required to make fully available and without restriction all data underlying their findings. Sharing the data underlying your findings increases the visibility, credibility, and impact of your work, while supporting transparency and future scientific discovery. Please see our <a href="https://journals.plos.org/plosone/s/d ata-availability" target="_blank">PLOS Data Policy page</a> for detailed information on this policy.</p>  <br>A <strong>Data Availability | All relevant data are within the manuscript and its supporting information files. |

| Statement</strong>, detailing where the data can be accessed, is required at first submission. Insert your Data Availability Statement in the box below.</p>  <br>Please see the <a href="https://journals.plos.org/plosone/s/s ubmission-guidelines#loc-data-reporting" target="_blank">data reporting</a> of our submission guidelines for instructions on what you need to include in your Data Availability Statement. Examples of acceptable Data Availability Statements are included here for reference:</p>  <ol>  <li>The minimal data set [and accompanying code, where generated] is available at [repository name] via [DOI hyperlink]</li>  <li>No data was generated by this study. The following existing data sources were used: [dataset name] from [dataset location] available via [web address or DOI hyperlink].</li>  <li>For Study Protocols: No datasets were generated or analysed during the current study. All relevant data from this study will be made available upon study completion.</li>  </ol>  <br><i>PLOS allows rare exemptions to address legal and ethical concerns. If you have legal or ethical restrictions, please use the box below to detail these in full sentences for the Journal team to consider.</i></p>  <br>This statement will be typeset if the manuscript is accepted for publication.</p> |  |
| --- | --- |

Manuscript	[Click here to access/download;Manuscript;Manuscript.docx](https://www2.cloud.editorialmanager.com/pone/download.aspx?id=44213238&guid=8bad020f-a32a-4374-b8e9-e2936acb5ea7&scheme=1)[<https://www2.cloud.editorialmanager.com/pone/download.aspx?id=44213238&guid=8bad020f-a32a-4374-b8e9-e2936acb5ea7&scheme=1>](https://www2.cloud.editorialmanager.com/pone/download.aspx?id=44213238&guid=8bad020f-a32a-4374-b8e9-e2936acb5ea7&scheme=1)

1 **Rifampicin resistance and associated factors of *Mycobacterium tuberculosis* among pulmonary**
2 **tuberculosis-suspected patients in the Amhara National Regional State Comprehensive**
3 **Specialized Hospitals, Ethiopia**
4 Tebelay Dilnessa ^a,^ ^b^*, Feleke Moges ^a^, Belay Tessema ^a,^ ^c^, Workagegnehu Hailu ^d^, Baye Gelaw ^a^
5 ^a^ Department of Medical Microbiology, School of Biomedical and Laboratory Sciences, College of Medicine
6 and Health Sciences, University of Gondar; P. O. Box 196, Gondar, Ethiopia
7 ^b^ Department of Medical Laboratory Science, College of Medicine and Health Sciences, Debre Markos
8 University; P. O. Box 269, Debre Markos, Ethiopia
9 ^c^ Institute of Clinical Immunology, Faculty of Medicine, University of Leipzig; 04103, Leipzig, Germany
10 ^d^ Department of Internal Medicine, School of Medicine, College of Medicine and Health Sciences, University
11 of Gondar; P. O. Box 196, Gondar, Ethiopia

12

13

14

15

16	*Corresponding author: Tebelay Dilnessa: [tebelay@gmail.com](mailto:tebelay@gmail.com), Telephone: +251912198715

17

18

19

20

21

22

23

24

25

26

27

28

29

30

# 31 Abstract

32 **Background:** Tuberculosis (TB) remains a major public health challenge globally, with drug-resistant strains,
33 particularly rifampicin-resistant *Mycobacterium tuberculosis* (RR-MTB), which poses serious challenges for
34 treatment and control. Ethiopia is among the high TB burden countries, and the Amhara National Regional
35 State continues to report significant tuberculosis morbidity and mortality.
36 **Objective:** To determine the prevalence of rifampicin-resistant *M. tuberculosis* and associated factors among
37 tuberculosis-suspected patients at the Amhara National Regional State Comprehensive Specialized Hospitals,
38 Northwest Ethiopia.
39 **Methods:** A multicenter cross-sectional study was conducted among pulmonary tuberculosis-suspected
40 patients attending comprehensive specialized hospitals from April 01, 2023, to May 30, 2025. Socio-
41 demographic and associated factors data were collected using semi-structured questionnaires. Sputum was
42 collected and tested using GeneXpert MTB/RIF assay to detect *M. tuberculosis* and rifampicin resistance. Data were entered into and analyzed using SPSS version 28 for analysis. Binary logistic regression was applied to assess the relationship
43 between predictors and the outcome variable. Variables with *p*-value of ≤ 0.05 at 95%CI in multivariate logistic
44 regression were considered statistically significant.
45 **Results:** Out of Among 2,548 pulmonary tuberculosis (PTB)-suspected participants, 150 (5.9%), the overall prevalence of were positive for M. tuberculosis was 150/2548 (5.9%).. The prevalence was higher in males (6.4%), in urban
46 (7.0%) residents, and among participants aged ≥ 66 years (11.2%). Rifampicin
47 resistance was detected in 19/150 (12.7%) of *M. tuberculosis*-positive cases, more frequently among urban
48 residents (18.6%), and among clients with low education 20.0% (in grades 1-4). In multivariate logistic
49 regression analysis, age, residence, occupation, income, and marital status were associated with *M. tuberculosis*
50 infection (*p*< 0.05). Rural residence was associated with reduced pulmonary tuberculosis infection compared
51 with urban residence (AOR: 0.23; 95%CI: 0.06-0.79). Students had markedly lower odds of PTB (AOR: 0.03;
52 95%CI: 0.01-0.11). Previous antibiotic use (AOR: 9.17; 95%CI: 2.01-28.14) and alcohol consumption (AOR:
53 7.10; 95%CI: 4.99-20.86) were independently associated with increased *M. tuberculosis* infection.
54 **Conclusion:** The prevalence of *M. tuberculosis* infection and rifampicin resistance remain
55 significant in the study area. The strong association between prior antibiotic use and alcohol consumption with
56 *M. tuberculosis* highlights the need for targeted case-finding, improved diagnosis, rational antibiotic use, and
57 focused health education interventions.
58 **Keywords:**, Rifampicin resistance, *Mycobacterium tuberculosis*, GeneXpert

61

62

# 63 1. Introduction

64 Tuberculosis (TB), caused by *Mycobacterium tuberculosis* (Mtb), is a major cause of morbidity and mortality
65 worldwide, particularly in low- and middle-income countries. According to the World Health Organization
66 (WHO), TB remains among the top 10 causes of death globally and the leading cause of death from a single
67 infectious agent, surpassing HIV/AIDS [1]. The emergence of drug-resistant *M. tuberculosis*, especially
68 rifampicin-resistant TB (RR-TB) and multidrug-resistant TB (MDR-TB), has become a significant threat to
69 global PTB control efforts [2].
70 Ethiopia is among the 30 high TB and MDR-TB burden countries [1]. The Amhara National Regional State is
71 one of the most populous administrative regions in Ethiopia that consistently reported high number of PTB
72 cases [3]. Delayed diagnosis and poor treatment adherence contribute to the development and spread of drug-
73 resistant strains [4]. The region is one of the most TB-affected areas compounded with different contributing
74 factors such as malnutrition, displacement and instability. In recent years, the region reported increasing rates
75 of both PTB and drug-resistant TB underscoring the urgency for continued research and targeted interventions
76 [5].
77 Rifampicin resistance is of particular concern due to its role as a key first-line anti-TB drug and its use as a
78 proxy marker for MDR-TB. Rifampicin resistance in *M. tuberculosis* remains a major public health challenge
79 due to its strong correlation with multidrug-resistant tuberculosis (MDR-TB). It poses a serious threat to TB
80 control efforts due to limited treatment options and increased morbidity [1, 6]. Accurate and rapid detection
81 of *M. tuberculosis* and its resistance to rifampicin is critical for timely initiation of appropriate therapy and to
82 prevent further transmission. Molecular diagnostic tools such as GeneXpert MTB/RIF facilitate rapid
83 simultaneous detection of *M. tuberculosis* and rifampicin resistance [7, 8].
84 Several studies have documented that sociodemographic factors (such as low educational status,
85 unemployment, and poor socioeconomic conditions), clinical factors (including HIV co-infection, previous TB
86 treatment, and comorbidities), and behavioral characteristics (such as smoking, alcohol use, and treatment non-
87 adherence) significantly influence the development and transmission of rifampicin-resistant *M. tuberculosis*
88 [9]. These associated factors often interact, leading to delayed diagnosis, incomplete treatment, and subsequent
89 amplification of drug resistance. Multiple associated factors contribute to both TB incidence and rifampicin
90 resistance in Amhara National Regional State, Ethiopia [10].
91 In Ethiopia, pulmonary tuberculosis (PTB) remains a leading cause of illness and death, with rifampicin
92 resistance emerging as a major public health concern. The prevalence of rifampicin resistance varies across
93 regions, and both individual patient characteristics and broader contextual factors have been shown to
94 contribute to its development [11]. In the Amhara National Regional State, where the dual burden of high PTB
95 prevalence and increasing rifampicin resistance exists, the need for focused research is particularly critical.
96 Despite the availability of diagnostic tools, there is still limited evidence that comprehensively examines the
97 determinants of rifampicin resistance in this region, especially among PTB-suspected patients attending
98 comprehensive specialized hospitals. Generating such evidence is essential for informing targeted
99 interventions, including patient education, strategies to improve treatment adherence, and tailored public health
100 measures. Therefore, this study aimed to determine the prevalence of *M. tuberculosis* infection and rifampicin
101 resistance, and to identify associated factors among PTB-suspected patients attending selected comprehensive
102 specialized hospitals in the Amhara National Regional State, Ethiopia.

# 103 2. Material and methods

104 **1.2. Study area and setting**
105 The study was conducted among pulmonary tuberculosis-suspected patients at selected comprehensive
106 specialized hospitals of the Amhara National Regional State, Northwest Ethiopia. The hospitals included in the
107 study were Debre Markos Comprehensive Specialized Hospital, Felege-Hiwot Comprehensive Specialized
108 Hospital and University of Gondar Comprehensive Specialized Hospital which serve as referral centers for a
109 wide catchment population. Additionally, these hospitals provide diagnostic and treatment services for PTB
110 patients, making them ideal settings for assessing *M. tuberculosis*-prevalence and resistance patterns.

# 111 2.2. Study design and period

112 A multifacility-based cross-sectional study was conducted from April 01, 2023, to May 30, 2025.

# 113 2.3. Study population

114 The study population consisted of patients aged ≥ 8 years who were suspected of having pulmonary
115 tuberculosis and referred for GeneXpert testing during the study period. Pulmonary tuberculosis suspicion was
116 based on national guidelines, including persistent cough, weight loss, fever, and night sweats.

# 117 2.4. Study variables

118 **2.4.1. Dependent variables**
119 •	Prevalence of *M. tuberculosis*
120 •	Rifampicin susceptibility patterns of *M. tuberculosis*

# 121 2.4.2. Independent variables

122 •	Sociodemographic variables: Age, sex, residence (urban/rural), educational status, occupation
123 •	Clinical and health-related factors: HIV status, health facility visited, distance from health facility,
124 history of PTB patient in the family and contact history, presence of chronic illnesses (e.g., diabetes,
125 hypertension, malnutrition), smoking history, and alcohol consumption

# 126 2.5. Eligibility criteria

127 **2.5.1. Inclusion criteria**
128 •	Individuals diagnosed with PTB based on clinical, and laboratory findings (GeneXpert).
129 •	Patients with complete clinical data and who provided adequate sputum samples.
130 •	Patients aged greater than or equal to 8 years attending at the Amhara National Regional State
131 Comprehensive Specialized Hospitals.

# 132 2.5.2. Exclusion criteria

133 •	Patients with extrapulmonary TB or those on multi-drug-resistant TB (MDR-TB) treatment.
134 •	Patients who have received antibiotics including anti-TB drugs within the past two weeks prior to
135 sample collection.
136 •	Patients who were critically ill and unable to provide sputum sample.

# 137 2.6. Sample size determination and sampling technique

138 The sample size was determined by enrolling participants consecutively until 150 *M. tuberculosis*-positive
139 cases were obtained from the three health facilities. To ensure balanced representation and facilitate
140 comparison across study sites, 50 *M. tuberculosis*-positive participants were included from each health facility.

# 141 Operational definitions

142 **Multidrug-resistant tuberculosis (MDR-TB)**: Defined as *M. tuberculosis* that is resistant to at least isoniazid
143 and rifampin, the two most potent tuberculosis drugs [12].

# 144 2.7. Data collection and processing

145 **2.7.1. Sociodemographic data collection**
146 Socio-demographic and clinical data were collected using a semi-structured and pre-tested questionnaire
147 administered by trained healthcare personnels. Information included were age, sex, residence, TB history, HIV
148 status, contact history, alcohol drinking habit, health facility visited, and smoking status.

# 149 2.7.2. Sputum sample collection

150 Sputum was collected using a sterile, leak-proof, wide-mouthed 50 mL falcon tube from each participant.
151 Patients were trained on the importance of high-quality sputum specimen and instructed to collect the sputum
152 sample. Emphasis was given on the need to produce sputum originating from the lungs, rather than saliva.

153

154

# 155 2.7.3. Laboratory investigation

156 The GeneXpert MTB/RIF assay was run according to the manufacturer’s instructions. The assay detects both
157 *M. tuberculosis* DNA and mutations in the rpoB gene associated with rifampicin resistance. The GeneXpert
158 assay is based on real-time polymerase chain reaction (PCR) technology, which detects the DNA of *M.*
159 *tuberculosis* complex and mutations in the rpoB gene associated with resistance to rifampicin [13]. Two mL
160 sputum samples were mixed with 4 mL buffer, vortex mixed, and incubated at room temperature for 15
161 minutes. Two milliliters of the homogenized sample was then transferred into the GeneXpert cartridge, which
162 was inserted into the machine after barcode registration. Results were generated within 90 minutes.

# 163 2.8. Data analysis and interpretations

164 Data were entered and analyzed using SPSS version 28 computer software. Descriptive data analysis was
165 conducted to organize, summarize, and interpret the data. Bivariable logistic regression analysis was employed
166 to examine the association between independent variables with tuberculosis or rifampicin resistance. A *p*-value
167 ≤ 0.25 was used as a cut-off for inclusion into the multivariate model. Multicollinearity among the independent
168 variables was assessed using the Variance Inflation Factor (VIF) prior to multivariable logistic regression
169 analysis, with a VIF value < 5 indicating no significant multicollinearity. Variables with *p-*value less than or
170 equal to 0.25 in the bivariate analysis were jointly entered into a multivariate logistic regression analysis. A *p*-
171 value ≤ 0.05 with 95%CI was considered significantly associated with *M. tuberculosis* and/or rifampicin
172 resistance. Model fitness was evaluated using the Hosmer-Lemeshow goodness-of-fit test.

# 173 2.9. Ethical considerations

174 The study was conducted in accordance with the principles of the Declaration of Helsinki. Ethical clearance
175 was obtained from the Institutional Review Board (IRB) of College of Medicine and Health Sciences of
176 University of Gondar (Ref. number: CMHS/SH/CS/CoMEng/06/216/3/2015). Written permission was
177 obtained from Debre Markos, Felege-Hiwot and University of Gondar Comprehensive Specialized Hospitals
178 for the data collection process. Written informed assent and consent were obtained from the parents/guardians
179 and study participants, respectively. Confidentiality and anonymity were also maintained, and participants
180 received counseling and appropriate referral when necessary.

# 181 3. Results

182 **3.1. Socio-demographic characteristics of the study participants**
183 A total of 2,548 tuberculosis-suspected participants were enrolled in this study, of whom 46.7% were males
184 and 53.3% were females. The dominant age group was 16-30 years (36.9%), followed by 41-65 years (20.6%).
185 The majority of participants were living in rural areas (60.5%). In terms
186 of study site distribution, 35.5% were from Felege-Hiwot, 33.8% from Debre Markos, and 30.7% from
187 University of Gondar Comprehensive Specialized Hospitals. Data on educational status of the study
188 participants showed that 28.0% were illiterate, 20.2% completed elementary school, and 18.8% attained
189 college or university. Farmers were predominant (40.8%) followed by merchants (20.3%), and government
190 employees (17.8%). More than half of the study participants (62.3%) had a family size greater than five and
191 the proportion of the study participants who had monthly income less than 3,000 Ethiopian birr (ETB) was
192 33.5%, those with 3,001-6,000 ETB (55.6%), and 10.9% earned above 6,001 ETB (Table 1).
193 **Table 1**: Socio-demographic characteristics and the proportion of *M. tuberculosis* among pulmonary
194 tuberculosis-suspected participants in the Amhara National Regional State Comprehensive Specialized
195 Hospitals, Ethiopia (N=2,548)

| **Variables** | **Categories of variables** | **Tuberculosis status** | | | **Total, n (%)** |
| --- | --- | --- | --- | --- | --- |
|  |  | **Positive, n (%)** | **95%CI** | **Negative, n (%)** |  |
| Sex | Male | 76 (6.4) | 5.0-7.8 | 1114 (93.6) | 1190 (46.7) |
|  | Female | 74 (5.4) | 4.2-6.7 | 1284 (94.6) | 1358 (53.3) |
| Age group (in years) | 8-15 | 22 (6.1) | 3.6-8.6 | 339 (93.9) | 361 (14.2) |
|  | 16-30 | 32 (3.4) | 2.3-4.6 | 907 (96.6) | 939 (36.9) |
|  | 31-40 | 28 (6.6) | 4.2-8.9 | 398 (93.4) | 426 (16.7) |
|  | 41-65 | 35 (6.7) | 4.5-8.8 | 491 (93.3) | 526 (20.6) |
|  | ≥ 66 | 33 (11.2) | 7.6-14.7 | 263 (88.8) | 296 (11.6) |
| Residence | Urban | 70 (7.0) | 5.4-8.5 | 938 (93.0) | 1008 (39.5) |
|  | Rural | 80 (5.2) | 4.1-6.3 | 1460 (94.8) | 1540 (60.5) |
| Study area | University of Gondar CSH | 50 (6.4) | 4.7-8.1 | 732 (93.6) | 782 (30.7) |
|  | Felege-Hiwot CSH | 50 (5.5) | 4.0-7.0 | 854 (94.5) | 904 (35.5) |
|  | Debre Markos CSH | 50 (5.8) | 4.2-7.4 | 812 (94.2) | 862 (33.8) |
| Educational status | Illiterate | 43 (6.0) | 4.3-7.8 | 671 (94.0) | 714 (28.0) |
|  | Grade 1-4 | 15 (3.3) | 1.6-4.9 | 446 (96.7) | 461 (18.1) |
|  | Grade 5-8 | 24 (4.7) | 2.8-6.5 | 490 (95.3) | 514 (20.2) |
|  | Grade 9-12 | 27 (7.1) | 4.5-9.7 | 353 (92.9) | 380 (14.9) |
|  | College & above | 41 (8.6) | 6.1-11.1 | 438 (91.4) | 479 (18.8) |
| Occupation | Government employed | 33 (7.3) | 4.5-9.7 | 420 (92.7) | 453 (17.8) |
|  | Merchant | 41 (7.9) | 5.6-10.3 | 475 (92.1) | 516 (20.3) |
|  | Farmer | 61 (5.9) | 4.3-7.3 | 980 (94.1) | 1041 (40.8) |

|  | Daily laborer | 2 (2.1) | 0.8-5.0 | 92 (97.9) | 94 (3.7) |
| --- | --- | --- | --- | --- | --- |
|  | Student | 4 (1.0) | 0.2- 2.0 | 398 (99.0) | 402 (15.8) |
|  | Others | 9 (21.4) | 9.0-33.8 | 33 (78.6) | 42 (1.6) |
| Family size | < 5 | 60 (6.2) | 4.7-7.8 | 901 (93.8) | 961 (37.7) |
|  | ≥ 5 | 90 (5.7) | 4.5-6.8 | 1497 (94.3) | 1587 (62.3) |
| Income (ETB)* | ≤ 3,000 | 44 (5.2) | 3.7-6.6 | 809 (94.8) | 853 (33.5) |
|  | 3,001-6,000 | 77 (5.4) | 4.3-6.6 | 1339 (94.6) | 1416 (55.6) |
|  | ≥ 6,001 | 29 (10́.4) | 6.8-14.0 | 250 (89.6) | 279 (10.9) |
| Marital status | Married | 91 (7.3) | 5.8-8.7 | 1163 (92.7) | 1254 (49.2) |
|  | Single | 48 (4.1) | 2.9-5.2 | 1133 (95.9) | 1181 (46.4) |
|  | Divorced | 5 (10.6) | 1.8-19.5 | 42 (89.4) | 47 (1.8) |
|  | Widowed | 6 (9.1) | 2.2-16.0 | 60 (90.9) | 66 (2.6) |
| **Total** | | **150 (5.9)** | **5.0- 6.9** | **2398 (94.1)** | **2548 (100.0)** |

196

197

198

199

200

201

202

203

204

205

206

207

208

209

210

211

212

213

CSH: Comprehensive Specialized hospitals; ETB: Ethiopian Birr

3.2. **Prevalence of *Mycobacterium tuberculosis***

The overall prevalence of *M. tuberculosis* among the participants was 5.9% (150/2,548) (5.0-6.9). The prevalence was slightly higher in males (6.4%),among participants ≥66 years (11.2%), from urban areas (7.0%) in University of Gondar comprehensive specialized hospital (6.4%) , . among patients who had college and above education level (8.6%), and , among divorced participants (10.6%) (Table 1).

3.3. **Rifampicin susceptibility pattern of *M. tuberculosis***

Among the 150 *M. tuberculosis* positive participants tested by GeneXpert MTB/RIF assay, rifampicin resistance was detected in 19 (12.7%; 95%CI: 7.4-18.0), while 120 (80.0%; 95%CI: 73.6-86.4) were rifampicin susceptible, and 11 (7.3%; 95%CI: 3.2-11.5) yielded indeterminate results. Rifampicin resistance was slightly higher in females (14.8%) compared to males (10.5%). Across age groups, the highest rifampicin resistance was observed among participants aged 31-40 years (21.4%) followed by those aged 16-30 years (12.5%). Urban residents had a higher resistance rate (18.6%) than rural residents (7.5%). The prevalence of rifampicin

214 resistance was 14.0% in University of Gondar, 12.0% in Felege-Hiwot and Debre Markos comprehensive
215 specialized hospitals each. Data also showed that the proportion of rifampicin resistance was 20.0% among
216 study participants who had lower educational status (grade 1-4) and 16.7% among those with grade 5-8, but
217 14.8% among grade 9-12 educational status. The prevalence of rifampicin-resistant *M. tuberculosis* was 21.9%
218 among merchants but 15.0% among government employees. Married participants had higher proportion of
219 rifampicin resistant *M. tuberculosis* (17.6%) compared to singles (6.3%) participants (Table 2).
220 **Table 2**: Frequency of rifampicin susceptibility pattern of *M. tuberculosis* using GeneXpert MTB/RIF assay
221 among *M. tuberculosis*-positive participants in the Amhara National Regional State Comprehensive
222 Specialized Hospitals, Ethiopia (N=150)

| **Variables** | **Categories of variables** | **Rifampicin susceptibility pattern** | | | **Total, n (%)** |
| --- | --- | --- | --- | --- | --- |
|  |  | **Detected, n (%)** | **Not detected, n**  **(%)** | **Indeterminate,**  **n (%)** |  |
| Sex | Male | 8 (10.5) | 62 (81.6) | 6 (7.9) | 76 (50.7) |
|  | Female | 11 (14.8) | 58 (78.4) | 5 (6.8) | 74 (49.3) |
| Age group (in years) | 8-15 | 1 (4.6) | 18 (81.8) | 3 (13.6) | 19 (14.7) |
|  | 16-30 | 4 (12.5) | 27 (84.4) | 1 (3.1) | 31 (21.3) |
|  | 31-40 | 6 (21.4) | 22 (78.6) | 0 (0.0) | 28 (18.7) |
|  | 41-65 | 4 (11.4) | 28 (80.0) | 3 (8.6) | 35 (23.3) |
|  | ≥ 66 | 4 (12.1) | 25 (75.8) | 4 (12.1) | 33(22.0) |
| Residence | Urban | 13 (18.6) | 52 (74.3) | 5 (7.1) | 70 (46.7) |
|  | Rural | 6 (7.5) | 68 (85.0) | 6 (7.5) | 80 (53.3) |
| Study area | University of Gondar CSH | 7 (14.0) | 39 (78.0) | 4 (8.0) | 50 (33.4) |
|  | Felege-Hiwot CSH | 6 (12.0) | 41 (82.0) | 3 (6.0) | 50 (33.3) |
|  | Debre Markos CSH | 6 (12.0) | 40 (80.0) | 4 (8.0) | 50 (33.3) |
| Educational status | Illiterate | 4 (9.3) | 37 (86.0) | 2 (4.7) | 43 (28.7) |
|  | Grade 1-4 | 3 (20.0) | 11 (73.3) | 1 (6.7) | 15 (10.0) |
|  | Grade 5-8 | 4 (16.7) | 18 (75.0) | 2 (8.3) | 24 (16.0) |
|  | Grade 9-12 | 4 (14.8) | 20 (74.1) | 3 (11.1) | 27 (18.0) |
|  | College & above | 4 (9.8) | 34 (82.9) | 3 (7.3) | 41 (27.3) |
| Occupation | Government employed | 5 (15.2) | 27 (81.8) | 1 (3.0) | 33 (22.0) |
|  | Merchant | 9 (21.9) | 28 (68.3) | 4 (9.8) | 41 (27.3) |
|  | Farmer | 4 (6.6) | 54 (88.5) | 3 (4.9) | 61 (40.7) |

|  | Daily laborer | 1 (50.0) | 1 (50.0) | 0 (0.0) | 2 (1.3) |
| --- | --- | --- | --- | --- | --- |
|  | Student | 0 (0.0) | 3 (75.0) | 1 (25.0) | 4 (2.7) |
|  | Others | 0 (0.0) | 7 (77.8) | 2 (22.2) | 9 (6.0) |
| Family size | < 5 | 12 (20.0) | 46 (76.7) | 2 (3.3) | 60 (40.0) |
|  | ≥ 5 | 7 (7.8) | 74 (82.2) | 9 (10.0) | 90 (60.0) |
| Income (ETB) | ≤ 3,000 | 6 (13.6) | 33 (75.0) | 5 (11.4) | 44 (29.3) |
|  | 3,001-6,000 | 6 (7.8) | 66 (85.7) | 5 (6.5) | 77 (51.4) |
|  | ≥ 6,001 | 7 (24.1) | 21 (72.4) | 1 (3.5) | 29 (19.3) |
| Marital status | Married | 16 (17.6) | 70 (76.9) | 5 (5.5) | 91 (60.7) |
|  | Single | 3 (6.3) | 41 (85.4) | 4 (8.3) | 48 (32.0) |
|  | Divorced | 0 (0.0) | 4 (80.0) | 1 (20.0) | 5 (3.3) |
|  | Widowed | 0 (0.0) | 5 (83.3) | 1 (16.7) | 6 (4.0) |
| **Total** | | **19 (12.7)** | **120 (80.0)** | **11 (7.3)** | **150 (100)** |

223

224

225

226

227

228

CSH: Comprehensive Specialized hospitals; ETB: Ethiopian birr

# 3.4. Associated factors for tuberculosis infection

In the bivariate model, age, educational status, occupation, income, marital status, and residence were associated with *M. tuberculosis* infection. Data also showed that study participants with the age ≥66 years were

1.93 times more likely to be infected with *M. tuberculosis* compared with study participants aged 16-30 years (COR: 1.93; 95%CI: 1.10-3.39).

229 In the multivariate model, age, residence, occupation, income, and marital status remained associated with *M.*
230 *tuberculosis* infection. Rural residence was associated with reduced odds of *M. tuberculosis* infection compared
231 with urban residence (AOR: 0.23; 95%CI: 0.06-0.79). Students had markedly lower odds of *M. tuberculosis*
232 infection (AOR: 0.03; 95%CI: 0.01-0.11) compared with government employed. Being single was also
233 independently associated with lower PTB odds compared with being married (AOR: 0.51; 95%CI: 0.32-0.80)
234 (Table 3).
235 **Table 3:** Bivariate and multivariate logistic regression analysis of socio-demographic factors against *M. tuberculosis* infection among
236 tuberculosis-suspected participants in the Amhara National Regional Sate Comprehensive Specialized Hospitals, Ethiopia (N=2,548)

| **Variables** | | ***M. tuberculosis* status** | | **COR (95%CI)** | **P-value** | **AOR (95%CI)** | **P-value** |
| --- | --- | --- | --- | --- | --- | --- | --- |
|  |  | **Positive, n (%)** | **Negative, n (%)** |  |  |  |  |
| Sex | Male | 76 (6.4) | 1114 (93.6) | 1.18 (0.85-1.65) | 0.316 |  |  |
|  | Female | 74 (5.4) | 1284 (94.6) | 1 |  |  |  |
| Age group (in years) | 8-15 | 22 (6.1) | 339 (93.9) | 1 |  | 1 |  |
|  | 16-30 | 32 (3.4) | 907 (96.6) | 0.54 (0.31-0.95) | 0.032 | 0.06 (0.03-0.12) | *0.001 |
|  | 31-40 | 28 (6.6) | 398 (93.4) | 1.08 (0.61-1.93) | 0.784 | 0.08 (0.04-0.17) | *0.001 |
|  | 41-65 | 35 (6.7) | 491 (93.3) | 1.09 (0.63-1.90) | 0.738 | 0.08 (0.04-0.17) | *0.001 |
|  | ≥ 66 | 33 (11.2) | 263 (88.8) | 1.93 (1.10-3.39) | 0.022 | 0.14 (0.06-0.32) | *0.001 |
| Residence | Urban | 70 (7.0) | 938 (93.0) | 1 |  |  |  |
|  | Rural | 80 (5.2) | 1460 (94.8) | 0.73 (0.53-1.02) | 0.067 | 0.23 (0.06-0.79) | *0.020 |
| Study area | University of Gondar CSH | 50 (6.4) | 732 (93.6) | 1.11 (0.74-1.66) | 0.615 |  |  |
|  | Felege-Hiwot CSH | 50 (5.5) | 854 (94.5) | 0.95 (0.63-1.42) | 0.807 |  |  |
|  | Debre Markos CSH | 50 (5.8) | 812 (94.2) | 1 |  |  |  |
| Educational status | Illiterate | 43 (6.0) | 671 (94.0) | 0.68 (0.44-1.07) | 0.095 | 0.63 (0.26-1.54) | 0.316 |
|  | Grade 1-4 | 15 (3.3) | 446 (96.7) | 0.36 (0.19-0.66) | 0.001 | 0.42 (0.16-1.07) | 0.069 |
|  | Grade 5-8 | 24 (4.7) | 490 (95.3) | 0.52 (0.31-0.88) | 0.015 | 0.54 (0.25-1.20) | 0.130 |
|  | Grade 9-12 | 27 (7.1) | 353 (92.9) | 0.82 (0.49-1.35) | 0.434 | 0.86 (0.42-1.73) | 0.673 |
|  | College & above | 41 (8.6) | 438 (91.4) | 1 |  | 1 |  |
| Occupation | Government employed | 33 (7.3) | 420 (92.7) | 1 |  | 1 |  |
|  | Merchant | 41 (7.9) | 475 (92.1) | 1.09 (0.68-1.77) | 0.669 | 1.24 (0.72-2.14) | 0.425 |
|  | Farmer | 61 (5.9) | 980 (94.1) | 0.79 (0.51-1.23) | 0.298 | 0.86 (0.44-1.71) | 0.679 |
|  | Daily laborer | 2 (2.1) | 92 (97.9) | 0.27 (0.06-1.12) | 0.081 | 0.32 (0.07-1.47) | 0.143 |
|  | Student | 4 (1.0) | 398 (99.0) | 0.13 (0.04-0.36) | 0.001 | 0.03 (0.01-0.11) | *0.001 |
|  | Others | 9 (21.4) | 33 (78.6) | 3.47 (1.53-7.86) | 0.003 | 3.44 (1.28-9.27) | *0.014 |

| Family size | < 5 | 60 (6.2) | 901 (93.8) | 1 |  |  |  |
| --- | --- | --- | --- | --- | --- | --- | --- |
|  | ≥ 5 | 90 (5.7) | 1497 (94.3) | 0.90 (0.64-1.26) | 0.552 |  |  |
| Income (ETB) | ≤ 3,000 | 44 (5.2) | 809 (94.8) | 0.47 (0.28-0.76) | 0.002 | 0.53 (0.29-0.94) | *0.030 |
|  | 3,001-6,000 | 77 (5.4) | 1339 (94.6) | 0.49 (0.32-0.77) | 0.002 | 2.17 (0.65-7.30) | 0.208 |
|  | ≥ 6,001 | 29 (10́.4) | 250 (89.6) | 1 |  | 1 |  |
| Marital status | Married | 91 (7.3) | 1163 (92.7) | 1 |  | 1 |  |
|  | Single | 48 (4.1) | 1133 (95.9) | 0.54 (0.38-0.77) | 0.001 | 0.51 (0.32-0.80) | 0.004 |
|  | Divorced | 5 (10.6) | 42 (89.4) | 1.52 (0.59-3.94) | 0.387 | 1.38 (0.52-3.64) | 0.513 |
|  | Widowed | 6 (9.1) | 60 (90.9) | 1.27 (0.54-3.04) | 0.579 | 0.74 (0.29-1.86) | 0.521 |
| **Total** | | **150 (5.9)** | **2398 (94.1)** |  |  |  |  |

237

238

239

240

241

242

243

244

245

Key: *:……; CSH: Comprehensive Specialized hospitals; AOR: Adjusted odds ratio; COR: Crude odds ratio

# 246 3.5. Association of clinical and behavioral factors with *M. tuberculosis* infection

247 In the bivariate model, several variables were significantly associated with *M. tuberculosis* infection among
248 PTB-suspected patients. A higher likelihood of PTB was observed among participants with previous
249 antibiotic use (COR: 1.63; 95%CI: 1.17-2.26; *p*= 0.004), and those who had a PTB patient in the family
250 (COR: 1.87; 95%CI: 1.34-2.61; *p* = 0.001). Cigarette smoking (COR: 4.72; 95%CI: 3.17-7.03; *p*= 0.001)
251 and alcohol drinking (COR: 12.00; 95%CI: 8.37-17.20; *p*= 0.001) were also strongly associated with PTB-
252 positivity.
253 In the multivariate logistic regression analysis, previous antibiotic use (AOR: 9.17; 95%CI: 2.01-28.14; *p*=
254 0.001) and alcohol drinking (AOR: 7.10; 95%CI: 4.99-20.86; *p*= 0.001) remained independently associated
255 with increased odds of *M. tuberculosis* infection. The type of health facility visited also remained
256 significantly associated with PTB status, with lower odds observed among patients who visited health posts
257 (AOR: 0.02; 95%CI: 0.05-0.12), health centers (AOR: 0.03; 95%CI: 0.06-0.15), government hospitals
258 (AOR: 0.03; 95%CI: 0.07-0.17), and private hospitals/clinics (AOR: 0.03; 95% CI: 0.07-0.18) compared
259 with other facilities (all *p*= 0.001). Other variables, including underlying diseases, cigarette smoking,
260 distance from health facility, source of drinking water, and family history of *M. tuberculosis* infection were
261 not statistically significant after adjustment (Table 4).

262

263

264

265

266 **Table 4:** Bivariate and multivariate regression analysis of associated factors against *M. tuberculosis* infection among tuberculosis-suspected
267 patients in the Amhara National Regional State Comprehensive Specialized Hospitals, Ethiopia (N=2,548)

| **Variables** | | ***M. tuberculosis* status** | | **COR (95%CI)** | **P-value** | **AOR (95%CI)** | **P-value** |
| --- | --- | --- | --- | --- | --- | --- | --- |
|  |  | **Positive, n (%)** | **Negative, n (%)** |  |  |  |  |
| Chief compliant | Cough | 86 (7.9) | 1000 (92.1) | 1.31 (0.51-3.32) | 0.573 | 2.17 (0.75-6.30) | 0.154 |
|  | Fever | 46 (6.6) | 654 (93.4) | 1.07 (0.41-2.77) | 0.891 | 1.77 (0.59-5.25) | 0.305 |
|  | Chest pain | 6 (1.3) | 465 (98.7) | 0.19 (0.06-0.66) | 0.008 | 0.25 (0.05-0.87) | 0.031 |
|  | Weight loss | 3 (1.8) | 168 (98.2) | 0.27 (0.06-1.16) | 0.079 | 0.43 (0.08-2.13) | 0.304 |
|  | Hemoptysis | 4 (10.3) | 35 (89.7) | 1.74 (0.45-6.86) | 0.431 | 0.54 (0.09-3.33) | 0.508 |
|  | Shortness of breath | 5 (6.2) | 76 (93.8) | 1 |  | 1 |  |
| Underlying disease | HIV/AIDS | 52 (15.5) | 283 (84.5) | 1.67 (0.77-3.63) | 0.195 | 2.06 (0.82-5.20) | 0.123 |
|  | Diabetes | 18 (3.3) | 532 (96.7) | 0.69 (0.31-1.55) | 0.377 | 0.89 (0.35-2.20) | 0.813 |
|  | UTI | 18 (3.7) | 473 (96.3) | 0.86 (0.39-1.88) | 0.701 | 1.18 (0.46-2.98) | 0.729 |
|  | Asthma | 19 (3.7) | 501 (96.3) | 0.94 (0.43-2.04) | 0.884 | 0.79 (0.31-1.97) | 0.614 |
|  | Hypertension | 10 (8.1) | 113 (91.9) | 2.09 (0.87-5.00) | 0.097 | 1.92 (0.66-5.60) | 0.230 |
|  | Pneumonia | 16 (7.7) | 193 (92.3) | 1.48 (0.64-3.43) | 0.351 | 1.97 (0.73-5.27) | 0.178 |
|  | Others | 8 (5.0) | 151 (95.0) | 0.89 (0.33-2.38) | 0.824 | 1.49 (0.47-4.67) | 0.494 |
|  | No underlying disease | 9 (5.6) | 152 (94.4) | 1 |  | 1 |  |
| Previous  antibiotics use | Yes | 77 (7.7) | 943 (92.5) | 1.63 (1.17-2.26) | 0.004 | 9.17 (2.01-28.14) | *0.001 |
|  | No | 73 (4.8) | 1455 (95.2) | 1 |  | 1 |  |
| Source of  drinking water | Tap water | 70 (6.9) | 942 (93.1) | 1 |  | 1 |  |
|  | River and others | 80 (5.2) | 1456 (94.8) | 0.74 (0.53-1.03) | 0.074 | 4.50 (0.42-26.32) | 0.997 |
| Family history  of PTB | Yes | 82 (8.0) | 939 (92.0) | 1.87 (1.34-2.61) | 0.001 | 0.02 (0.01-10.53) | 0.998 |
|  | No | 68 (4.5) | 1459 (95.5) | 1 |  | 1 |  |
| Cigarette  smoking | Yes | 39 (19.0) | 166 (81.0) | 4.72 (3.17-7.03) | 0.001 | 1.01 (0.56-1.83) | 0.961 |
|  | No | 111 (4.7) | 2232 (95.3) | 1 |  | 1 |  |
|  | Yes | 68 (30.5) | 155 (69.5) | 12.0 (8.37-17.20) | 0.001 | 7.10 (4.99-20.86) | *0.001 |

| Alcohol  drinking habit | No | 82 (3.5) | 2243 (96.5) | 1 |  | 1 |  |
| --- | --- | --- | --- | --- | --- | --- | --- |
| Distance from  home to HCF | ≤ 40 km | 70 (6.9) | 942 (93.1) | 1 |  | 1 |  |
|  | > 40 km | 80 (5.2) | 1456 (94.8) | 0.74 (0.53-1.03) | 0.074 | 2.35 (0.78-10.57) | 0.691 |
| Type of health facility visited | Health post | 32 (5.7) | 534 (94.3) | 0.12 (0.03-0.50) | 0.004 | 0.02 (0.01-0.12) | *0.001 |
|  | Health center | 45 (6.1) | 687 (93.9) | 0.13 (0.03-0.54) | 0.005 | 0.03 (0.06-0.15) | *0.001 |
|  | Gov’t hospital | 43 (5.4) | 754 (94.6) | 0.11 (0.03-0.47) | 0.003 | 0.03 (0.07-0.17) | *0.001 |
|  | Private hospital/clinics | 27 (6.1) | 417 (93.9) | 0.13 (0.03-0.54) | 0.005 | 0.03 (0.07-0.18) | *0.001 |
|  | Others | 3 (33.3) | 6 (66.7) | 1 |  | 1 |  |
| Actions taken for the first appearance of symptoms | Self-medication | 39 (5.0) | 742 (95.0) | 0.59 (0.35-0.99) | 0.047 | 0.83 (0.36-1.90) | 0.653 |
|  | Traditional medicine | 47 (6.8) | 648 (93.2) | 0.82 (0.49-1.35) | 0.436 | 1.19 (0.62-2.32) | 0.598 |
|  | Holly water | 18 (3.9) | 442 (96.1) | 0.46 (0.25-0.85) | 0.014 | 0.71 (0.32-1.57) | 0.397 |
|  | Consult HCWs locally | 20 (6.8) | 272 (93.2) | 0.83 (0.45-1.52) | 0.550 | 1.70 (0.51-5.64) | 0.385 |
|  | Consult HCWs at HCF | 26 (8.1) | 294 (91.9) | 1 |  | 1 |  |
| **Total** | | **150 (5.9)** | **2398 (94.1)** |  |  |  |  |

268

269

270

271

272

AOR: Adjusted odds ratio; COR: Crude odds ratio; HCW: Health care workers; HCF: Health care facility

# 273 4. Discussion

274 The overall prevalence of *M. tuberculosis* among pulmonary tuberculosis (PTB)-suspected patients (5.9%;
275 150/2,548) is on the lower end of recent hospital-based Ethiopian findings. A multi-year GeneXpert
276 program data reported 10.5% positivity among 17,615 presumptive tuberculosis patients from 2015 to 2021
277 [14]. A 2024 trend analysis from Northwest Ethiopia found 11.7% positivity in 3,696 presumptive cases,
278 again higher than the current study [15]. Other single-site studies likewise report higher yields: 12.7% in a
279 2023 cross-sectional study using GeneXpert, 17.2% in 2025 analysis of 273 presumptive patients, and
280 26.8% in a smaller facility-based series [10, 16, 17].
281 The magnitude of *M. tuberculosis* in the current study falls within the range reported in the Ethiopian
282 population surveys, which estimate bacteriologically-confirmed PTB prevalence near 147 per 100,000
283 population, although much higher rates have also been seen depending on case definition and diagnostic
284 method. Localized studies in hotspot regions and high-risk occupational groups, such as miners, report
285 notably higher prevalence, exceeding 7% or more in specific subpopulations [18]. Demographically, the
286 finding of marginally higher prevalence in males aligns with national and regional data, where men
287 consistently show elevated notification and incidence rates, with male-to-female ratios up to 1.4. The
288 observed age gradient-with peak PTB prevalence among those at or above 66 years-matches the pattern
289 seen across Ethiopia and Sub-Saharan Africa: older adults face higher PTB risk, while young adults
290 constitute the majority of notified cases due to larger population size [19].
291 The urban *M. tuberculosis* prevalence rate (7.0%) versus rural (5.2%) slightly contrasts with some earlier
292 Ethiopian surveys, which show greater PTB notification in rural settings, largely a reflection of healthcare
293 access and reporting; however, escalating urbanization and overcrowding are increasingly driving for
294 higher urban PTB rates. Inter-site differences (Gondar, Bahir Dar, Debre Markos) reflect spatial clustering
295 documented in the Amhara Regional State and other regions, where migration and local socioeconomic
296 factors influence local PTB rates [20]. Education-wise, higher PTB prevalence among illiterate individuals
297 is echoed in a Tanzanian study, where both extremes of educational attainment carried higher risk-
298 potentially due to different exposure patterns, awareness, or mobility. Occupational differences are
299 consistent with regional literature: merchant, government employees, and farmers often show higher
300 prevalence, while students and daily laborers tend to record the lowest rates-mirroring high PTB risks for
301 those exposed to crowded or migratory work environments [21].
302 The PTB prevalence was highest among
303 individuals with higher income (>6,001 ETB) and among divorced and widowed participants, indicate that
304 the risk of PTB does not necessarily decrease with increasing income. This may be linked to factors such
305 as urban residence and occupational exposures. Likewise, the elevated prevalence among divorced and
306 widowed individuals has been documented elsewhere and may reflect social vulnerability, reduced support
307 networks, and related health challenges [22].
308 The magnitude of *M. tuberculosis* with respect to educational attainment was 28% among illiterate and
309 only 18.8% having college-level education or higher, parallels report from other Ethiopian settings: for
310 example, identified low education as a major associated factor for PTB clustering, particularly among
311 internal migrants and in hotspots with high rural population density. Occupational patterns show a similar
312 picture elsewhere: farmers and day laborers carry greater PTB vulnerability due to socioeconomic
313 challenges, frequent migration, and crowded living environments. Income distributions in PTB studies
314 across Ethiopia and Kenya typically reveal most patients earning under local median incomes, emphasizing
315 the links between poverty and PTB risk [23]. A comparative study in Kenya and other parts of Sub-Saharan
316 Africa also highlights a concentration of PTB cases among young adults, males, and those of low
317 socioeconomic and educational status. The clustering of PTB in densely populated, lower-income, and rural
318 areas remains a consistent theme, with migration and poor living conditions frequently cited as amplifying
319 factors [24].
320 Among the 150 *M. tuberculosis* positive participants tested with the GeneXpert MTB/RIF assay, rifampicin
321 resistance (RR) was detected in 12.7% of cases. Rifampicin resistance was slightly higher among females
322 (14.8%) compared to males (10.5%). Age-wise, the highest resistance was in the 31-40 years group
323 (21.4%). Urban residents had a higher rifampicin resistance rate (18.6%) than rural residents (7.5%). The
324 rifampicin resistance rate was relatively consistent across study sites: Gondar (14%), Bahir Dar (12%), and
325 Debre Markos (12%). A study in Pakistan among pediatric PTB patients found 4.5% rifampicin-resistant
326 *M. tuberculosis*, a rate lower than the 12.7% reported here, reflecting possible differences in population
327 and settings [25]. A study in Southwest Ethiopia found rifampicin resistance at 3.4%, which is lower than
328 the current study, but was noted to be higher in previously treated cases, aligning with the increased
329 resistance seen in certain age and urban groups in this data [26]. Another Ethiopian study reported
330 rifampicin resistance at 9.8%, close to but slightly lower than the 12.7% seen, with similar observations of
331 higher resistance among females and HIV status [27]. A study reviewing a larger population showed a
332 declining trend, but still reported notable resistance levels in both adults and children, with 8.3% rifampicin
333 resistance among adults and 7.2% in children, somewhat lower but comparable to the current findings [28].
334 Socio-economic determinants such as low education and poor knowledge contribute to delayed diagnosis
335 and poor adherence, exacerbating rifampicin resistance risks, particularly in urban and certain occupational
336 groups with higher social mixing. The influence of income and household size on resistance is less
337 frequently documented explicitly but is consistent with observations of complex risk factors influencing
338 PTB epidemiology and drug resistance [15, 29]. Interventions targeting education, treatment adherence,
339 and socioeconomic barriers remain crucial in controlling rifampicin-resistant *M. tuberculosis* [27, 30]. A
340 meta-analyses and pooled molecular-surveillance studies for Ethiopia report pooled rifampicin resistance
341 (RR) generally in the 7-9% range, so our study 12.7% is noticeably above those pooled averages,
342 suggesting either local hot-spots or sample differences [6, 31].
343 Rifampicin resistance was highest among those with Grade 1-4 education (20.0%), followed by grade 5-8
344 (16.7%) and grade 9-12 (14.8%). This aligns with broader patterns showing elevated RR or MDR-TB
345 among populations with low educational attainment, though explicit stratification by educational level is
346 not frequently reported. For instance, one study from Northwest Ethiopia found that poorest and least
347 educated adults had disproportionately higher MDR-TB rates, underscoring the vulnerability of the poorly
348 educated to drug-resistant disease [32]. Data on occupation, merchants (21.9%) and government employees
349 (15.2%) showed with notably higher resistance compared to farmers (6.6%), and none among students. In
350 contrast, a cross-sectional analysis from West Armachiho and Metema districts in Northwest Ethiopia
351 reported no significant association between occupation and MDR-TB after adjustment; although raw
352 numbers showed some MDR-TB among farmers/day laborers, the regression found occupation-including
353 being a farmer-was not a predictor once other factors were accounted [33]. In the current study, a relatively
354 high proportion of indeterminate results (7.3%) in *M. tuberculosis* rifampicin susceptibility testing,
355 particularly when using the GeneXpert MTB/RIF assay, may be attributed to several well-recognized
356 technical and biological factors. These include low bacillary load in clinical specimens, suboptimal sample
357 quality, the presence of PCR inhibitors, instrument- or cartridge-related issues, and mutations occurring
358 outside the rifampicin resistance–determining region (RRDR).
359 In this study, the prevalence of PTB among suspected cases was influenced by several socio-demographic
360 characteristics. Male participants showed a slightly higher PTB prevalence (6.4%) compared to females
361 (5.4%), though the association was not statistically significant, aligning with studies from Ethiopia,
362 Uganda, and India that consistently reported higher PTB infection among men, likely due to greater
363 occupational exposure and health-seeking behavior differences [34-36]. In the multivariate model, age,
364 residence and occupation were independently associated with PTB status. Older age categories showed
365 significantly lower odds of PTB compared with the 8-15-year age group (AOR: 0.06-0.14; *p*= 0.001). In
366 contrast, a study reported that older age showed higher odds for PTB, highlighting a higher PTB risk in
367 adults than children and adolescents in sub-Saharan Africa [37]. Further, these findings were inconsistent
368 from other studies in Ethiopia and low- and middle-income countries (LMICs), where low education is
369 frequently associated with higher PTB risk due to its correlation with socioeconomic status and health
370 literacy [38, 39]. Students by occupation remained lower odds for PTB (AOR: 0.03; 95%CI: 0.01-0.11)
371 compared to government employees. This is contrary to study in Ethiopia which showed type of occupation
372 was associated with increased odds of active PTB disease. Additionally, broader literature on PTB and
373 socioeconomic status demonstrates that low socioeconomic status is consistently associated with higher
374 PTB incidence and vulnerability [4, 40].
375 Chest pain was a less likely indicators of PTB, with adjusted odds ratio (AOR: 0.25; 95%CI: 0.05-0.87; *p*=
376 0.031), indicating that PTB suspects presenting chest pain are less likely to be PTB-positive. However,
377 cough and fever are common in PTB, were not strongly associated in this analysis, echoing the diagnostic
378 challenges reported in Ethiopian health facilities. Weight loss and hemoptysis did not reach statistical
379 significance in multivariate analysis, which matches observations that such symptoms are less sensitive for
380 PTB diagnosis in generalized screening but may still indicate advanced disease [41].
381 Previous antibiotic use and alcohol drinking were significantly associated with PTB, whereas HIV status,
382 diabetes, smoking, family history of TB, distance to health facilities, and first health-seeking actions were
383 not significant after adjustment. This finding is consistent with a study from Debre Markos Referral
384 Hospital, which reported a significant association between previous antibiotic use and PTB [42]. Similarly,
385 studies from Sekota town identified alcohol consumption as a significant factor, although they also reported
386 HIV seropositivity and contact with PTB patients as important predictors, unlike the present study [39, 43].
387 In contrast, studies from Adigrat General Hospital demonstrated that HIV infection and age were
388 significantly associated with rifampicin resistance, differing from the current non-significant association
389 with HIV [44, 45]. Although cigarette smoking was significant in the bivariate analysis, it lost significance
390 in the multivariate model, despite strong evidence from other studies showing smoking as an independent
391 risk factor for PTB progression and poor outcomes [46]. Alcohol drinking remained significant in both
392 models, supporting previous Ethiopian and international evidence linking alcohol use with increased PTB
393 susceptibility and adverse outcomes [47]. Furthermore, source of drinking water and distance to health
394 facilities were not significant, diverging from studies that report these factors as important for other
395 infectious diseases but less consistently for PTB unless combined with broader social determinants [41, 45,
396 48].
397 Differences in prevalence across studies usually reflect: (i) case mix and inclusion criteria (symptom
398 duration thresholds, inclusion of extrapulmonary specimens, proportion of retreatment cases-retreatment
399 consistently shows higher positivity); (ii) diagnostic approach and quality (GeneXpert vs. smear/culture,
400 cartridge performance, error/invalid rates, and laboratory quality systems); (iii) epidemiologic context
401 (local TB/HIV burden, urban-rural composition, crowding, and contact patterns); and (iv) time
402 period/program effects (expansion of screening, earlier care-seeking, and secular declines). For example,
403 retreatment patients had substantially higher positivity than new cases in national program data, while
404 program reports also document cartridge wastage/error that can influence measurable yield; both factors
405 can push site-level prevalence up or down [14, 49]. In short, the lower estimate likely reflects a combination
406 of broader catchment with many low-risk presumptive, fewer retreatment cases, and improved case-finding
407 relative to earlier single-site studies.

# 408 Limitations of the study

409 The use of the GeneXpert MTB/RIF assay, detects only rifampicin-resistant and not resistance to other
410 first-line or second-line anti-TB drugs. The study included patients from selected comprehensive
411 specialized hospitals, which may not represent the broader population in the Amhara National Regional
412 State, particularly rural areas or those without access to advanced health facilities. Information on socio-
413 demographic and clinical factors was collected using self-reported questionnaires, which could be subject
414 to recall bias and social desirability bias.

# 415 5. Conclusion and recommendation

416 The prevalence of *M. tuberculosis* among suspected cases was 5.9%, with higher rates observed among
417 older age groups and urban residents. Rifampicin resistance was observed in 12.7% of isolates. Pulmonary
418 tuberculosis was significantly associated with prior antibiotic use and alcohol consumption. Strengthening
419 early *M. tuberculosis* detection and routine rifampicin resistance testing is crucial, especially among high-
420 risk groups. Interventions should promote appropriate antibiotic use, and reduce alcohol consumption.
421 Focused health education and ongoing surveillance of drug-resistant *M. tuberculosis* are needed to limit
422 transmission and improve PTB control in the region.

423

424

# 425 Abbreviations

426 AMR: Antimicrobial resistance, DMCSH: Debre Markos Comprehensive Specialized Hospital, FHCSH:
427 Felege-Hiwot Comprehensive Specialized Hospital, MDR-TB: Multidrug-resistant tuberculosis, PTB:
428 Pulmonary tuberculosis, UoGCSH: University of Gondar Comprehensive Specialized Hospital

# 429 Acknowledgments

430 We would like to sincerely thank Debre Markos, Felege-Hiwot and University of Gondar Comprehensive
431 Specialized Hospitals administration and staffs for allowing us to conduct the research in the hospitals. We
432 acknowledge the study participants for their participation without them the research would not be a reality.

# 433 Availability of data and material

434 All relevant data are within the manuscript and its supporting information files.

# 435 Competing interest

436 The authors declare that they have no competing interests.

# 437 Funding

438 The authors received no specific funding for this work.

# 439 Authors’ contribution

440 TD: Conceptualization, Methodology, Investigation, Data curation, Project administration, Formal
441 analysis, Resources, Software, Writing- original draft, and Writing- review and editing
442 FM: Conceptualization, Methodology, Supervision, Project administration, Validation, and Writing- review
443 and editing
444 BT: Methodology, Supervision, Project administration, Validation, and Writing- review and editing
445 WH: Methodology, Supervision, Project administration, Validation and Writing- review and editing
446 BG: Conceptualization, Methodology, Investigation, Supervision, Project administration, Validation, and
447 Writing- review and editing

# 448 References

449 1.	WHO. Global tuberculosis report 2023. Geneva: World Health Organization; 2023. Licence: CC
450 BY-NC-SA 3.0 IGO.
451 2.	Salari N, Kanjoori AH, Hosseinian-Far A, Hasheminezhad R, Mansouri K, Mohammadi M. Global
452 prevalence of drug-resistant tuberculosis: a systematic review and meta-analysis. *Infect Dis Poverty*.
453 2023;12(1):57.
454 3.	FMoH. Ethiopia national TB program annual performance report. Ministry of Health, Ethiopia.
455 2022.
456 4.	Shimeles E, Enquselassie F, Aseffa A, Tilahun M, Mekonen A, Wondimagegn G, *et al*. Risk factors
457 for tuberculosis: A case-control study in Addis Ababa, Ethiopia. *PloS One*. 2019;14(4):e0214235.
458 5.	Shiferaw MB, Sinishaw MA, Amare D, Alem G, Asefa D, Klinkenberg E. Prevalence of active
459 tuberculosis disease among healthcare workers and support staff in healthcare settings of the Amhara
460 region, Ethiopia. *PloS One*. 2021;16(6):e0253177.
461 6.	Birhanu MY, Bekele GM, Jemberie SS. Molecular detection of rifampicin-resistant *Mycobacterium*
462 *tuberculosis* by polymerase chain reaction in Ethiopia: a systematic review and meta-analysis. *Front Med*.
463 2024;11:1319845.
464 7.	WHO. Automated Real-Time Nucleic Acid Amplification Technology for Rapid and Simultaneous
465 Detection of Tuberculosis and Rifampicin Resistance: Xpert MTB/RIF Assay for the Diagnosis of
466 Pulmonary and Extrapulmonary TB in Adults and Children, 2013. Geneva: Copyright © World Health
467 Organization 2013.
468 8.	Boehme CC, Nabeta P, Hillemann D, Nicol MP, Shenai S, Krapp F, *et al*. Rapid molecular detection
469 of tuberculosis and rifampin resistance. *N Engl J Med*. 2010;363(11):1005-15.
470 9.	Badgeba A, Shimbre MS, Gebremichael MA, Bogale B, Berhanu M, Abdulkadir H. Determinants
471 of Multidrug-Resistant *Mycobacterium tuberculosis* Infection: A Multicenter Study from Southern
472 Ethiopia. *Infect Drug Resist*. 2022;15:3523-35.
473 10.	Asmare Z, Assefa A, Belay A, Mulie A, Aschil D, Gashaw Y. Prevalence and factors associated
474 with TB among presumptive TB patients in war-affected areas of North Wollo, Ethiopia. *JAC Antimicrob*
475 *Resist*. 2025;7(1):dlae206.
476 11.	Girum T, Muktar E, Lentiro K, Wondiye H, Shewangizaw M. Epidemiology of multidrug-resistant
477 tuberculosis (MDR-TB) in Ethiopia: a systematic review and meta-analysis of the prevalence, determinants
478 and treatment outcome. *Trop Dis Travel Med Vaccines*. 2018;4(1):5.
479 12.	WHO. Definitions and reporting framework for tuberculosis-2013 revision. Geneva: WHO. 2013.
480 13.	Ramaswamy S, Musser JM. Molecular genetic basis of antimicrobial agent resistance in
481 *Mycobacterium tuberculosis*: 1998 update. *Tubercle Lung Dis*. 1998;79(1):3-29.
482 14.	Biset S, Teferi M, Alamirew H, Birhanu B, Dessie A, Aschale A, *et al*. Trends of *Mycobacterium*
483 *tuberculosis* and Rifampicin resistance in Northwest Ethiopia: Xpert® MTB/RIF assay results from 2015
484 to 2021. *BMC Infect Dis*. 2024;24(1):238.
485 15.	Gebremariam G, Kiros M, Hagos S, Hadush H, Gebremichael A, Gebrekirstos G, *et al*. Trend of
486 pulmonary tuberculosis and rifampicin-resistance among tuberculosis presumptive patients in Central
487 Tigray, Ethiopia; 2018 -2023: a six-year retrospective study. *Trop Dis Travel Med Vaccines*. 2024;10(1):15.
488 16.	Wubu B, Jemal M, Million Y, Gizachew M. Pulmonary tuberculosis and multidrug-resistant
489 *Mycobacterium tuberculosis* in northwestern Ethiopia: a hospital-based cross-sectional study among
490 presumptive pulmonary tuberculosis patients. *Front Med*. 2023;10:1266780.
491 17.	Andarge DB, Anticho TL, Jara GM, Ali MM. Prevalence of *Mycobacterium tuberculosis* infection
492 and rifampicin resistance among presumptive tuberculosis cases visiting tuberculosis clinic of Adare
493 General Hospital, Southern Ethiopia. *SAGE Open Med*. 2021;9:20503121211045541.
494 18.	Banti AB, Winje BA, Hinderaker SG, Heldal E, Abebe M, Dangisso MH, *et al*. Prevalence and
495 incidence of symptomatic pulmonary tuberculosis based on repeated population screening in a district in
496 Ethiopia: a prospective cohort study. *BMJ Open*. 2023;13(7):e070594.
497 19.	Kebede AH, Alebachew Z, Tsegaye F, Lemma E, Abebe A, Agonafir M, *et al*. The first population-
498 based national tuberculosis prevalence survey in Ethiopia, 2010-2011. *Int J Tuberc Lung Dis*.
499 2014;18(6):635-9.
500 20.	Getahun M, Beyene D, Mollalign H, Diriba G, Tesfaye E, Yenew B, *et al*. Population structure and
501 spatial distribution of *Mycobacterium tuberculosis* in Ethiopia. *Sci Rep*. 2024;14(1):10455.
502 21.	Mbuya AW, Mboya IB, Semvua HH, Mamuya SH, Msuya SE. Prevalence and factors associated
503 with tuberculosis among the mining communities in Mererani, Tanzania. *PloS One*. 2023;18(3):e0280396.
504 22.	Gashaw M, Abtew D, Addis Z. Prevalence and Antimicrobial Susceptibility Pattern of Bacteria
505 Isolated from Mobile Phones of Health Care Professionals Working in Gondar Town Health Centers. *Int*
506 *Sch Res Notices*. 2014;2014(1):205074.
507 23.	Heaton B. Prevalence of Tuberculosis in Sub-Saharan Africa. Ballard Brief. March 2022.
508 24.	Tola HH, Karimi M, Yekaninejad MS. Effects of sociodemographic characteristics and patients'
509 health beliefs on tuberculosis treatment adherence in Ethiopia: a structural equation modelling approach.
510 *Infect Dis Poverty*. 2017;6(1):167.
511 25.	Jameel R, Mehdi N, Majeed N, Zafar A, Tahir A, Aroob I. Prevalence and Diagnosis of Rifampicin-
512 Resistant Mycobacterium Tuberculosis using the GeneXpert MTB/RIF Assay at a Tertiary Care Children's
513 Hospital: Prevalence and Diagnosis of Rifampicin-Resistant *Mycobacterium tuberculosis. PJHS*.
514 2024;5(07):110-4.
515 26.	Admassu W, Ayelign B, Abebe G, Tadesse M. Detection of *Mycobacterium tuberculosis* and
516 rifampicin resistance by Xpert® MTB/RIF assay among presumptive tuberculosis cases at Jimma
517 University Medical Center, Southwest Ethiopia. *PloS One*. 2022;17(1):e0262929.
518 27.	Araya S, Negesso AE, Tamir Z. Rifampicin-Resistant *Mycobacterium tuberculosis* Among Patients
519 with Presumptive Tuberculosis in Addis Ababa, Ethiopia. *Infect Drug Resist*. 2020;13:3451-9.
520 28.	Wasihun AG, Hailu GG, Dejene TA. Prevalence of *Mycobacterium tuberculosis* (Rifampicin-
521 Resistant MTB) and Associated Risk Actors Among Pulmonary Presumptive TB Patients in Eastern
522 Amhara, Ethiopia: 2015-2019. *Infect Dis Ther*. 2021;10(3):1299-308.
523 29.	Cadmus S, Akinseye VO, Olagunju T, Makolo A, Falodun O, Lawal O, *et al*. Risk factors for
524 rifampicin resistance tuberculosis among patients attending Directly Observed Treatments Centres in
525 Southwestern Nigeria. *Pan Afr Med J*. 2024;49:87.
526 30.	Arega B, Menbere F, Getachew Y. Prevalence of rifampicin resistant *Mycobacterium tuberculosis*
527 among presumptive tuberculosis patients in selected governmental hospitals in Addis Ababa, Ethiopia.
528 *BMC Infect Dis*. 2019;19(1):307.
529 31.	Demelash M, Nibret E, Hailegebriel T, Minichil Z, Mekonnen D. Prevalence of rifampicin resistant
530 pulmonary tuberculosis using geneXpert assay in Ethiopia, a systematic review and meta-analysis. *Heliyon*.
531 2023;9(9):e19554.
532 32.	Zereabruk K, Kahsay T, Teklemichael H, Aberhe W, Hailay A, Mebrahtom G, *et al.* Determinants
533 of multidrug-resistant tuberculosis among adults undergoing treatment for tuberculosis in Tigray Region,
534 Ethiopia: a case-control study. *BMJ Open Respir Res*. 2024;11(1).
535 33.	Mekonnen F, Tessema B, Moges F, Gelaw A, Eshetie S, Kumera G. Multidrug resistant
536 tuberculosis: prevalence and risk factors in districts of metema and west armachiho, Northwest Ethiopia.
537 *BMC Infect Dis*. 2015;15:461.
538 34.	Ramos JM, Comeche B, Tesfamariam A, Reyes F, Tiziano G, Balcha S, *et al*. Sex differences and
539 HIV status of tuberculosis in adults at a rural hospital in southern Ethiopia: an 18-year retrospective cross-
540 sectional study. *Afr Health Sci*. 2020;20(2):605-14.
541 35.	Sekandi JN, Quach T, Olum R, Nakkonde D, Farist L, Obiekwe R, *et al.* Stigma and associated sex
542 disparities among patients with tuberculosis in Uganda: a cross-sectional study. *Ther Adv Infect Dis*.
543 2024;11:20499361241305517.
544 36.	Roy M. Sociodemographic Determinants of Tuberculosis Burden in India. *Amrita J Med*.
545 2021;17:45.
546 37.	Horton KC, MacPherson P, Houben RM, White RG, Corbett EL. Sex Differences in Tuberculosis
547 Burden and Notifications in Low- and Middle-Income Countries: A Systematic Review and Meta-analysis.
548 *PLoS Med*. 2016;13(9):e1002119.
549 38.	Mohammed H, Oljira L, Roba KT, Ngadaya E, Manyazewal T, Ajeme T, *et al*. Tuberculosis
550 Prevalence and Predictors Among Health Care-Seeking People Screened for Cough of Any Duration in
551 Ethiopia: A Multicenter Cross-Sectional Study. *Front Public Health*. 2022;Volume 9 - 2021.
552 39.	Mengistu G, Tekeste Z, Mehabie D, Tesfaye S, Alelign A. Prevalence, risk factors, and rifampicin
553 resistance pattern of *Mycobacterium tuberculosis* in Sekota town, Northwest Ethiopia. *Parasite Epidemiol*
554 *Control*. 2026;32:e00471.
555 40.	Wang Q, Guo L, Wang J, Zhang L, Zhu W, Yuan Y, *et al*. Spatial distribution of tuberculosis and its
556 socioeconomic influencing factors in mainland China 2013-2016. *Trop Med Int Health*. 2019;24(9):1104-13.
557 41.	Mohammed H, Oljira L, Teji Roba K, Ngadaya E, Mehari R, Manyazewal T, *et al*. Who to Involve
558 and Where to Start Integrating Tuberculosis Screening into Routine Healthcare Services: Positive Cough
559 of Any Duration as the First Step for Screening Tuberculosis in Ethiopia. *Risk Manag Healthc Policy*.
560 2021;14:4749-56.
561 42.	Mulu W, Abera B, Yimer M, Hailu T, Ayele H, Abate D. Rifampicin-resistance pattern of
562 *Mycobacterium tuberculosis* and associated factors among presumptive tuberculosis patients referred to
563 Debre Markos Referral Hospital, Ethiopia: a cross-sectional study. *BMC Res Notes*. 2017;10(1):8.
564 43.	Endalew G, Beyene MB, Kassie A, Wassie GT. Incidence and Risk Factors of Tuberculosis among
565 Children Receiving Antiretroviral Therapy in Northwest, Ethiopia. *Int J Public Health*. 2025;70:1607892.
566 44.	Abay GK, Abraha BH. Trends of *Mycobacterium tuberculosis* and rifampicin resistance in Adigrat
567 General Hospital, Eastern zone of Tigrai, North Ethiopia. *Trop Dis Travel Med Vaccines*. 2020;6:14.
568 45.	Diriba K, Awulachew E. Associated risk factor of tuberculosis infection among adult patients in
569 Gedeo Zone, Southern Ethiopia. *SAGE Open Med*. 2022;10:20503121221086725.
570 46.	Alemu YM, Awoke W, Wilder-Smith A. Determinants for tuberculosis in HIV-infected adults in
571 Northwest Ethiopia: a multicentre case-control study. *BMJ Open*. 2016;6(4):e009058.
572 47.	Fenta A, Demeke G, Bitew A, Kebede D, Hailu T. Prevalence and Associated Factors of TB Co-
573 Morbidity Among HIV Sero-Positive Individuals in Shegaw Motta District Hospital, Ethiopia. *Int J Gen*
574 *Med*. 2020;13:1529-36.
575 48.	Abaynew Y, Ali A, Taye G. Social determinants of tuberculosis in Addis Ababa, Ethiopia: a
576 qualitative study. *Sci Rep*. 2025;15(1):15961.
577 49.	Ramachandra V, Brammacharry U, Muralidhar A, Muthukumar A, Mani R, Muthaiah M, *et al*.
578 Assess the Diagnostic Accuracy of GeneXpert to Detect *Mycobacterium tuberculosis* and Rifampicin-
579 Resistant Tuberculosis among Presumptive Tuberculosis and Presumptive Drug Resistant Tuberculosis
580 Patients. *Microbiol Res*. 2024;15(1):91-108.

# 581 Supporting Information

582 **S 1 data:** Raw data set in excel

Supporting Information

[Click here to access/download](https://www2.cloud.editorialmanager.com/pone/download.aspx?id=44212887&guid=16641f05-7c71-43c7-ac00-5a968bfcba36&scheme=1)

[**Supporting Information** S1 Data.xlsx](https://www2.cloud.editorialmanager.com/pone/download.aspx?id=44212887&guid=16641f05-7c71-43c7-ac00-5a968bfcba36&scheme=1)
