# Supplementary material for: Rifampicin resistance and associated factors of Mycobacterium tuberculosis among pulmonary tuberculosis-suspected patients in the Amhara National Regional State Comprehensive Specialized Hospitals, Ethiopia
Source: PLoS One. 2026 Jun 26;21(6):e0351964. doi: 10.1371/journal.pone.0351964 (PMC13309019; doi:10.1371/journal.pone.0351964)
Supplement: Attachment — Submitted filename: Comments on Rifampicin resistance among MTB-2026.docx [file pone.0351964.s005.docx]

**General Comments**

The authors are appreciated for addressing an important public health issue, particularly in developing countries such as Ethiopia. The manuscript is generally well organized; however, several aspects require revision to enhance its scientific rigor, clarity, and overall quality. The study aimed *“to determine the prevalence of rifampicin-resistant Mycobacterium tuberculosis and associated factors among tuberculosis-suspected patients at Amhara National Regional State Comprehensive Specialized Hospitals, Northwest Ethiopia.”*

The authors reported an overall prevalence of *M. tuberculosis* of 150/2548 (5.9%), with rifampicin resistance detected in 19/150 (12.7%) of confirmed cases. Several factors, including age, residence, occupation, income, marital status, previous antibiotic use, and alcohol consumption, were found to be independently associated with *M. tuberculosis* infection. Despite these contributions, the manuscript can be improved in the following areas:

**ABSTRACT**

**Background**

- Please include brief background information on rifampicin resistance to better contextualize the study.

**Objective**

- It is preferable to replace the phrase *“to determine”* with *“to assess”* for improved academic tone.

**Conclusion**

- The conclusion requires revision to ensure consistency with the stated objective and results.
- Statements such as *“the prevalence remains significant”* should be supported by a clear baseline or comparison (e.g., previous studies in the study area or national estimates). Consider using terms like *high* or *low* with appropriate references.
- Key statistically significant factors identified in the results (e.g., age, residence, occupation, income, marital status) should also be reflected in the conclusion and recommendations.

**MATERIALS AND METHODS**

**Study Population, Inclusion, and Exclusion Criteria**

- Provide justification for excluding patients aged <8 years and clarify the rationale for including participants aged ≥8 years.

**Sample Size Determination and Sampling Technique**

- The sample size calculation is not clearly described. Please specify how the total sample size and the 150 *M. tuberculosis* cases were obtained.
- The statement *“50 M. tuberculosis-positive participants were included from each health facility”* needs reconsideration. A proportional allocation based on the source population of each facility would be more appropriate than equal allocation.
- Revise this section to include:
  - A clear sample size calculation formula and assumptions
  - Proportional allocation of participants across study sites

**Data Collection Tool**

- Indicate where the pre-test of the questionnaire was conducted and the number of participants involved.

**RESULTS**

**Statistical Analysis**

- This section requires substantial revision for clarity and methodological correctness.

1. **Confidence Intervals**
   - Please include 95% confidence intervals (CIs) in Table 2 to facilitate comparison with other studies in the discussion section.
2. **Regression Analysis Approach**
   - Clarify how independent variables were selected for bivariable and multivariable logistic regression.
   - It is methodologically inappropriate to analyze sociodemographic variables separately from clinical and behavioral factors in multivariable models.
   - Variables with p-value ≤0.25 in the bivariable analysis should be entered together into a single multivariable logistic regression model.
   - Tables 3 and 4 should ideally be merged into one comprehensive table. If separated for clarity, ensure that all variables are included in a unified multivariable model.
3. **Table 3 and Description**
   - There are inconsistencies between the table and its description. For example:
     - The reference category for age is listed as 8–15 years in the table, whereas the text compares ≥66 years with 16–30 years.
     - This inconsistency must be corrected.
   - The finding that participants with income <3000 birr have lower odds of infection compared to those earning >6000 birr (AOR: 0.53; 95% CI: 0.29–0.94) requires careful interpretation. Please verify the reference category and ensure the correctness of the statistical analysis.
4. **Table 4**
   - For the variable *“type of health facility visited,”* specify the categories included under *“others”* in a footnote, or revise the reference category for clarity.

Overall, addressing these issues will substantially improve the methodological soundness and clarity of the manuscript.
