# Supplementary material for: Rifampicin resistance and associated factors of Mycobacterium tuberculosis among pulmonary tuberculosis-suspected patients in the Amhara National Regional State Comprehensive Specialized Hospitals, Ethiopia
Source: PLoS One. 2026 Jun 26;21(6):e0351964. doi: 10.1371/journal.pone.0351964 (PMC13309019; doi:10.1371/journal.pone.0351964)
Supplement: Attachment — Submitted filename: Regarding key words please add.docx [file pone.0351964.s008.docx]

1. Regarding key words please add:

- Tuberculosis, Amhara Region

2. In table 1: the 95% CI level for unemployed is beyond the range 3(4.3, 7.3). Therefore, please remove 95% CI from the table if possible.
3. In table 3: use the reference “rural” not urban. Because your focus is on prevalence. Also for Table 5.
4. In table 3: illiterate is not logical word. Use “no formal education”
