# Supplementary material for: Rifampicin resistance and associated factors of Mycobacterium tuberculosis among pulmonary tuberculosis-suspected patients in the Amhara National Regional State Comprehensive Specialized Hospitals, Ethiopia
Source: PLoS One. 2026 Jun 26;21(6):e0351964. doi: 10.1371/journal.pone.0351964 (PMC13309019; doi:10.1371/journal.pone.0351964)
Supplement: Attachment — Submitted filename: Response_to_Reviewers_auresp_2.docx [file pone.0351964.s010.docx]

**Response to reviewers**

**Dear Editor- in-Chief,**

**PLoS One**

This is a revised versions of the manuscript entitled: “**Rifampicin resistance and associated factors of *Mycobacterium tuberculosis* among pulmonary tuberculosis-suspected patients in** **the Amhara National Regional State Comprehensive Specialized Hospitals, Ethiopia**” to be considered for publication in “**PLoS One**”.

**Dear Academic Editor,**

We sincerely thank you for your decision and for the valuable detailed comments provided, which have been extremely helpful in improving our manuscript. We are also very grateful to the reviewers for their detailed, thoughtful, constructive comments and questions. Please find the attached revised version of the manuscript, which has been carefully updated in accordance with the comments and suggestions from the editor and reviewers. All comments have been carefully considered and incorporated into the manuscript. In addition, the entire manuscript has been thoroughly proofread to improve clarity, grammar, and overall presentation.

- **Journal requirements:** If the reviewer comments include a recommendation to cite specific previously published works, please review and evaluate these publications to determine whether they are relevant and should be cited. There is no requirement to cite these works unless the editor has indicated otherwise.
- **Author response:** The suggested articles were carefully reviewed and evaluated for their relevance to the current study. Citations were incorporated where they were directly relevant and strengthened the scientific context and discussion of the manuscript. However, references were not included solely based on reviewer recommendation, in accordance with the journal’s citation policy.

**Responses to Academic Editor**

1. **Editor**: Several sections contain unnecessary detail, making the Results section lengthy and difficult to follow.

**Author response**: Thank you for this valuable comment. We agree that several parts of the Results section contained excessive detail, which affected the overall clarity and readability of the manuscript. Accordingly, we carefully revised and condensed the Results section by removing redundant information and presenting the findings more concisely to improve the logical flow and readability of the manuscript.

2. **Editor**: Important findings should be highlighted concisely, while detailed distributions can remain in the tables.

**Author response**: We agree that the major findings should be emphasized concisely in the text, while detailed distributions are more appropriately presented in the tables. Accordingly, we revised the manuscript carefully.

3. **Editor**: Consistent use of abbreviations, numbering formats, terminology, and statistical reporting throughout the manuscript is recommended.

**Author response**: We appreciate the editor’s comment. The manuscript was carefully proofread and revised to ensure consistent use of abbreviations, numbering formats, terminology, and statistical reporting throughout the document.

4. **Editor**: The Methods section should be further summarized by removing unnecessary details and excessive subheadings to improve readability and flow.

**Author response**: Thank you for this valuable comment. We agree that the Methods section contained excessive details and multiple subheadings that affected the overall readability and flow of the manuscript. Accordingly, we have revised and summarized the Methods section by removing unnecessary details, merging related subsections, and simplifying the presentation to improve clarity and readability.

**Responses to Reviewer #5**

Thank you dear reviewer, for your time, comments and questions to us for improvement of our manuscript. We accepted comments and incorporated to the paper and we gave some responses to your comments and questions below.

1. **Reviewer 5**: Regarding key words please add: Tuberculosis, Amhara Region

**Author response**: Thank you for the comment. I accept the suggestion and have incorporated the keywords “Tuberculosis” and “Amhara National Regional State” into the document.

2. **Reviewer 5**: In table 1: the 95% CI level for unemployed is beyond the range 3(4.3, 7.3). Therefore, please remove 95% CI from the table if possible.

**Author response**: Thank you for this valuable comment. We carefully reviewed Table 1 and observed that the reported 95% confidence interval for the unemployed category was inconsistent with the presented values which occurs during typing. Accordingly, we revised the table to avoid potential misinterpretation.

3. **Reviewer 5**: In table 3: use the reference “rural” not urban. Because your focus is on prevalence. Also, for Table 5.

**Author response**: Thank you for the comment. We acknowledge that using “rural” residence as the reference category may better align with studies focusing on prevalence comparisons. However, in our analysis, we intentionally used “urban” residence as the reference category because we considered urban residents to have comparatively lower exposure and lower risk of tuberculosis due to better access to healthcare services, health information, and living conditions. Therefore, the comparison was made against the group presumed to be less affected by tuberculosis.

4. **Reviewer 5**: In table 3: illiterate is not logical word. Use “no formal education”

**Author response**: Thank you for the valuable comment. We accept the suggestion and have revised Table 3 by replacing the term “illiterate” with “no formal education” throughout the document.

Finally, once again we would like to thank you the editor and reviewers for your time and expertise.

In case of any questions and doubts, please do not hesitate to contact us anytime.

With best regards,

Tebelay Dilnessa (MSc, PhD candidate in Medical Microbiology, University of Gondar,

Email: [tebelay@gmail.com](mailto:tebelay@gmail.com); Telephone: +251912198715)

Onbehaf of all authors
