# Supplementary material for: Rifampicin resistance and associated factors of Mycobacterium tuberculosis among pulmonary tuberculosis-suspected patients in the Amhara National Regional State Comprehensive Specialized Hospitals, Ethiopia
Source: PLoS One. 2026 Jun 26;21(6):e0351964. doi: 10.1371/journal.pone.0351964 (PMC13309019; doi:10.1371/journal.pone.0351964)
Supplement: Peer review history

# Peer review: Decision Letter 0

**DOI:** 10.1371/journal.pone.0351964.r001

**Authors:** Balew Arega Negatie

29 Apr 2026

PONE-D-26-17510

Rifampicin resistance and associated factors of Mycobacterium tuberculosis among pulmonary tuberculosis-suspected patients in the Amhara National Regional State Comprehensive Specialized Hospitals, Ethiopia

PLOS One

Dear Dr. Dilnessa ,

Thank you for submitting your manuscript to PLOS ONE. After careful consideration, we feel that it has merit but does not fully meet PLOS ONE’s publication criteria as it currently stands. Therefore, we invite you to submit a revised version of the manuscript that addresses the points raised during the review process.

**ACADEMIC EDITOR:**

- Attach the reviewer and editor comments as comments in a separate document, and use tracked changes in the main document. Carefully consider each comment, and either correct or justify accordingly in your revision

-->Please submit your revised manuscript by Jun 13 2026 11:59PM. If you will need more time than this to complete your revisions, please reply to this message or contact the journal office at plosone@plos.org. When you're ready to submit your revision, log on to https://www.editorialmanager.com/pone/ and select the 'Submissions Needing Revision' folder to locate your manuscript file.

Please include the following items when submitting your revised manuscript:

- A letter that responds to each point raised by the academic editor and reviewer(s). You should upload this letter as a separate file labeled 'Response to Reviewers'.
- A marked-up copy of your manuscript that highlights changes made to the original version. You should upload this as a separate file labeled 'Revised Manuscript with Track Changes'.
- An unmarked version of your revised paper without tracked changes. You should upload this as a separate file labeled 'Manuscript'.

We look forward to receiving your revised manuscript.

Kind regards,

Balew Arega Negatie, Msc,MD

Academic Editor

PLOS One

Journal Requirements:

When submitting your revision, we need you to address these additional requirements.

1. Please ensure that your manuscript meets PLOS ONE's style requirements, including those for file naming. The PLOS ONE style templates can be found at

https://journals.plos.org/plosone/s/file?id=wjVg/PLOSOne_formatting_sample_main_body.pdf and

https://journals.plos.org/plosone/s/file?id=ba62/PLOSOne_formatting_sample_title_authors_affiliations.pdf

2. We note that there is identifying data in the Supporting Information file "S1 Data.xlsx_0". Due to the inclusion of these potentially identifying data, we have removed this file from your file inventory. Prior to sharing human research participant data, authors should consult with an ethics committee to ensure data are shared in accordance with participant consent and all applicable local laws.

Data sharing should never compromise participant privacy. It is therefore not appropriate to publicly share personally identifiable data on human research participants. The following are examples of data that should not be shared:

-Name, initials, physical address

-Ages more specific than whole numbers

-Internet protocol (IP) address

-Specific dates (birth dates, death dates, examination dates, etc.)

-Contact information such as phone number or email address

-Location data

-ID numbers that seem specific (long numbers, include initials, titled “Hospital ID”) rather than random (small numbers in numerical order)

Data that are not directly identifying may also be inappropriate to share, as in combination they can become identifying. For example, data collected from a small group of participants, vulnerable populations, or private groups should not be shared if they involve indirect identifiers (such as sex, ethnicity, location, etc.) that may risk the identification of study participants.

Additional guidance on preparing raw data for publication can be found in our Data Policy (https://journals.plos.org/plosone/s/data-availability#loc-human-research-participant-data-and-other-sensitive-data) and in the following article: http://www.bmj.com/content/340/bmj.c181.long.

3. Please remove or anonymize all personal information sex and age, ensure that the data shared are in accordance with participant consent, and re-upload a fully anonymized data set. Please note that spreadsheet columns with personal information must be removed and not hidden as all hidden columns will appear in the published file.

If the reviewer comments include a recommendation to cite specific previously published works, please review and evaluate these publications to determine whether they are relevant and should be cited. There is no requirement to cite these works unless the editor has indicated otherwise.

Reviewers' comments:

Reviewer's Responses to Questions

**Comments to the Author**

1. Is the manuscript technically sound, and do the data support the conclusions?

The manuscript must describe a technically sound piece of scientific research with data that supports the conclusions. Experiments must have been conducted rigorously, with appropriate controls, replication, and sample sizes. The conclusions must be drawn appropriately based on the data presented.

Reviewer #1: Yes

Reviewer #2: Yes

Reviewer #3: Yes

Reviewer #4: Yes

**********

2. Has the statistical analysis been performed appropriately and rigorously?

Reviewer #1: Yes

Reviewer #2: Yes

Reviewer #3: Yes

Reviewer #4: No

**********

3. Have the authors made all data underlying the findings in their manuscript fully available?

The PLOS Data policy requires authors to make all data underlying the findings described in their manuscript fully available without restriction, with rare exception (please refer to the Data Availability Statement in the manuscript PDF file). The data should be provided as part of the manuscript or its supporting information, or deposited to a public repository. For example, in addition to summary statistics, the data points behind means, medians and variance measures should be available. If there are restrictions on publicly sharing data—e.g. participant privacy or use of data from a third party—those must be specified.

Reviewer #1: Yes

Reviewer #2: Yes

Reviewer #3: Yes

Reviewer #4: Yes

**********

4. Is the manuscript presented in an intelligible fashion and written in standard English?

PLOS ONE does not copyedit accepted manuscripts, so the language in submitted articles must be clear, correct, and unambiguous. Any typographical or grammatical errors should be corrected at revision, so please note any specific errors here.

Reviewer #1: Yes

Reviewer #2: Yes

Reviewer #3: Yes

Reviewer #4: Yes

**********

-->5 Reviewers  comments

**Reviewer #1:**

This study emphasizes the prevalence of tuberculosis among the general population of the Amhara Region, Northwest Ethiopia. The topic is highly interesting and of public health concern. The manuscript was well prepared and clearly articulated. However, very minor modifications are needed.

- It would be better to see the prevalence of rifampicin resistance among the overall suspected cases.
- In Table 3, the variable stated as "others" under occupation is significantly associated. Don't you think this will be difficult to discuss?
- The title of Table 4 needs a slight modification. The word "associated factors" is a little vague. Please change to "clinical data." (Line No. 266)

**Reviewer #2:**

The overall comments are attached to the system. The manuscript is technically sound, and the analysis is well done. Moreover, the manuscript is presented in an intelligible fashion and written in standard English, except for some editorial errors, unnecessary details, and some repetitions in the main document. Besides, the manuscript is comprehensive as it has been conducted using a multicenter approach.

**Reviewer #3:**

This manuscript presents good work addressing the current global issue of antimicrobial resistance. The topic is timely and relevant, and the study contributes valuable information to the field.

Please refer to my detailed comments and suggestions provided in the attached PDF.

**Reviewer #4:**

The manuscript is generally well organized; however, several aspects require revision to enhance its scientific rigor, clarity, and overall quality. Specifically, the methods and results sections need revision.

**********

[NOTE: If reviewer comments were submitted as an attachment file, they will be attached to this email and accessible via the submission site. Please log into your account, locate the manuscript record, and check for the action link "View Attachments". If this link does not appear, there are no attachment files.]

To ensure your figures meet our technical requirements, please review our figure guidelines: https://journals.plos.org/plosone/s/figures

You may also use PLOS’s free figure tool, NAAS, to help you prepare publication quality figures: https://journals.plos.org/plosone/s/figures#loc-tools-for-figure-preparation.

NAAS will assess whether your figures meet our technical requirements by comparing each figure against our figure specifications.

Attachment Submitted filename: Comments and questions TD Manuscript PONE-D-26-17510.docx

Attachment Submitted filename: Main manuscript track changes TD PONE-D-26-17510.docx

Attachment Submitted filename: PONE-D-26-17510.pdf

Attachment Submitted filename: Comments on Rifampicin resistance among MTB-2026.docx

Attachment Submitted filename: Manuscript.docx_editors.docx

# Author comment: Author response to Decision Letter 1

**DOI:** 10.1371/journal.pone.0351964.r002

9 May 2026

Response to reviewers

Dear Editor- in-Chief,

PLoS One

This is a revised version of the manuscript entitled "Rifampicin resistance and associated factors of Mycobacterium tuberculosis among pulmonary tuberculosis-suspected patients in the Amhara National Regional State Comprehensive Specialized Hospitals, Ethiopia” to be considered for publication in “PLoS One."

Dear Academic Editor,

We sincerely thank you for your decision and for the valuable detailed comments provided, which have been extremely helpful in improving our manuscript. We are also very grateful to the reviewers for their detailed, thoughtful, constructive comments and questions. Please find the attached revised version of the manuscript, which has been carefully updated in accordance with the comments and suggestions from the editor and reviewers. All comments from the tracked and other attached files have been carefully considered and incorporated into the manuscript. Where appropriate, we have also included brief explanations addressing the concerns raised by the reviewers. In addition, the entire manuscript has been thoroughly proofread to improve clarity, grammar, and overall presentation. We believe that these revisions have significantly strengthened the quality of the manuscript, and we hope that the revised version is now suitable for publication in your esteemed journal.

Responses to Academic Editor

1. Editor: Attach the reviewer and editor comments as comments in a separate document, and use tracked changes in the main document. Carefully consider each comment, and either correct or justify accordingly in your revision

Author response: We are grateful to you for your decision and to the reviewers for the time and effort invested in thoroughly evaluating our work and for providing valuable and constructive comments. We have carefully reviewed all the comments and suggestions raised by the reviewers and have revised the manuscript accordingly. Every concern has been addressed, and the recommended changes have been fully incorporated into the revised version of the manuscript. A detailed, point-by-point response to your comments and each reviewer’s comments is provided below.

2. Editor: Please indicate here whether you use secondary data or not

Author response: This study utilized primary data collected prospectively, as clearly described in the manuscript. Now additional clarification was included both the abstract and main sections of methods part.

3. Editor: Specifically mention, what is the outcome, there is confusing, either PTB or RR-MTB

Author response: Thank you for this important comment. In this study, both pulmonary tuberculosis (PTB) and RR-MTB were considered as outcome variables. PTB was defined as the primary outcome (presence of M. tuberculosis among suspected patients), while RR-MTB was treated as a secondary outcome among confirmed TB cases. In the revised manuscript, this distinction has been clearly stated in the methods and results sections to avoid any confusion. Associated factor analysis was also performed for RR-MTB.

4. Editor: It is less or equal

Author response: Thank you for the clarification: Yes, it is less or equal to 0.05 based on the statistical association principle.

5. Editor: It is multivariable not multivariate

Author response. We accepted the comment and uniformly corrected it, as it was ‘bivariable and multivariable.'

6. Editor: Be consistent; make it patient.

Author response: We made consistent by using patient rather than participant throughout the document.

7. Editor: Please remove this comparative word and just you can discuss in the discussion part

Author response: Thank you for the comment. We removed comparative words from the result part, and comparison was performed in the discussion part.

8. Editor: In your objective, you plan to determine the risk factors for RIF but here you determine risk factors for PTB. Please make it clear.

Author response: Thank you for the important comment. Both PTB and RR-MTB were considered in the analysis. Risk factors for PTB were assessed among all suspected patients, while risk factors for RR-MTB were evaluated among confirmed TB cases. This distinction has now been clarified, and the objective and corresponding sections have been revised to clearly indicate that factors associated with both PTB and RR-MTB were investigated in the manuscript (Tables 3, 4 & 5).

9. Editor: Unnecessary associated factors variables was not important in the abstract.

Author response: We removed those factors that lacked association with PTB and or RR-MTB and primary focus was given for those variables which have significant associations.

10. Editor: You can Make it “Rural residence (AOR: 0.23; 95% CI: 0.06-0.79) and being a student (AOR: 0.03; 95% CI: 0.01-0.11) were associated with significantly lower odds of pulmonary tuberculosis (PTB) infection. In contrast, previous antibiotic use (AOR: 9.17; 95% CI: 2.01-28.14) and alcohol consumption (AOR: 7.10; 95% CI: 4.99-20.86) were independently associated with increased M. tuberculosis infection.” If your plan was to determine factor for PTB

Author response: Thank you for the suggestion. We accepted the recommendation, and the revision was performed in the abstract.

11. Editor: You mention several studies but your reference is single i.e [9]

Author response: Thank you for this observation. We acknowledge that the initial version cited only a single reference despite referring to multiple studies. In the revised manuscript, we have incorporated additional up-to-date and relevant peer-reviewed references to appropriately support each statement and ensure consistency between the cited evidence and the discussion.

12. Editor: Please describe shortly their finding, their limitation and your current study value.

Author response:

13. Editor: Cross-check references, showing the regional variation of PTB or RR-TB across the region.

Author response: We checked the references that described the regional variations of PTB in Ethiopia.

14. Editor: The introduction last sentence was different from your objective in your abstract part, there, the risk factor is for RR-PTB

Author response: Thank you for this important observation. In the revised manuscript, we have aligned the final sentence of the introduction with the objective stated in the abstract. Specifically, we clarified that the study aims to assess the associated factors for both PTB and RR-MTB, rather than limiting the focus only to RR-PTB. This revision ensures consistency between the introduction and abstract and more accurately reflects the scope of our analysis.

15. Editor: Here add, how many specialized hospitals found in the region, how many % of them included in this study? Are these hospitals, treat MDR-TB patient?

Author response: There are 8 comprehensive specialized hospitals, namely, the University of Gondar, Felege Hiwot, Tibebe Ghion, Debre Markos, Debre Birhan, Dessie, Debre Tabor, and Woldia. All of these hospitals provide diagnoses of TB and rifampicin resistance and standard TB treatment, but four comprehensive specialized hospitals (UoG, FH, DM and Woldiya) are dedicated for treatment of MDR-TB. Three hospitals were included in the current study, representing 3 out of 8 (37.5%) of the comprehensive specialized hospitals in the region, and were considered sufficient to represent the entire region based on statistical sampling principles.

16. Editor: Why you excluded patients less than 8 years?

Author response: Thank you for this observation. Children under 8 years are often excluded primarily due to inability to produce sputum, low diagnostic yield, and difficulty in confirming bacteriological outcomes, which are critical for studies assessing TB and drug resistance patterns.

17. Editor: PTB suspected: take these to operational definition and add reference

Author response: We took to the operational definitions and operationalized based on the comment.

18. Editor: Distance from home to the health facility: Have you collected this?

Author response: Yes, we collected and categorized as less or equal to 40 and greater than 40 km based on the face-to-face interview with participant.

19. Editor: Your study population was PTB, so, EXPTB is not under your population, so, should not include in exclusion criteria

Author response: Thank you for this important comment. We agree that since the study population was limited to PTB-suspected patients, extrapulmonary tuberculosis (EPTB) cases fall outside the defined population and should not be listed under the exclusion criteria. Accordingly, EPTB has been removed from the manuscript part of exclusion criteria.

20. Editor: 1. Did you use a sample size calculation formula, or did you simply include all patients who had GenXert for PTB in the selected hospital (which is better)? Why did you choose an equal sample size (50 PTB patients) when it should be based on proportion? 2. Clearly describe the data type (primary vs. secondary), data source, etc. 3. How did you select patients- randomly, systematically, or conveniently?

Author response: Thank you for these constructive comments. We have revised the manuscript accordingly and provide the following clarifications:

1. Sample size determination and allocation across sites: No formal sample size calculation was performed for this study. Instead, we included all eligible PTB- suspected patients who underwent GeneXpert testing during the study period using a consecutive approach. The decision to enroll an equal number of 50 M. tuberculosis-positive cases per site was intentional to ensure balanced representation and enable direct comparison across the three study areas. This approach was adopted in the context of a dissertation project, where equal group sizes also facilitate comparison with parallel study groups (e.g., comparison of PTB patients versus apparently healthy individuals in terms of diversity and abundance of microbiota, antimicrobial resistance of microbiota, carbapenemase production-encoding genes, and treatment outcomes of PTB patients), although that objective is not part of the present manuscript. Additionally, having comparable sample sizes per site allows site-specific prevalence estimates to be calculated more consistently for microbiota, treatment outcomes, etc. for the next parts of the dissertation.

2. Data type and data source: The study utilized primary data collected prospectively from PTB-suspected patients at the selected hospitals. Laboratory results, including GeneXpert outcomes, were obtained directly from routine diagnostic testing, and relevant socio-demographic and clinical information was collected using semi-structured data collection tools.

3. Sampling technique: A convenience sampling technique was employed. Specifically, consecutive eligible patients were enrolled at each site until 50 bacteriologically confirmed PTB-cases were obtained per facility. This approach ensured feasibility within the study period while maintaining comparability across sites.

21. Editor: Add PTB-suspected patients, RR-MTB, what is indeterminate RR-MTB and what can do for indeterminate as operational definition

Author response: We operationalized the PTB-suspected patients, RR-MTB, intermediate RR-MTB. We noted that repeat testing or confirmatory methods (e.g., line probe assay or culture-based drug susceptibility testing) are recommended in routine practice; for this study test reputation with additional sputum sample by GeneXpert was performed, but no change was observed in the result.

22. Editor: Socio-demographic data collection: Is mean by interview? or extracted from the secondary data from HMIS or a patient chart?

Author response: The data were collected prospectively by trained healthcare professionals. We clarify it by revising as ‘Socio-demographic and clinical data were collected using a semi-structured, pre-tested questionnaire administered through face-to-face interviews by trained healthcare personnel.'

23. Editor: These sentences should be rephrased. Sputum, not saliva is the sample; it is not matter of emphasis

Author response: Thank you; we revised it.

24. Editor: Bivariable logistic regression analysis was employed to examine the association between independent variables with tuberculosis or rifampicin resistance: for what or both?

Author response: Thank you very much for your valuable comment. We performed logistic regression analysis for both associated factors for prevalence of PTB and RR-MTB. A revision was performed to avoid confusion for readers in the methods section.

25. Editor: If you determine factor for both/or RR-MTB or PTB. The data interpretation became confusing

Author response: Thank you very much for your valuable comment. We prepared separate tables, and associated factor analyses were performed for each RR-MTB and PTB and no confusion would be created now (Tables 3, 4 & 5).

26. Editor: Have you taken written consent from the 2,548 patients?

Author response: Thank you very much for your valuable comment. Yes, we took written consent from each individual from the three sites 862 from Debre Markos, 904 from Bahir Dar and 782 from University of Gondar Comprehensive Specialized Hospitals. Six individuals participated in the administration of the consent and interview.

27. Editor: Once you abbreviate a word, use the abbreviation thereafter.

Author response: Thank you, we revised the manuscript thoroughly and corrected it.

28. Editor: Please use a consistent numbering format, number with % or only %, better to use percentages with number proportions. %(a/b) formatting throughout your document

Author response: Thank you. We revised and used consistent formatting in the use of absolute number and percentages.

29. Editor: You can merge the two tables into one compressive table. The 95%CI can be described some main variables like overall PTB prevalence and RR-MTB.

Author response: Thank you, we merged tables 1 and 2.

30. Editor: Have you collected, data regarding the TB type (new vs previous TB treatment history), because the prevalence differed between the two groups

Author response: Based on the inclusion criteria, only PTB-suspected newly diagnosed by the authors were included as study participants. All previous PTB-treated, default, and lost-to-follow-up patients were excluded.

31. Editor: Is your interest to determine factors for PTB infection or for the RR-MTB, a lot has been done for PTB

Author response: Thank you; our objective was to identify the factors associated with both PTB infection and RR-MTB. Separate tables, results in text, and corresponding interpretations were clearly presented for each outcome in the revised manuscript.

32. Editor: No need to interpret variable in the univariable regression, just list the variables with p<0.25

Author response: Thank you, a modification was performed, not to focus to interpret COR on the univariable logistic regression analysis.

33. Editor: If you change the reference value, you can describe the variable that increase, rather than preventing the risk. Complete the interpretation, you miss the income

Author response: Thank you for this valuable comment. We agree that the interpretation should be clearer when the reference category is changed. In our analysis, we used the lower-risk or less-affected group as the reference category to improve interpretability of the associations. Accordingly, the direction of effect has been described in relation to this reference group. The monthly income data was included in text.

34. Editor: Sex, family size and study area are not candidate in univariable because the p value > 0.25. Not candidate variable because the p value in 0.25

Author response: Thank you for the comment. As observed in table 3, the variables including sex, family size, and study area were not included in the multivariable logistic regression analysis because the p-value in the bivariable logistic regression (in COR) was less than 0.25. But the bivariable logistic regression result was included in the table but not in the multivariable logistic regression column of the table.

35. Editor: Please include the result of AOR, result who were candidate in COR

Author response: Thank you for this important comment. We would like to clarify that sex was as

Attachment Submitted filename: Response to Reviewers.docx

# Peer review: Decision Letter 1

**DOI:** 10.1371/journal.pone.0351964.r003

**Authors:** Balew Arega Negatie, Balew Arega Negatie

26 May 2026

PONE-D-26-17510R1

Rifampicin resistance and associated factors of Mycobacterium tuberculosis among pulmonary tuberculosis-suspected patients in the Amhara National Regional State Comprehensive Specialized Hospitals, Ethiopia

PLOS One

Dear Dr. Teblay,

Thank you for submitting your manuscript to PLOS ONE. After careful consideration, we feel that it has merit but does not fully meet PLOS ONE’s publication criteria as it currently stands. Therefore, we invite you to submit a revised version of the manuscript that addresses the points raised during the review process.

Editor

This version is significantly improved compared with the previous one; however, it still requires further revision before it can be considered for publication.

1. Several sections contain unnecessary detail, making the Results section lengthy and difficult to follow.

2. Important findings should be highlighted concisely, while detailed distributions can remain in the tables.

3. Consistent use of abbreviations, numbering formats, terminology, and statistical reporting throughout the manuscript is recommended.

4. The Methods section should be further summarized by removing unnecessary details and excessive subheadings to improve readability and flow.

For more details, please refer to the main document with tracked changes.

-->Please submit your revised manuscript by  Jul 10 2026 11:59PM. If you will need more time than this to complete your revisions, please reply to this message or contact the journal office at plosone@plos.org. When you're ready to submit your revision, log on to https://www.editorialmanager.com/pone/ and select the 'Submissions Needing Revision' folder to locate your manuscript file.

Please include the following items when submitting your revised manuscript:

- A letter that responds to each point raised by the academic editor and reviewer(s). You should upload this letter as a separate file labeled 'Response to Reviewers'.
- A marked-up copy of your manuscript that highlights changes made to the original version. You should upload this as a separate file labeled 'Revised Manuscript with Track Changes'.
- An unmarked version of your revised paper without tracked changes. You should upload this as a separate file labeled 'Manuscript'.

We look forward to receiving your revised manuscript.

Kind regards,

Balew Arega Negatie, Msc,MD

Academic Editor

PLOS One

Journal Requirements:

If the reviewer comments include a recommendation to cite specific previously published works, please review and evaluate these publications to determine whether they are relevant and should be cited. There is no requirement to cite these works unless the editor has indicated otherwise.

Additional Editor Comments:

Reviewer

1. Regarding key words please add:

• Tuberculosis, Amhara Region

2. In table 1: the 95% CI level for unemployed is beyond the range 3(4.3, 7.3). Therefore, please remove 95% CI from the table if possible.

3. In table 3: use the reference “rural” not urban. Because your focus is on prevalence. Also for Table 5.

4. In table 3: illiterate is not logical word. Use “no formal education”

[Note: HTML markup is below. Please do not edit.]

Reviewers' comments:

Reviewer's Responses to Questions

**Comments to the Author**

1. If the authors have adequately addressed your comments raised in a previous round of review and you feel that this manuscript is now acceptable for publication, you may indicate that here to bypass the “Comments to the Author” section, enter your conflict of interest statement in the “Confidential to Editor” section, and submit your "Accept" recommendation.

Reviewer #1: All comments have been addressed

Reviewer #2: All comments have been addressed

Reviewer #4: All comments have been addressed

Reviewer #5: All comments have been addressed

**********

2. Is the manuscript technically sound, and do the data support the conclusions?

The manuscript must describe a technically sound piece of scientific research with data that supports the conclusions. Experiments must have been conducted rigorously, with appropriate controls, replication, and sample sizes. The conclusions must be drawn appropriately based on the data presented.

Reviewer #1: Yes

Reviewer #2: Yes

Reviewer #4: Yes

Reviewer #5: Yes

**********

3. Has the statistical analysis been performed appropriately and rigorously?

Reviewer #1: Yes

Reviewer #2: Yes

Reviewer #4: Yes

Reviewer #5: Yes

**********

4. Have the authors made all data underlying the findings in their manuscript fully available?

The PLOS Data policy requires authors to make all data underlying the findings described in their manuscript fully available without restriction, with rare exception (please refer to the Data Availability Statement in the manuscript PDF file). The data should be provided as part of the manuscript or its supporting information, or deposited to a public repository. For example, in addition to summary statistics, the data points behind means, medians and variance measures should be available. If there are restrictions on publicly sharing data—e.g. participant privacy or use of data from a third party—those must be specified.

Reviewer #1: Yes

Reviewer #2: Yes

Reviewer #4: Yes

Reviewer #5: Yes

**********

5. Is the manuscript presented in an intelligible fashion and written in standard English?

PLOS ONE does not copyedit accepted manuscripts, so the language in submitted articles must be clear, correct, and unambiguous. Any typographical or grammatical errors should be corrected at revision, so please note any specific errors here.

Reviewer #1: Yes

Reviewer #2: Yes

Reviewer #4: Yes

Reviewer #5: Yes

**********

6. Review Comments to the Author

Please use the space provided to explain your answers to the questions above. You may also include additional comments for the author, including concerns about dual publication, research ethics, or publication ethics. (Please upload your review as an attachment if it exceeds 20,000 characters)

Reviewer #1: (No Response)

Reviewer #2: I believe and checked thoroughly that the authors addressed all the questions and comments requested to correct.

Reviewer #4: All of my comments were thoroughly and appropriately addressed. I sincerely thank the authors once again for their efforts in assessing the public health concern of antimicrobial resistance, including inducible and clindamycin resistance associated with pulmonary tuberculosis. The study contributes valuable insight into the growing global threat of antimicrobial drug resistance and highlights an important area of public health research.

I appreciate the authors’ commitment and hard work in improving the manuscript and addressing the reviewers’ concerns with care and clarity. I wish the authors continued success in their future research and contributions toward combating this worldwide challenge of antimicrobial resistance.

Reviewer #5: (No Response)

**********

7. PLOS authors have the option to publish the peer review history of their article (what does this mean?). If published, this will include your full peer review and any attached files.

If you choose “no”, your identity will remain anonymous but your review may still be made public.

**Do you want your identity to be public for this peer review?**  For information about this choice, including consent withdrawal, please see our Privacy Policy.

Reviewer #1: No

Reviewer #2: **Yes:** Dessie Tegegne

Reviewer #4: **Yes:** Abebe Fenta Niguse

Reviewer #5: **Yes:** Deresse Daka

**********

[NOTE: If reviewer comments were submitted as an attachment file, they will be attached to this email and accessible via the submission site. Please log into your account, locate the manuscript record, and check for the action link "View Attachments". If this link does not appear, there are no attachment files.]

To ensure your figures meet our technical requirements, please review our figure guidelines: https://journals.plos.org/plosone/s/figures

You may also use PLOS’s free figure tool, NAAS, to help you prepare publication quality figures: https://journals.plos.org/plosone/s/figures#loc-tools-for-figure-preparation.

NAAS will assess whether your figures meet our technical requirements by comparing each figure against our figure specifications.

Attachment Submitted filename: Regarding key words please add.docx

# Author comment: Author response to Decision Letter 2

**DOI:** 10.1371/journal.pone.0351964.r004

28 May 2026

Dear Editor- in-Chief,

PLoS One

This is a revised version of the manuscript entitled "Rifampicin resistance and associated factors of Mycobacterium tuberculosis among pulmonary tuberculosis-suspected patients in the Amhara National Regional State Comprehensive Specialized Hospitals, Ethiopia” to be considered for publication in “PLoS One."

Dear Academic Editor,

We sincerely thank you for your decision and for the valuable detailed comments provided, which have been extremely helpful in improving our manuscript. We are also very grateful to the reviewers for their detailed, thoughtful, constructive comments and questions. Please find the attached revised version of the manuscript, which has been carefully updated in accordance with the comments and suggestions from the editor and reviewers. All comments have been carefully considered and incorporated into the manuscript. In addition, the entire manuscript has been thoroughly proofread to improve clarity, grammar, and overall presentation.

• Journal requirements: If the reviewer comments include a recommendation to cite specific previously published works, please review and evaluate these publications to determine whether they are relevant and should be cited. There is no requirement to cite these works unless the editor has indicated otherwise.

• Author response: The suggested articles were carefully reviewed and evaluated for their relevance to the current study. Citations were incorporated where they were directly relevant and strengthened the scientific context and discussion of the manuscript. However, references were not included solely based on reviewer recommendation, in accordance with the journal’s citation policy.

Responses to Academic Editor

1. Editor: Several sections contain unnecessary detail, making the Results section lengthy and difficult to follow.

Author response: Thank you for this valuable comment. We agree that several parts of the Results section contained excessive detail, which affected the overall clarity and readability of the manuscript. Accordingly, we carefully revised and condensed the Results section by removing redundant information and presenting the findings more concisely to improve the logical flow and readability of the manuscript.

2. Editor: Important findings should be highlighted concisely, while detailed distributions can remain in the tables.

Author response: We agree that the major findings should be emphasized concisely in the text, while detailed distributions are more appropriately presented in the tables. Accordingly, we revised the manuscript carefully.

3. Editor: Consistent use of abbreviations, numbering formats, terminology, and statistical reporting throughout the manuscript is recommended.

Author response: We appreciate the editor’s comment. The manuscript was carefully proofread and revised to ensure consistent use of abbreviations, numbering formats, terminology, and statistical reporting throughout the document.

4. Editor: The Methods section should be further summarized by removing unnecessary details and excessive subheadings to improve readability and flow.

Author response: Thank you for this valuable comment. We agree that the Methods section contained excessive details and multiple subheadings that affected the overall readability and flow of the manuscript. Accordingly, we have revised and summarized the Methods section by removing unnecessary details, merging related subsections, and simplifying the presentation to improve clarity and readability.

Responses to Reviewer #5

Thank you, dear reviewer, for your time, comments, and questions to us for improvement of our manuscript. We accepted comments and incorporated them into the paper, and we gave some responses to your comments and questions below.

1. Reviewer 5: Regarding key words, please add: Tuberculosis, Amhara Region

Author response: Thank you for the comment. I accept the suggestion and have incorporated the keywords “Tuberculosis” and “Amhara National Regional State” into the document.

2. Reviewer 5: In table 1: the 95% CI level for unemployed is beyond the range 3(4.3, 7.3). Therefore, please remove 95% CI from the table if possible.

Author response: Thank you for this valuable comment. We carefully reviewed Table 1 and observed that the reported 95% confidence interval for the unemployed category was inconsistent with the presented values which occurs during typing. Accordingly, we revised the table to avoid potential misinterpretation.

3. Reviewer 5: In table 3: use the reference “rural” not urban. Because your focus is on prevalence. Also, for Table 5.

Author response: Thank you for the comment. We acknowledge that using “rural” residence as the reference category may better align with studies focusing on prevalence comparisons. However, in our analysis, we intentionally used “urban” residence as the reference category because we considered urban residents to have comparatively lower exposure and lower risk of tuberculosis due to better access to healthcare services, health information, and living conditions. Therefore, the comparison was made against the group presumed to be less affected by tuberculosis.

4. Reviewer 5: In table 3, "illiterate" is not a logical word. Use “no formal education."

Author response: Thank you for the valuable comment. We accept the suggestion and have revised Table 3 by replacing the term “illiterate” with “no formal education” throughout the document.

Finally, once again, we would like to thank you, the editor and reviewers, for your time and expertise.

In case of any questions and doubts, please do not hesitate to contact us anytime.

With best regards,

Tebelay Dilnessa (MSc, PhD candidate in Medical Microbiology, University of Gondar,

Email: tebelay@gmail.com; Telephone: +251912198715)

Onbehaf of all authors

Attachment Submitted filename: Response_to_Reviewers_auresp_2.docx

# Editor report: Decision Letter 2

**DOI:** 10.1371/journal.pone.0351964.r005

**Authors:** Balew Arega Negatie, Balew Arega Negatie, Balew Arega Negatie

4 Jun 2026

Rifampicin resistance and associated factors of Mycobacterium tuberculosis among pulmonary tuberculosis-suspected patients in the Amhara National Regional State Comprehensive Specialized Hospitals, Ethiopia

PONE-D-26-17510R2

Dear Dr.Teblay

We’re pleased to inform you that your manuscript has been judged scientifically suitable for publication and will be formally accepted for publication once it meets all outstanding technical requirements.

Within one week, you’ll receive an e-mail detailing the required amendments. When these have been addressed, you’ll receive a formal acceptance letter and your manuscript will be scheduled for publication.

An invoice will be generated when your article is formally accepted. Please note, if your institution has a publishing partnership with PLOS and your article meets the relevant criteria, all or part of your publication costs will be covered. Please make sure your user information is up-to-date by logging into Editorial Manager at Editorial Manager® and clicking the ‘Update My Information' link at the top of the page. For questions related to billing, please contact billing support.

If your institution or institutions have a press office, please notify them about your upcoming paper to help maximize its impact. If they’ll be preparing press materials, please inform our press team as soon as possible -- no later than 48 hours after receiving the formal acceptance. Your manuscript will remain under strict press embargo until 2 pm Eastern Time on the date of publication. For more information, please contact onepress@plos.org.

Kind regards,

Balew Arega Negatie, Msc,MD

Academic Editor

PLOS One

Additional Editor Comments (optional):

Reviewers' comments:

# Editor report: Acceptance letter

**DOI:** 10.1371/journal.pone.0351964.r006

**Authors:** Balew Arega Negatie, Balew Arega Negatie, Balew Arega Negatie

PONE-D-26-17510R2

PLOS One

Dear Dr. Dilnessa ,

I'm pleased to inform you that your manuscript has been deemed suitable for publication in PLOS One. Congratulations! Your manuscript is now being handed over to our production team.

At this stage, our production department will prepare your paper for publication. This includes ensuring the following:

* All references, tables, and figures are properly cited

* All relevant supporting information is included in the manuscript submission,

* There are no issues that prevent the paper from being properly typeset

You will receive further instructions from the production team, including instructions on how to review your proof when it is ready. Please keep in mind that we are working through a large volume of accepted articles, so please give us a few days to review your paper and let you know the next and final steps.

Lastly, if your institution or institutions have a press office, please let them know about your upcoming paper now to help maximize its impact. If they'll be preparing press materials, please inform our press team within the next 48 hours. Your manuscript will remain under strict press embargo until 2 pm Eastern Time on the date of publication. For more information, please contact onepress@plos.org.

You will receive an invoice from PLOS for your publication fee after your manuscript has reached the completed accept phase. If you receive an email requesting payment before acceptance or for any other service, this may be a phishing scheme. Learn how to identify phishing emails and protect your accounts at https://explore.plos.org/phishing.

If we can help with anything else, please email us at customercare@plos.org.

Thank you for submitting your work to PLOS ONE and supporting open access.

Kind regards,

PLOS ONE Editorial Office Staff

on behalf of

Dr. Balew Arega Negatie

Academic Editor

PLOS One
